# Genome‐wide evidence for divergent selection between populations of a major agricultural pathogen

**DOI:** 10.1111/mec.14711

**Published:** 2018-05-23

**Authors:** Fanny E. Hartmann, Bruce A. McDonald, Daniel Croll

**Affiliations:** ^1^ Plant Pathology Institute of Integrative Biology ETH Zurich Zurich Switzerland; ^2^ Ecologie Systématique Evolution Univ. Paris‐Sud, AgroParisTech CNRS Université Paris‐Saclay Orsay France; ^3^ Laboratory of Evolutionary Genetics Institute of Biology University of Neuchâtel Neuchâtel Switzerland

**Keywords:** adaptation, ecological genetics, fungi, molecular evolution, natural selection and contemporary evolution, population genetics—empirical

## Abstract

The genetic and environmental homogeneity in agricultural ecosystems is thought to impose strong and uniform selection pressures. However, the impact of this selection on plant pathogen genomes remains largely unknown. We aimed to identify the proportion of the genome and the specific gene functions under positive selection in populations of the fungal wheat pathogen *Zymoseptoria tritici*. First, we performed genome scans in four field populations that were sampled from different continents and on distinct wheat cultivars to test which genomic regions are under recent selection. Based on extended haplotype homozygosity and composite likelihood ratio tests, we identified 384 and 81 selective sweeps affecting 4% and 0.5% of the 35 Mb core genome, respectively. We found differences both in the number and the position of selective sweeps across the genome between populations. Using a XtX‐based outlier detection approach, we identified 51 extremely divergent genomic regions between the allopatric populations, suggesting that divergent selection led to locally adapted pathogen populations. We performed an outlier detection analysis between two sympatric populations infecting two different wheat cultivars to identify evidence for host‐driven selection. Selective sweep regions harboured genes that are likely to play a role in successfully establishing host infections. We also identified secondary metabolite gene clusters and an enrichment in genes encoding transporter and protein localization functions. The latter gene functions mediate responses to environmental stress, including interactions with the host. The distinct gene functions under selection indicate that both local host genotypes and abiotic factors contributed to local adaptation.

## 1 INTRODUCTION

Agricultural plant pathogens are responsible for widespread epidemics and can quickly overcome control methods (McDonald & Stukenbrock, 2016). In particular, the breakdown of genetic resistance by pathogens can lead to significant yield losses and impact food security (Singh et al., 2011). Resistance to fungicides arises frequently, with significant economic costs for farmers (Hahn, 2014). These rapid evolutionary responses are largely due to the high environmental and genetic uniformity found in agricultural fields, which imposes strong directional selection that leads to the evolution of more virulent and drug‐resistant pathogen populations (Stukenbrock & McDonald, 2008). Despite abundant evidence for rapid evolution of pathogens in agricultural ecosystems, the loci involved in adaptive evolution remain largely unknown. Identifying loci under recent positive selection in populations can provide key insights into the mechanisms of adaptation (Weigel & Nordborg, 2015).

Signatures of positive selection were found in key loci functionally shown to play a role in the evolution of virulence, that is effector genes, and fungicide resistance in pathogen populations (Aguileta, Refrégier, Yockteng, Fournier, & Giraud, 2009). During the co‐evolution of hosts and pathogens, the host evolves defence mechanisms that target specific pathogen genotypes. In turn, the pathogen evolves strategies to escape recognition by the host and infect the host tissues (Jones & Dangl, 2006). Pathogen effector genes should be under particularly strong positive selection because expressing these genes can be highly detrimental or beneficial for the pathogen (de Jonge, Bolton, & Thomma, 2011; Presti et al., 2015). Strong directional selection was found to affect polymorphism in a small number of well‐characterized effector genes. These genes mostly encode small secreted proteins that are under selection to avoid recognition by the prevalent host genotypes (Dai, Jia, Correll, Wang, & Wang, 2010; Van de Wouw et al., 2010). A few genes encoding cell wall degrading enzymes and host‐specific toxins were also under positive selection in pathogen populations (Brunner, Torriani, Croll, Stukenbrock, & McDonald, 2013; McDonald, Oliver, Friesen, Brunner, & McDonald, 2013). The fixation of adaptive mutations in the mitochondrial cytochrome *b* gene and the *CYP51* gene contributed to strobilurin and azole fungicide resistance, respectively (Brunner, Stefanato, & Mcdonald, 2008; Brunner, Stefansson, Fountaine, Richina, & McDonald, 2015; Delmas et al., 2017; Estep et al., 2015; Pereira, McDonald, & Brunner, 2017; Torriani, Brunner, McDonald, & Sierotzki, 2009). Despite these examples, the proportion of the genome under positive selection and the functions of the loci in these selected regions remain largely unknown.

In contrast to the environmental and genetic uniformity present at the field scale, agricultural landscapes may be highly heterogeneous at the continental scale. Environmental heterogeneity in agricultural landscapes may also be created on a temporal scale through crop rotations. These spatial and temporal differences in environmental heterogeneity are likely to impose selection on the corresponding pathogen populations. Earlier analysis of global genotypic and phenotypic diversity in allopatric fungal populations indicated that divergent selection likely contributed to local adaptation (McDonald et al., 2013; Stefansson, McDonald, & Willi, 2013; Zhan & McDonald, 2011; Zhan, Stefanato, & McDonald, 2006; Zhan et al., 2005). Divergent selection can also be detected at a very local scale, with host genotypes or fungicide treatments selecting for adapted pathogen populations even within single fields (Cowger, Hoffer, & Mundt, 2000; Walker et al., 2017). Because similar agricultural practices can lead to similar environments (e.g., by planting genetically identical crops, applying the same fertilizers and spraying the same fungicides) for pathogen populations on different continents, there are opportunities for parallel adaptation affecting the same pathogen traits. However, it remains largely unknown whether the same loci will be affected in the same way by similar selection pressures applied in different regions (Croll & McDonald, 2016).

Genome‐wide signatures of recent selection can be detected using genome scans. Selective sweeps are detected based on changes in genetic diversity along chromosomes, with the power to detect sweeps resting largely on hitchhiking effects between an adaptive locus and proximal polymorphisms. Scans for divergence screen for extreme population differentiation at a small subset of the loci (Nielsen, 2005; Vitti, Grossman, & Sabeti, 2013). Genome scans were successfully applied to fungal populations found in natural ecosystems. Selective sweeps were found to affect between 1% and 17% of the genome in two sister species of the anther smut fungus, *Microbotryum lychnidis‐dioicae* and *M. silenes‐dioicae* (Badouin et al., 2017). The sweep regions contained several genes with pathogenicity‐related functions. A dominant force of selection was proposed to be adaptation to the host (Badouin et al., 2017). In other plant‐associated fungi, genome‐wide scans for divergent selection identified several outlier regions for population divergence along a gradient of abiotic environments that included salinity and temperature (Branco et al., 2015, 2017; Ellison et al., 2011). The genomes of fungal pathogens in agricultural ecosystems are likely to be similarly affected by selection due to a combination of biotic and abiotic factors, but there was no empirical evidence for this until now.

The fungus *Zymoseptoria tritici* is the most damaging wheat pathogen in Europe (Fones & Gurr, 2015). The fungus establishes itself first as an apparent biotroph on wheat leaves, then switches to necrotrophy after killing the host cells and finally lives as a saprotroph on the dead plant material. The fungus undergoes several cycles of sexual and asexual reproduction annually (Eyal, 1999). High levels of gene flow through airborne ascospore dispersal and frequent sexual reproduction maintain large effective population sizes, leading to a rapid decay in linkage disequilibrium (Croll, Lendenmann, Stewart, & McDonald, 2015; Zhan et al., 2005). Given these properties, we expect that *Z. tritici* populations should respond rapidly to selection pressures during the cropping season. Studies of field populations showed that the pathogen rapidly evolved resistance to fungicides and gained the ability to infect previously resistant hosts (Cowger et al., 2000; O'Driscoll, Kildea, Doohan, Spink, & Mullins, 2014). Recent studies based on quantitative trait loci (QTL) mapping identified candidate loci for fungicide resistance, melanization, temperature sensitivity and virulence (Lendenmann, Croll, & McDonald, 2015; Lendenmann, Croll, Palma‐Guerrero, Stewart, & McDonald, 2016; Lendenmann, Croll, Stewart, & McDonald, 2014; Mirzadi Gohari et al., 2015; Stewart et al., 2018). Genome‐wide association mapping showed that the pathogen overcame host resistance by mutations in genes encoding small secreted proteins (Hartmann, Sánchez‐Vallet, McDonald, & Croll, 2017; Zhong et al., 2017). Transcriptomic studies showed waves of expression of genes encoding secreted proteins (i.e., effectors, peptidases, peroxidases and cell wall degrading enzymes) and secondary metabolite production pathways during infection (Palma‐Guerrero et al., 2017; Rudd et al., 2015). In addition, analyses of synonymous versus nonsynonymous substitutions among *Z. tritici* and its wild relatives identified 27 positively selected genes (Stukenbrock et al., 2011). Three of these genes had an experimentally validated impact on virulence and reproduction during infection (Poppe, Dorsheimer, Happel, & Stukenbrock, 2015). A study using a similar strategy identified diversifying selection in six of 48 genes encoding plant cell wall degrading enzymes (Brunner et al., 2013). Although targeted analyses of loci segregating variation within the species and genes under positive selection among lineages identified candidate genes involved in adaptation, the genomic regions and corresponding genes under recent selection in populations of *Z. tritici* are unknown.

In this study, we aimed to identify the proportion of the genome and the specific gene functions under recent positive selection in populations of *Z. tritici*. We analysed whole‐genome data of 123 isolates from four field populations located on three continents and planted to different wheat cultivars. In Oregon (USA), isolates were from two sympatric populations collected on the same day from two different wheat cultivars planted in the same field (Croll, Zala, & McDonald, 2013; Hartmann & Croll, 2017; Hartmann et al., 2017; Torriani, Stukenbrock, Brunner, McDonald, & Croll, 2011; Zhan et al., 2005). Population structure was found to be consistent with the geographic origin of the isolate, with distinct environments likely leading to locally adapted pathogen populations based on selection for fungicide resistance, temperature sensitivity and virulence (Zhan et al., 2005, 2006). Individual populations harboured substantial genetic variation based on genome‐wide analyses of single nucleotide polymorphism (SNP) and gene content (Hartmann & Croll, 2017; Hartmann et al., 2017). Linkage disequilibrium decayed within 10 kb for all populations and within much less distance in the most polymorphic populations (Hartmann et al., 2017). First, we performed genome scans in the four allopatric populations to identify which genomic regions are under recent positive selection in field populations and to analyse whether the same or distinct genomic regions are under selection among populations. To determine what proportion of the genome was under selection, we first sought evidence for selective sweeps in the four allopatric field populations individually. Individual fields likely experienced a homogeneous biotic and abiotic environment. We used extended haplotype homozygosity (EHH) and composite likelihood ratio (CLR) tests. These are complementary methods that are designed to detect different types of selection signatures. The CLR test allows detection of hard sweeps, whereas iHS is more powerful for detecting incomplete sweeps (Pavlidis & Alachiotis 2017; Vitti et al., 2013). To identify the most likely gene functions under selection, we analysed the gene content of each selective sweep region. To identify whether some loci show evidence of divergent selection, we then analysed loci showing extreme population divergence based on the XtX‐based outlier detection method that incorporates both the population co‐ancestry and demography history. At last, we investigated host‐driven selection by analysing divergent selection in the pair of sympatric populations in Oregon. We found that about 5% of the *Z. tritici* genome showed signatures of positive selection. Selective sweep regions differed strongly between the allopatric populations and we identified several genomic regions showing extreme population divergence, providing evidence for divergent selection. The gene functions found in the selected regions indicate that both local host genotypes and abiotic factors contributed to local adaptation. The analyses of the two sympatric populations revealed genes likely to be involved in host adaptation.

## 2 MATERIALS AND METHODS

### 2.1 Fungal isolate collection and whole‐genome sequences

We analysed a total of 123 isolates of *Z. tritici* that were collected from naturally infected wheat fields between 1990 and 2001: Australia (*n* = 26), Israel (*n* = 24), Switzerland (*n* = 27) and Oregon, USA (*n* = 46). In each location, all isolates were collected from a single field and cultivar, except for the Oregon isolates that were collected from two different wheat cultivars, Madsen and Stephens, growing in the same field (Zhan et al., 2005). Whole‐genome sequencing data were generated for all 123 isolates (Croll et al., 2013; Hartmann & Croll, 2017; Hartmann et al., 2017; Torriani et al., 2011). In brief, high‐quality genomic DNA was extracted from liquid cultures and Illumina paired‐end sequencing of 100‐bp read length, and an insert size of ca. 500 bp was performed to generate 0.7–2.5 Gb sequence data per isolate. All Illumina sequence data are accessible from the NCBI Short Read Archive (for Accession nos, see Supporting information: Table S1).

### 2.2 Read mapping and variant calling procedure

Raw Illumina reads were trimmed for adapter contamination and sequencing quality. For trimming, the software trimmomatic v0.32 (Bolger, Lohse, & Usadel, 2014) was used with the following settings: illuminaclip = TruSeq3‐PE.fa:2:30:10, leading = 10, trailing = 10, slidingwindow = 5:10, minlen = 50. Trimmed Illumina reads were aligned to the reference genome IPO323 using the short read aligner bowtie 2 version 2.2.3 (Langmead, Trapnell, Pop, & Salzberg, 2009) with the following settings: –very‐sensitive‐local –phred33 –X 1,000. We used the reference genome assembly version 2 (Goodwin et al., 2011) from EnsemblFungi (Flicek et al., 2014). The MarkDuplicates module of Picard tools version 1.118 (http://broadinstitute.github.io/picard) was used to mark PCR duplicates in the alignment (bam) files. Average coverage ranged from 8× to 29× for all 123 isolates and no evidence of chromosomal aneuploidy was found (Hartmann & Croll, 2017).

Single nucleotide polymorphism (SNP) calling and variant filtration were performed using the Genome Analysis Toolkit (GATK) version 3.3‐0 (McKenna et al., 2010). First, we performed SNP calling for all 123 *Z. tritici* isolates independently using HaplotypeCaller with the following options: –emitRefConfidence GVCF; –variant_index_type LINEAR; –variant_index_parameter 128000; –sample_ploidy 1. Then, we used GenotypeGVCFs to perform joint variant calls on a merged gvcf variant file with the option –maxAltAlleles 2. We kept only SNPs from the joint variant call file. We used the GATK VariantFiltration and SelectVariants tools to perform hard filtering of SNPs based on quality cut‐offs following the GATK Best Practices recommendations (DePristo et al., 2011; Van der Auwera et al., 2002). We used the following cut‐offs: QUAL < 250; QD < 20.0; MQ < 30.0; –2 > BaseQRankSum > 2; –2 > MQRankSum > 2; –2 > ReadPosRankSum > 2; FS > 0.1. After filtration for genotyping rate (>90%), a set of 1,527,909 SNPs was retained. To further validate the identified SNPs, we used the independent SNP caller freebayes v.0.9 (Garrison & Marth, 2012). We set freebayes to ignore poorly mapped reads and poor base quality using the following stringent parameters: –min‐mapping‐quality 30; –min‐base‐quality 20; –use‐best‐n‐alleles 2; –min‐alternate‐count 1; –ploidy 1; –use‐mapping‐quality; –no‐indels; –no‐mnps; –no‐complex. SNPs were filtered for quality using the GATK VariantFiltration and SelectVariants tools (QUAL < 250). We retained 1,678,238 SNPs with a genotyping rate >90% from freebayes. To identify the most stringently called SNPs, we retained only SNPs called using HaplotypeCaller and freebayes using the option –diff‐site of vcftools version 0.1.13 (Danecek et al., 2011). The overlap contained 1,456,070 SNPs (i.e., 83.2% of all HaplotypeCaller SNPs; Supporting information: Figure S1A). The SNP quality (QUAL) and the alternative allele frequency were highly correlated (Supporting information: Figure S1B–S1D). Pearson's product‐moment correlation tests were performed using the open source software r. We excluded SNPs located on accessory chromosomes and tri‐allelic SNPs. After these additional filtering steps, we retained a total of 1,375,999 SNPs for all further analyses. We annotated and predicted the effect of SNPs using SnpEff 4.3i (Cingolani, Platts et al., 2012) and SnpSift (Cingolani, Patel et al. et al., 2012).

### 2.3 Assignment of ancestral and derived SNP alleles

To identify ancestral alleles at SNPs, we analysed whole‐genome sequencing data of the two closest known sister species of *Z. tritici*. We used raw Illumina reads of four *Z. pseudotritici* isolates (STIR04_2.2.1, STIR04_3.11.1, STIR04_5.3 and STIR04_5.9.1) and four *Z. ardabiliae* isolates (STIR04_1.1.1, STIR04_1.1.2, STIR04_3.13.1 and STIR04_3.3.2; Stukenbrock, Banke, Javan‐Nikkhah, & McDonald, 2007; Stukenbrock, Christiansen, Hansen, Dutheil, & Schierup, 2012). Procedures for read mapping and SNP calling were similar to those described above for *Z. tritici*. We modified the following trimmomatic settings to account for the older sequencing technology: leading=8 and trailing=8. Read alignment rates ranged from 44% to 49% for *Z. ardabiliae* and from 52% to 72% for *Z. pseudotritici*. We first performed SNP calling using the HaplotypeCaller of the Genome Analysis Toolkit (GATK) version 3.3‐0. Then, we repeated the SNP calling to include all *Z. tritici*,* Z. pseudotritici* and *Z. ardabiliae* isolates. SNPs were filtered for quality as described above. For each of the two *Z. tritici* sister species, we retained SNPs with a genotyping rate >50% and no intraspecific polymorphism. We assigned ancestral alleles for any *Z. tritici* SNPs if an identical allele was retained in both sister species. We were able to assign ancestral alleles for 584,327 SNPs (42% of the total number of *Z. tritici* SNPs).

### 2.4 Analyses of population structure

We analysed the population structure of all 123 *Zymoseptoria tritici* isolates using three methods. We performed a principal component analysis (PCA) based on all SNPs using the software tassel version 5.2.14 (Bradbury et al., 2007). We estimated population differentiation by calculating all pairwise *F*_ST_ fixation indices among populations (Wright, 1951). *F*_ST_ was calculated using the r package {hierfstats} (Goudet, 2005) that implements Yang's algorithm (Yang, 1998). Furthermore, we used the Bayesian unsupervised genetic clustering algorithm implemented in the software structure version 2.3.4 (Pritchard, Stephens, & Donnelly, 2000). We used an admixture model with correlated frequencies and no prior information about the population demography. The *K* parameter was tested for values ranging from 1 to 8 with 10 repetitions for each tested *K* value. We used 50,000 samples as a burn‐in period and 100,000 samples per run for the Monte Carlo Markov Chain (MCMC) replicates. Parameter convergence was inspected visually. Cluster assignment probabilities were computed using the clumpp program (Jakobsson & Rosenberg, 2007) and prepared for visualization using the distruct program (Rosenberg, 2004). For *F*_ST_ calculations and Bayesian genetic clustering, we selected a set of 2,047 genome‐wide equidistant SNPs at intervals of 15 kb along the chromosomes. This set of genome‐wide SNPs was assumed to be in linkage equilibrium based on previous estimates of linkage disequilibrium decay in *Z. tritici* populations (Croll et al., 2015; Hartmann et al., 2017).

### 2.5 Detection of selective sweeps

We detected selective sweeps using an extended haplotype homozygosity (EHH) and a composite likelihood ratio (CLR) tests. We performed each test for the four allopatric populations separately and included only SNPs with known ancestral states. For both tests, we used conservative percentile thresholds of the test statistics distribution to identify the strongest selective sweep regions. For the EHH test, we computed the integrated haplotype score (iHS) measure as implemented in the r package rehh (Gautier & Vitalis, 2012). The EHH corresponds to the decay of haplotype identity as a function of distance (Sabeti et al., 2007). Alleles favoured by positive selection are expected to be found on long haplotypes. The iHS statistic aims to detect abnormally long haplotype blocks by comparing the integrated EHH of the ancestral allele and the integrated EHH of the derived allele at each SNP. The method controls for heterogeneous recombination rates across the genome (Voight, Kudaravalli, Wen, & Pritchard, 2006). The 99.9th percentile of the distribution of absolute iHS values was used as a threshold for the detection of outlier SNPs. We clustered outlier SNPs in a set of selective sweep regions based on EHH variation around significant SNPs. Similar to Park et al. (2012), we computed windows around each core SNP based on a EHH decay threshold (Park et al., 2012). To be conservative, we computed windows around each core SNP where EHH decayed to 0.4 and grouped SNPs with windows overlapping on length >50%. Selective sweep region coordinates were based on the coordinates of the largest overlaps. As a second method, we performed the CLR test implemented in the software sweed v3.3.2 (Pavlidis, Živković, Stamatakis, & Alachiotis, 2013). sweed analyses the variation in the site‐frequency spectrum along the chromosome and implements the composite likelihood ratios (CLRs) test of SweepFinder (Nielsen, 2005). The CLR statistic computes the ratio of the likelihood of a selective sweep at a given position (referred to as grid point) to the likelihood of a null model without a selective sweep. The CLR statistic is robust to demographic events such as population expansions because the null model relies on the variation of the site‐frequency spectrum along the sequence of the whole genome or a full contig rather than the standard neutral model (Nielsen, 2005; Pavlidis et al., 2013). We calculated the CLR within each population and for each chromosome separately at grid points for every 1 kb. As low SNP density can lead to misleading CLR scores, we computed SNPs density in 50 kb nonoverlapping windows along chromosomes at the genome‐wide level. We retained only CLR values of grid points contained in 50 kb windows of at least 100 SNPs. The 99.5th percentile of the distribution of CLR scores was used as a threshold for the detection of outlier values. We selected distinct percentile values as significance threshold for CLR scores than iHS values because the two tests statistics showed different genomewide distributions. To define selective sweep regions in the Swiss, Israel and Oregon populations, we grouped adjacent grid points showing outlier CLR values into a single selective sweep region. Chromosome‐wide estimations of linkage disequilibrium decay were previously estimated to range from 0.6 to 2.2 kb in these three populations (Hartmann et al., 2017). Based on these estimations, we maintained separate sweep regions if the distance between grid points with outlier CLR scores was at least 5 kb. For each identified selective sweep region, we extended the region containing outlier CLR scores by adding 2.2 kb to each end to account for potential blocks of high linkage disequilibrium. As the Australian population showed slower linkage disequilibrium decay (decay to *r* ^2^ < 0.2 within 10.2 kb; Hartmann et al., 2017), we grouped grid points with outlier CLR scores if the grid points were <20 kb and extended selective sweep regions by adding 10 kb to each end. We considered selective sweep regions to be shared among populations and identified by both methods if the selective sweep regions overlapped by >50% in length. At last, we calculated the nucleotide diversity per site (π) and the Tajima's *D* (Tajima, 1989) statistic per gene using the popgenome r package (Pfeifer, Wittelsbürger, Ramos‐Onsins, & Lercher, 2014). We performed the analyses in the four *Z. tritici* populations separately using the entire SNP data set for each population. We calculated statistics only for genes containing at least 10 SNPs (i.e., 4,865, 9,299, 9,025 and 8,096 genes in the populations from Australia, Switzerland, Israel and Oregon, respectively).

### 2.6 Divergence tests and detection of outlier loci

To detect SNPs harbouring signatures of population divergence, we calculated the XtX statistic implemented in the program baypass (formerly bayenv) using the software default parameters (Gautier, 2015). We used all SNPs detected among the 123 isolates with a minor allele frequency of at least 0.05 (732,840 SNPs in total). A covariance matrix of allele frequencies was generated for all SNPs and used in the inference model to incorporate information on the shared demographic history of the populations (Coop, Witonsky, Rienzo, & Pritchard, 2010; Günther & Coop, 2013). To determine a significance threshold for loci under selection, we used the r function simulate.baypass() provided by baypass to generate a pseudoobserved data set of 1,000 SNPs following the specified inference model. We then computed the XtX statistics for this pseudoobserved data set. In addition, we extracted the XtX values for a set of 1,457 synonymous SNPs separated by at least 20 kb (Supporting information: Figure S5). As a conservative significance threshold, we used the maximum XtX value obtained for both data sets. In addition, we computed pairwise *F*_ST_ values in 1,000‐bp nonoverlapping windows for the entire set of SNP loci (Nei, 1973; Wright, 1951). We used the r package {hierfstat} (Goudet, 2005) that implements Yang's algorithm (Yang, 1998) to calculate *F*_ST_ values for each SNP locus. Average *F*_ST_ values per 1 kb nonoverlapping windows were calculated for windows that contained at least 10 SNPs. We used a 99.5‐percentile threshold to detect windows with high *F*_ST_ values. To investigate the presence of regions under positive selection in the two Oregon sympatric populations, we computed the XtX statistic as described above on a set of 571,265 polymorphic SNPs with minor allele frequency >0.05. We performed the cross‐population extended haplotype homozygosity (XP‐EHH) test (Sabeti et al., 2007) using the r package rehh. We used as an outlier detection threshold the 99.9th percentile of the distribution of absolute XP‐EHH values. For divergence scans, we clustered significant SNPs in separate genomic regions with high population differentiation if the distance between significant SNPs was at least 5 kb. This distance corresponds to the average distance of linkage disequilibrium decay (*r* ^2^ < 0.2) across the four populations.

### 2.7 Analysis of loci in candidate regions

Selective sweep regions identified by genome scans were analysed for their gene content using the updated gene models produced for the reference genome (Grandaubert, Bhattacharyya, & Stukenbrock, 2015). We used an in‐house pipeline to functionally annotate all genes. Protein family (PFAM) domain and gene ontology (GO) terms were assigned using interproscan v.5.16‐55.0 using default settings (Jones et al., 2014). Protein secretion signals were predicted using a combination of signalp v.4.1 (Petersen, Brunak, von Heijne, & Nielsen, 2011), phobius v.1.01 *(Käll, Krogh, & Sonnhammer,* 2007) and tmhmm v.2.0 (Krogh, Larsson, von Heijne, & Sonnhammer, 2001). The locations of transposable elements were retrieved from Grandaubert et al. (2015). The transcriptional profiles of individual genes were based on RNAseq data obtained from analyses of wheat seedling infections (Rudd et al., 2015). Rudd et al. (2015) measured gene transcript expression at five key stages of the wheat infection cycle (i.e., 1, 4, 9, 14 and 21 days postinfection). Expression level changes (X‐fold) were calculated as the ratio of the maximum value versus minimum value of reads per kilobase of transcript per million mapped reads (RPKM) during host infection.

We performed GO enrichment analyses for the genes located in selective sweep regions detected using the site‐frequency‐spectrum‐based method (sweed software) and the iHS based method (rehh software). We used the r packages {GSEABase} and {GOstats} (Falcon & Gentleman, 2007). The significance of enrichments was assessed using hypergeometric tests with a false discovery rate threshold of 0.05. We included only GO terms that were assigned to at least five different genes in the genome.

### 2.8 Linkage disequilibrium analyses

We analysed the linkage disequilibrium block structure in the selective sweep region affecting the polyketide synthase (PKS) 3 gene cluster on chromosome 5. For this, we used all SNPs with a minor allele frequency >0.1 in the Swiss, Israel and Oregon populations. We calculated the linkage disequilibrium *r* ^2^ between all marker pairs using the option –hap‐r2 in vcftools version 0.1.13 (Danecek et al., 2011). We produced a heatmap of pairwise linkage disequilibrium estimates using the function ldheatmap in the r package {LDheatmap} (Shin, Blay, Graham, & McNeney, 2006).

## 3 RESULTS

### 3.1 Widespread and narrow selective sweep regions in pathogen populations

**Figure 1:**
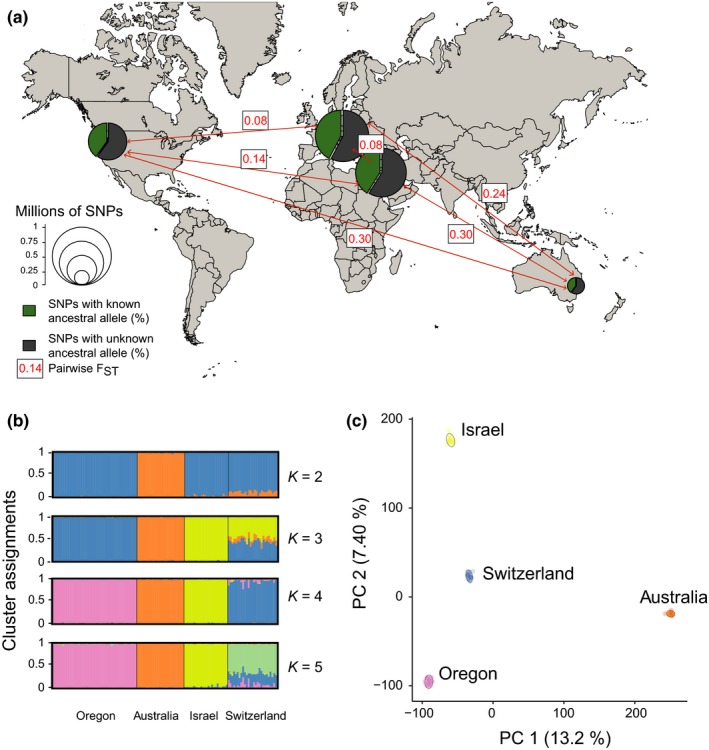
Sampling location, genetic polymorphism and population structure among 123 *Zymoseptoria tritici* isolates. (a) Sampling location and number of SNPs segregating within populations. The size of pie charts is proportional to the total number of SNPs, and the green slice of the pie indicates the percentage of SNPs with a determined ancestral allele. Arrows between pairs of populations show the pairwise *F*_ST_. (b) Clustering assignments of each genotype inferred using the software structure. Each colour represents one genetic cluster, and each vertical bar represents one isolate. The height of each colour in the vertical bar represents the posterior probabilities of cluster assignments. Isolates were ordered according to sampling location. (c) The first two principal components of a principal component analysis (PCA). The percentage of variance explained by each component is shown in parentheses. The sampling location of isolates is indicated [Colour figure can be viewed at http://wileyonlinelibrary.com]

We performed genome scans using whole‐genome sequence data generated for 123 *Z. tritici* isolates sampled from single wheat fields in Australia, Switzerland, Israel and Oregon, USA (Figure 1a). We detected a total of 1,375,999 high‐confidence, bi‐allelic SNP loci that were confirmed by two independent SNP callers. All analyses were restricted to SNPs on core chromosomes (i.e., chromosomes shared among all isolates; Supporting information: Figure S1). Population structure was consistent with the geographic origin of the isolates, and no field population showed evidence of intrapopulation genetic substructure (Figure 1b,c; Supporting information: Figure S2, Supporting information: Note S1). Overall, genetic diversity decreased with the distance from the pathogen centre of origin in the Fertile Crescent and the number of bottlenecks, consistent with previous studies (Figure 1a; Supporting information: Table S2; Supporting information: Figure S3; Zhan et al., 2005; Stukenbrock et al., 2007).

**Figure 2:**
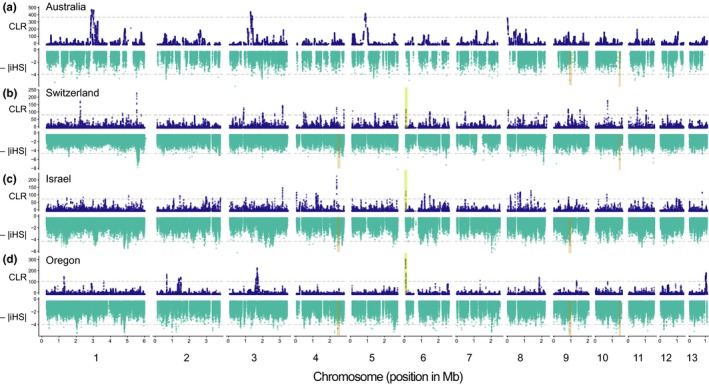
Genome‐wide analyses of selective sweeps in the four populations of *Zymoseptoria tritici*. In each panel, the upper plot represents the composite likelihood ratio (CLR) scores for windows at a distance of 1 kb computed using the CLR test implemented in sweed. The horizontal dashed line shows the 99.5% percentile threshold. The lower plot represents the absolute value (negatively transformed) of the integrated haplotype score (iHS) measure for each SNP locus with a known ancestral state. iHS values were calculated using the extended haplotype homozygosity (EHH) test implemented in the rehh package. The horizontal dashed line shows the 99.9% percentile threshold. Coloured rectangles highlight sweep regions detected by the CLR tests (yellow) and the EHH tests (orange) that were shared by three populations. Populations sampled in Australia (a), Switzerland (b), Israel (c) and Oregon (d) are shown separately [Colour figure can be viewed at http://wileyonlinelibrary.com]

To detect the proportion of the genome that experienced recent positive selection in *Z. tritici*, we first screened for signatures of selective sweeps in individual populations. For this, we restricted the analyses to SNPs with known ancestral state. Using whole‐genome sequence data of four and five strains from the two closely related sister species *Z. ardabiliae* and *Z. pseudotritici*, respectively, we were able to assign ancestral alleles for 584,327 SNPs (42.5%; Figure 1a; Supporting information: Table S2). Genome scans potentially produce high numbers of false positives as the demographic history of the population (i.e., population size variation and bottlenecks) can cause signatures resembling selective sweeps (Nielsen, 2005; Vitti et al., 2013). To limit the rate of false positives, we chose methods that incorporate genome‐wide patterns of genetic variation, used stringent percentile‐based thresholds for selective sweep detections and accurate linkage disequilibrium estimations to delimit sweep regions. We used two methods that are designed to detect different types of selective sweep signatures (Pavlidis & Alachiotis 2017). First, we performed an extended haplotype homozygosity (EHH) test. We computed the integrated haplotype score (iHS) statistic (Voight et al., 2006) using the algorithm implemented in the r package rehh (Table 1, Figure 2; Gautier & Vitalis, 2012). After clustering significant SNPs in regions of high extended haplotype homozygosity using conservative thresholds (EHH; |iHS_Switzerland_| > 4.29; |iHS_Israel_| > 4.04; |iHS_Oregon_| > 3.77), we identified a total of 90, 126 and 114 selective sweep regions in the Swiss, Israel and Oregon populations, respectively. Detected sweep regions were found on all 13 core chromosomes (Supporting information: Tables S4–S6). In the Australian population, we identified 54 selective sweep regions on chromosomes 1, 2, 3, 5, 6, 7, 8, 9, 10, 11 and 12 (|iHS_Australia_| > 3.71; Supporting information: Table S3). Sweep regions detected using the iHS statistic were on average 1.2 kb in length and covered a total of 4% of the core genome (Table 1). As a complementary method, we performed the composite likelihood ratio (CLR) test implemented in the software sweed. The software screens for local variation in the site‐frequency spectrum and calculates composite likelihood ratio (CLR) scores (Pavlidis et al., 2013; Table 1, Figure 2). Based on a conservative 99.5% outlier threshold (CLR_Australia_ > 389, CLR_Switzerland_ > 89.2; CLR_Israel_ > 82.8; CLR_Oregon_ > 120), we detected 6, 15, 29 and 31 regions affected by selective sweeps in the Australian, Oregon, Swiss and Israel populations, respectively (Supporting information: Tables S3–S6). Selective sweeps identified by sweed covered an average of 0.75% of the core genome in the four populations with individual regions ranging from 4.4 to 35.4 kb in length. Nucleotide diversity per site (π) and Tajima's *D* values were significantly lower for genes contained in selective sweeps identified by the CLR test than for genes not contained in selective sweeps (Supporting information: Figure S4). This is consistent with expectations for signatures produced by selective sweeps. However, we found no overall significant differences in diversity statistics for genes contained in selective sweeps identified by the EHH test, which may be due to the fact that EHH tests have higher sensitivities to detect incomplete sweeps.

**Table 1:** Summary statistics of the selective sweep regions identified in the four *Zymoseptoria tritici* populations using the haplotype‐based scan and the site‐frequency‐spectrum‐based scan

Sweep detection scan	Population	Number of sweep regions	Median length of sweep regions (kb)	Maximum length of sweep regions (kb)	Total length of regions affected by sweep		Number of genes in all sweep regions
					(kb)	(% core genome)	
Haplotype‐based scan	Australia	54	1	92.1	777.6	2.2	333
	Switzerland	90	1.4	105.9	759.3	2.2	295
	Israel	126	0.6	709.8	3201.4	9.1	1,172
	Oregon	114	1.7	84	1101.9	3.1	474
Site‐frequency‐spectrum‐based scan	Australia	6	32	78	253	0.7	87
	Switzerland	29	8.4	29.4	300.7	0.9	138
	Israel	31	6.4	32.4	294.5	0.8	138
	Oregon	15	11.4	35.4	206.1	0.6	57

### 3.2 Evidence of divergent selection among allopatric pathogen populations

To investigate whether the same loci were under selection between populations, we compared the location of selective sweep regions among the four allopatric populations. We considered selective sweep regions to be shared between populations if the selective sweep regions overlapped by >50% in length. Overall, selective sweep regions only weakly overlapped among populations using either of the two types of selection scans. Of 384 individual selective sweep regions detected in EHH tests, we found 140 regions (36.4%) to be shared among multiple populations. The majority of shared selective sweep regions were between two populations (86% of all overlapping regions). Selective sweep regions were shared among three populations on chromosomes 4 (2,556–2,557 kb), 9 (986–987 kb) and 10 (1,488–1,489 kb; Figure 2). Among the 81 selective sweep regions detected by CLR tests, only 11 regions overlapped among populations (13.6% of detected sweep regions). The strongest selective sweep region detected in the Oregon population was in the subtelomeric region of chromosome 6 (65–101 kb) and overlapped with selective sweep regions in the Israel (83–90 kb) and Swiss populations (82–101 kb). A total of eight selective sweep regions detected in the Swiss and Israel populations overlapped on chromosomes 3, 4 and 6. We found no overlap for any of the selective sweeps detected by the CLR test in the Australian population (Figure 2).

**Figure 3:**
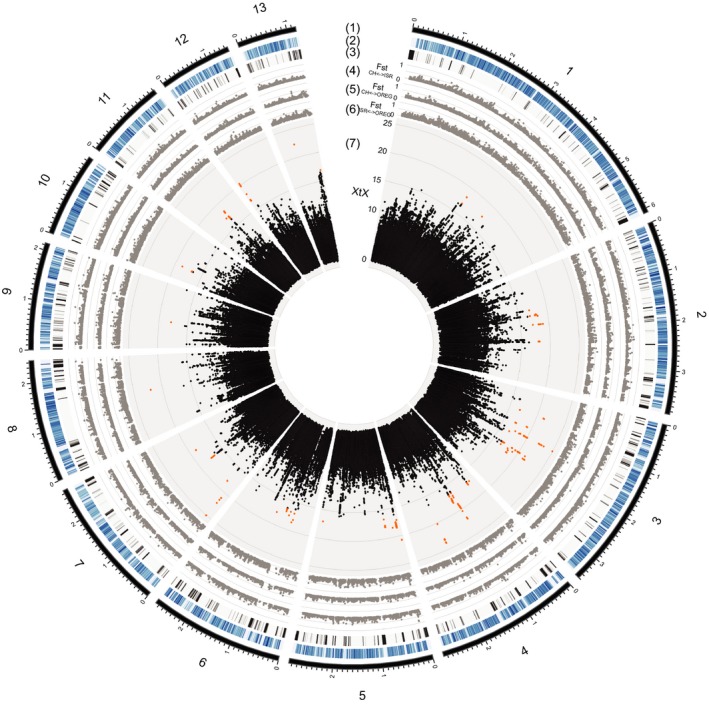
Genome‐wide scan for population divergence in the four populations of *Zymoseptoria tritici*. (1) Chromosomes of the reference genome and position in Mb. The core genome (i.e., shared among all strains) comprises 13 chromosomes. (2) Percentage of genes in 10 kb nonoverlapping windows (gradient shows differences from 0 to 100%). (3) Content of transposable element sequences in 10 kb nonoverlapping windows (gradient shows differences from 0% to 50%). (4–6) Mean values calculated in 1 kb nonoverlapping windows of pairwise *F*_ST_ for SNPs shared between the Swiss and Israel populations (4), the Swiss and Oregon populations (5), and the Israel and Oregon populations (6). (7) XtX statistics calculated at each SNP locus using the baypass software. The red dots correspond to outlier SNPs [Colour figure can be viewed at http://wileyonlinelibrary.com]

Given that populations showed predominantly population‐specific signatures of selection, we next analysed evidence for divergent selection among populations. For this, we calculated the XtX statistic implemented in the software baypass that incorporates both the population co‐ancestry and demography history (Gautier, 2015; Coop et al., 2010; Günther & Coop, 2013). The XtX statistic accounts for population structure by calculating a kinship matrix for a subsample of genome‐wide SNPs. We used all detected SNPs filtered for minor allele frequency of 5% (732,840 SNPs). We identified 390 SNPs that were outliers for population differentiation based on simulations and synonymous SNPs (XtX > 16.7; Supporting information: Figure S5). Highly differentiated SNPs clustered into 51 genomic regions distributed over all 13 core chromosomes (Figure 3; Supporting information: Table S7). As expected from the XtX statistics, outlier regions showed high *F*_ST_ values (the 99.5% outlier threshold was *F*_ST Switzerland vs Israel_ > 0.42, *F*_ST Switzerland vs Oregon_ > 0.38_,_ *F*_ST Israel vs Oregon_ > 0.57) in at least one pairwise population comparison (Figure 3).

**Figure 4:**
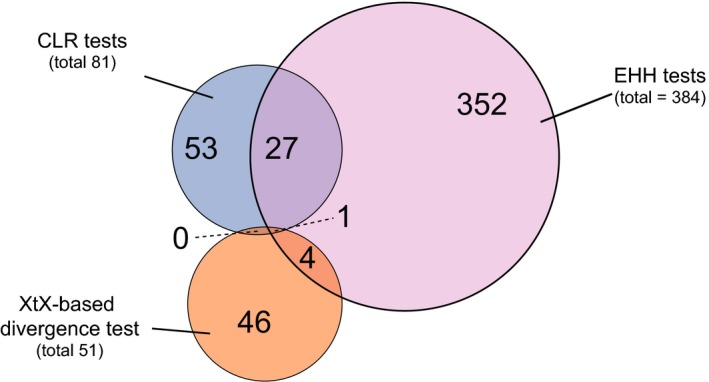
Venn diagram of overlaps between regions detected to be under positive selection by the composite likelihood ratio (CLR) test, the extended haplotype homozygosity (EHH) test and the XtX‐based outlier detection scan [Colour figure can be viewed at http://wileyonlinelibrary.com]

Overall, loci identified by different methods to detect positive selection showed little overlap (Figure 4). A total of 28 selective sweep regions overlapped among EHH and CLR tests. Among these overlapping regions, 12 selective sweep regions overlapped within populations, whereas an additional 16 selective sweeps detected by EHH in one population overlapped with sweep regions detected by CLR in another population. A total of 5 sweep regions detected by the EHH and CLR methods overlapped with regions with high differentiation between populations detected using the XtX statistic. Remarkably, the highly differentiated region located at 921.5–925.0 kb on chromosome 11 (XtX mean value = 17.2) overlapped with the selective sweep regions detected in the Israel population (918.0–923.4 kb) and the Oregon population (892.4–936.3 kb) using the CLR and EHH methods, respectively.

### 3.3 Gene content analyses of selective sweep regions

To identify the gene functions that were the target of recent selection, we analysed the function of genes within selective sweep regions. Detected selective sweep regions encompassed on average 337 genes per population and per scan (Table 1). The average number of genes within a selective sweep region was six genes. A total of 35.0% of genes in selective sweep regions did not encode a conserved protein family (PFAM) domain. We performed gene ontology (GO) enrichment analyses and tested for overrepresented GO terms compared to the genomic background for the genes with a PFAM domain. We found that GO terms for protein transport and localization functions were significantly overrepresented in selective sweep regions (*p*‐values = 0.005–0.04; Supporting information: Table S9). Selective sweep regions encoded 52 major facilitator superfamily (MFS) transporters. We found 54 additional genes with membrane transporter functions in selective sweep regions, including ATP‐binding cassette (ABC) transporters, sugar transporters and several metal and ion transporters.

Although not significantly enriched in function, selective sweep regions comprised 180 genes that encode secreted proteins including six plant cell wall degrading enzymes (CWDEs), 10 peptidases, five peroxidases and 23 small secreted proteins (SSPs) lacking conserved domains (Supporting information: Table S10). These gene categories were shown to be major virulence factors in plant pathogens including *Z. tritici* (de Jonge et al. 2012; Zhong et al., 2017; Hartmann et al., 2017). Genes encoding CWDEs included two cutinases (CEs family), two cellulases of the glycosyl hydrolase family GH5 and GH61 and two hemicellulases (GH43 and GH62 families). Three of the 23 genes encoding a SSP were highly upregulated during the infection (Rudd et al., 2015). The SSP‐encoding genes *3_00158, 5_00818* and *6_00224* had expression level changes during the infection equal to 375X, 859X and 114X, respectively. Three genes encoding SSPs and three genes encoding CWDEs were found in sweep regions that were shared among populations. Other genes encoding SSPs and CWDEs were found in population‐specific sweep regions. Among the genes in selective sweep regions that do not encode secreted proteins were four secondary metabolite gene clusters (SMGCs), including the polyketide synthase (PKS) genes PKS10 (*1_01966*), PKS3 (*5_00021*) and PKS2 (*9_00441*), and the nonribosomal peptide synthetase (NRPS) gene NRPS1 (*2_00117*; Ohm et al., 2012). The entire PKS10 SMGC was encompassed in a single selective sweep region. All SMGC genes were found in population‐specific sweep regions. Selective sweep regions also contained gene functions linked to general metabolism and regulation of transcription, including 109 genes encoding enzymes with dehydrogenase, hydrolase and oxidoreductase functions, and 35 genes encoding transcription factors.

**Figure 5:**
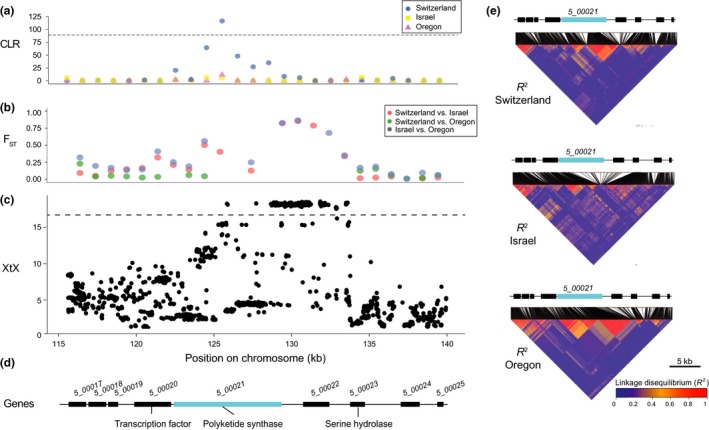
Signatures of divergent selection in the polyketide synthase (PKS) 3 gene cluster. (a) Composite likelihood ratio (CLR) scores for windows at a distance of 1 kb computed using the CLR test performed in sweed in the Swiss, Israel and Oregon populations. The horizontal dashed line shows the 99.5% percentile threshold of CLR values in the Swiss population. (b) Mean values calculated in 1 kb nonoverlapping windows of pairwise *F*_ST_ for SNPs shared between the Swiss and Israel populations (red), the Swiss and Oregon populations (green), and the Israel and Oregon populations (blue). (c) XtX statistics calculated at each SNP locus. The horizontal dashed line shows the maximum XtX statistics value obtained for a set of genome‐wide distributed synonymous SNPs. (d) Gene model of the PKS3 gene cluster in the 115–140 kb region. (e) Heatmap of pairwise linkage disequilibrium *r* ^2^ for SNPs with a minor allele frequency >0.1 in the Swiss, Israel and Oregon populations. Correspondence between SNPs location in the genomic region (kb) and on the heatmap is represented by black segments [Colour figure can be viewed at http://wileyonlinelibrary.com]

Over half of the SNPs (53%) identified through the divergence scan among the four populations were located in intergenic regions. The remaining SNPs were distributed across 36 different genes (Supporting information: Table S11). Eleven genes encoded a secreted protein, including four genes encoding SSPs that were highly upregulated during infection. The genes *2_00572, 3_0231, 3_00467* and *7_00040* encoded SSPs that had expression level changes equal to 18X, 741X, 4777X and 160X, respectively, during infection (Rudd et al., 2015). Remarkably, one outlier SNP was located in the major avirulence effector gene *AvrStb6* located on chromosome 5 at 69,206 bp (Zhong et al., 2017). Outlier SNPs were also found in three genes of the PKS3 gene cluster on chromosome 5, including the polyketide synthase (*5_00021)* and a MFS transporter gene (*5_00006;* Figure 5). A strong XtX outlier region was located on chromosome 5 (Figure 5c). This region showed also high *F*_ST_ values (>0.75) in the pairwise comparisons of Israel–Oregon and Israel–Switzerland (Figure 5b). A selective sweep of the PKS gene (*5_00021*) was also detected by the CLR test in the Swiss population (123.3–127.8 kb; Figure 5a). This region was also characterized by high levels of linkage disequilibrium (*r* ^2^ > 0.5) for pairs of SNPs at >10 kb distance in the Swiss, Israel and Oregon populations (Figure 5e), whereas linkage disequilibrium decayed generally within ~1–2.2 kb (Hartmann et al., 2017). The high levels of linkage disequilibrium are consistent with a recent selective sweep.

### 3.4 Evidence for host‐driven selection in sympatric pathogen populations

As the two sympatric Oregon populations were sampled on the same day from two different wheat cultivars, Madsen and Stephens (Zhan et al., 2005), growing in the same field, we investigated whether genotypes showed signatures of positive selection according to their cultivar of origin. We performed divergence scans using the XtX statistic but found no evidence for highly differentiated loci between populations (Supporting information: Figure S6). Using the cross‐population extended haplotype homozygosity (XP‐EHH) test (Sabeti et al., 2007) with a 99.9% outlier threshold, we identified 221 SNPs that were under positive selection only in one cultivar‐associated population but not the other. Outlier SNPs clustered in 12 genomic regions on chromosomes 1, 2, 6, 9 and 12 (Supporting information: Figure S7; Supporting information: Table S8). A total of 170 of 221 outlier SNPs were located in 16 distinct genes. These genes encoded proteins with either no conserved domain or functions linked to general metabolism and transcriptional regulation. The gene *9_00014* encoded a cysteine‐rich extracellular membrane protein with a CFEM domain (Supporting information: Table S12).

## 4 DISCUSSION

### 4.1 Complex signatures of divergent selection among populations

Using EHH and CLR tests in four allopatric populations of the major fungal wheat pathogen *Z. tritici*, we determined that about 5% (around 1 Mb) of the genome experienced recent positive selection. Selective sweep regions were narrow but widespread across the genome in all analysed populations. We chose very stringent thresholds for selective sweep detection to identify the strongest selective sweep regions. The number of detected selective sweeps would likely be higher by including all acceptable sweep regions based on demographic modelling. The extent of signatures of positive selection was consistent with studies in other fungi based on the same CLR tests. Genome scans in European populations of the anther smut fungus *Microbotryum*, a plant pathogen found in natural ecosystems, revealed that 1%–17% of the pathogen genome was affected by selective sweeps (Badouin et al., 2017). Branco et al. (2017) identified several hundred selective sweep regions in *S. brevipes* populations sampled across an environmental gradient in North America. Our and previous studies suggest that the number of selective sweeps detected in sexually reproducing fungal pathogen populations is higher than found in populations of mammals or plants (Evans et al., 2014; Qanbari et al. 2014; Bonhomme et al., 2015). These differences might be due to intrinsic population genetic properties of sexually reproducing fungal pathogens, such as high recombination rates and large population sizes that can accelerate the response to selection. Fungal populations with lower recombination rates will likely show less prevalent signatures of positive selection. The observed differences might also be due to the genetic architecture of pathogenicity traits and the strength of selection imposed on plant pathogens. In agroecosystems, the introduction of new host cultivars with different resistance mechanisms can rapidly alter selection pressures on pathogen populations. In addition, abiotic factors such as the application of fungicides, and fluctuations in temperature and humidity can alter selection pressures during the life cycle of a pathogen. Our findings were consistent with numerous soft sweeps caused by a variety of environmental factors. Largely due to their high effective population sizes, soft sweeps are thought to be dominant in rapidly evolving microorganisms, including plant pathogens (Delmas et al., 2017; Messer & Petrov, 2013).

We found little overlap between selective sweep regions detected by CLR and EHH tests. Some degree of discordance is expected because the methods are designed to detect different types of selection signatures (i.e., shifts in allele frequency spectra versus large haplotype block size). The CLR test allows detection of hard sweeps, whereas iHS is more powerful for detecting incomplete sweeps (Pavlidis & Alachiotis 2017; Vitti et al., 2013). Little overlap between genomic regions identified with these selection scan methods is common (Evans et al., 2014; Pavlidis & Alachiotis 2017). We identified selective sweep regions using both approaches because of their complementary nature. However, we chose very stringent percentile thresholds to detect selective sweeps and conservative estimates of linkage disequilibrium to delimit sweep regions. Due to the inherent limitations of inferring past selection through genome scans, understanding how genetic variation in individual genes contributes to adaptive trait variation ultimately requires experimental validation.

We found fewer selective sweeps in the less genetically diverse populations from Australia and Oregon (Hartmann et al., 2017; Zhan et al., 2005). Smaller population sizes and a slower decay in linkage disequilibrium are expected to make selection less efficient because beneficial mutations are more likely to be in linkage disequilibrium with slightly deleterious mutations. This is consistent with the smaller number of selective sweeps detected in populations with smaller effective population sizes. In addition to true differences in numbers of selective sweeps, the ability to detect selective sweeps can be affected by differences in the number of sampled individuals, levels of genetic diversity and the decay of linkage disequilibrium (Crisci, Poh, Mahajan, & Jensen, 2013). Although our study was based on genome scans that are robust to demographic histories of individual populations, some differences in selection signatures among populations may represent false positives.

We found differences in the number and position of selective sweeps across the *Z. tritici* genome among the four allopatric populations. Differences in power and demographic history may partly explain this pattern, although it is unlikely the only explanation given the extent of the observed heterogeneity. The identification of genomic regions with high population differentiation using the XtX test confirms that selection pressures were heterogeneous among these populations and favoured different alleles in different populations. We previously identified signatures of divergent selection at loci affected by copy number variation among these four populations (Hartmann & Croll, 2017). Such divergent selection is likely as the populations experienced differences in fungicide usage, annual mean temperatures and deployed host cultivars (Zhan & McDonald, 2011; Zhan et al., 2005, 2006). In natural ecosystems, fungal populations collected across ecological gradients also showed differences in the number and identity of loci under positive selection, which is strongly indicative of divergent selection (Branco et al., 2015, 2017; Ellison et al., 2011). Given sufficiently low levels of gene flow, divergent selection leads to locally adapted populations.

Evidence for local adaptation in plant‐colonizing fungi has mostly been found at the phenotypic level for adaptation to temperature and hosts (Branco et al., 2017), but the loci underlying local adaptation remain largely unknown (Croll & McDonald, 2016). Alternatives to local adaptation that can also lead to highly differentiated genomic regions include environment‐independent factors such as genetic incompatibilities due to prezygotic or postzygotic isolation, epistasis or underdominance. These factors often spatially coincide with ecological factors, leading to high differentiation among spatially structured populations (Bierne, Welch, Loire, Bonhomme, & David, 2011). As our system is haploid, allelic incompatibilities due to hybrid infertility, selection against hybrids, or underdominance cannot occur. However, epistasis among loci under selection may indeed exist and could interfere with the power to detect loci under positive selection. The fact that we analysed complete genomes instead of a reduced set of candidate loci provided significant power to identify novel adaptive loci. Analyses of putative functions showed that many loci in the selected regions were likely to encode functions linked to host and abiotic adaptation. In the end, analysing the contribution of individual loci to phenotypic traits will enable a clear distinction between loci underlying local adaptation and endogenous barriers (Hoban et al., 2016).

The two different hosts cultivated in sympatry in Oregon may have generated divergent selection pressures that favoured host specialization. It is interesting that the two sympatric populations were shown to differ in levels of fungicide resistance and aggressiveness on two other wheat cultivars, suggesting that the cultivars planted in Oregon indeed acted as an agent of divergent selection (Yang, Gao, Shang, Zhan, & McDonald, 2013; Zhan et al., 2005). However, the physical proximity of the two cultivars in the field and the ability of *Z. tritici* to disperse likely constrained the opportunity for local adaptation to either of the two host populations. We identified a set of genes in regions under positive selection in either of the sympatric populations. However, the functions of the identified genes are largely unknown.

### 4.2 Genes in recent selective sweeps are linked to adaptation to the host and to the abiotic environment

We identified a broad range of gene functions in selective sweep regions. The multitude of functions suggests that the fungal populations recently adapted both to local host genotypes and to abiotic factors. Selective sweep regions were not enriched in pathogenicity‐related functions. Nevertheless, we found genes that encode secreted proteins with functions in the degradation of the host cell wall or manipulation of host defences. Analyses of expression showed that many of these genes were strongly upregulated upon host infection. In *Z. tritici*, association mapping showed that mutations in two genes encoding SSPs (*8_609* and *AvrStb6*) were associated with host‐specific virulence (Hartmann et al., 2017; Zhong et al., 2017). These two genes were not found in selective sweep regions, which suggests that these populations were not experiencing recent positive selection by the host genotypes specifically associated with *8_609* and *AvrStb6*. Remarkably, *AvrStb6* was an outlier for divergent selection among populations in the XtX scan, which suggests that in at least one population selection on the *AvrStb6* locus may have been significant. *AvrStb6* encodes a protein recognized by the product of the host resistance gene *Stb6* and drastically reduces pathogen fitness on hosts carrying *Stb6* (Zhong et al., 2017). Several codons of the *AvrStb6* gene were shown to be under positive diversifying selection (Brunner & McDonald, 2018). We found no evidence for selective sweeps in the proximity of the second known host‐specific virulence gene *8_609*. The *8_609* locus is characterized by large chromosomal rearrangements that led to the adaptive deletion of the virulence gene (Hartmann et al., 2017). The structural variation at this locus may have had an impact on the power to detect evidence for positive selection. No other genes in regions under recent positive selection have yet been shown to play role in virulence. We found little overlap with genes identified to be under positive selection based on *dN*/*dS* ratios in the *Z. tritici* lineage compared to its sister species (Brunner et al., 2013; Stukenbrock et al., 2011), but two of six genes encoding plant cell wall degrading enzymes that were previously shown to be under diversifying selection (*2_00980* and *2_01151;* Brunner et al., 2013) were identified in the selected regions. As the genome scans in our study were designed to detect more recent selection than selection identified by amino acid substitution tests, the weak overlap among these different studies is not surprising (Vitti et al., 2013). For example, genes involved in the specialization of the pathogen on wheat are likely to be largely distinct from genes under more recent selection to adapt to individual wheat genotypes. Genome scans in populations of the *Microbotryum* plant pathogenic fungi also identified genes with putative roles in host adaptation, including genes upregulated during infection and encoding glycoside hydrolases, pectin lyases and extracellular membrane proteins with a cysteine‐rich CFEM domain (Badouin et al., 2017). Such gene functions form the pathogenesis toolkit of most plant pathogens (de Jonge et al., 2011; Presti et al., 2015; Rep, 2005). Combining evidence obtained from natural and agricultural ecosystems, host adaptation involving a wide range of gene functions appears to be a major driver of selection in populations of fungal plant pathogens.

Environmental factors are also likely to impose significant selection pressure (Stukenbrock & McDonald, 2009). Plant pathogens must cope with fluctuating temperatures and humidity, the host microbiota, variability in nutrients obtained from the host and agricultural practices (e.g., fertilizer and fungicide applications). This is reflected by the selective sweep regions that encompass genes with diverse functions that are most likely unrelated to host adaptation. In particular, we found an enrichment in transmembrane transport functions. Transmembrane transporters are involved in a wide range of cellular processes including obtaining nutrients during the infection (Chen et al., 2010), efflux of toxic compounds and fungicides (Del Sorbo, Schoonbeek, & De Waard, 2000), virulence (Wahl, Wippel, Goos, Kämper, & Sauer, 2010), environment sensing and stress responses (Bahn et al., 2007). Genome scans of populations of the root symbiont *S. brevipes* showed that adaptation to cold, salinity and water stress was most likely mediated by a series of transmembrane transporters (Branco et al., 2015, 2017). However, none of the transporter genes in the identified selective sweeps have yet been experimentally shown to affect adaptation in *Z. tritici*. Regions affected by selective sweeps also encoded proteins playing a role in regulatory pathways (e.g., transcription factors) and secondary metabolite production. The roles of secondary metabolites produced by *Z. tritici* remain poorly understood, but the clusters are likely to play roles in host adaptation, competition with microbes and environmental stress (Howlett, 2006). The PKS1 cluster is involved in melanin production in *Z. tritici* (Butler & Day, 1998; Lendenmann et al., 2014), but compounds produced by the four secondary metabolite clusters found in selective sweep regions remain unknown. These four clusters are upregulated at different stages of the infection process, suggesting distinctive roles in host colonization (Palma‐Guerrero et al., 2017). In addition to evidence for selection, the PKS9 cluster is affected by a large‐scale deletion polymorphism affecting the entire cluster (Hartmann & Croll, 2017). The polyketide synthase gene of the PKS3 cluster was an outlier for population differentiation and was affected by a selective sweep. This gene had a peak of expression during the early establishment of the infection and may be involved in the production of a toxin targeting the host (Palma‐Guerrero et al., 2017). Exposure to azole fungicides led to high levels of resistance in the Swiss population, but not in any other analysed population (Zhan et al., 2005). Therefore, sweeps due to selection for azole resistance should be restricted to the Swiss population. The *CYP51* gene encoding the target of azoles is indeed highly polymorphic in the Swiss population, with a large number of amino acid substitutions contributing to resistance (Brunner et al., 2008; Cools & Fraaije, 2013; Lendenmann et al., 2015). Although we found no direct evidence of positive selection in the *CYP51* locus, multiple SNPs had high XtX population differentiation values (>10). EHH and CLR tests may be unsuitable to detect the very rapid diversification into dozens of haplotypes that are typically observed at the *CYP51* locus.

Heterogeneity in both biotic and abiotic stresses creates the opportunity for local adaptation in plant pathogens (Croll & McDonald, 2016; Mboup et al., 2012). We found evidence for divergent selection among four allopatric populations with selection likely due to both abiotic factors and host resistance mechanisms. We also found evidence that different host genotypes in sympatry caused divergent selection pressure on the pathogen. The widespread evidence for selection across the genome suggests that the high effective population sizes of the pathogen strongly favoured rapid responses to divergent selection pressures. Loci under divergent selection among populations may ultimately constitute the genetic basis for local adaptation, but may also reveal constraints in the evolution of the pathogen. For example, selection for fungicide resistance is known to lead to correlated responses that negatively affect growth rates (Mohd‐Assaad, McDonald, & Croll, 2016) or virulence (Hagerty & Mundt, 2016). Using genome scans to identify genes under recent selection in pathogens will fill important gaps in our understanding of host–pathogen interactions and their evolutionary trajectory (Hall, Bento, & Ebert, 2017).

## DATA ACCESSIBILITY

The sequencing data are available at the Nucleotide Short Read Archive under BioProject Accession nos PRJNA327615 and PRJNA178194.

## AUTHOR CONTRIBUTIONS

The study was conceived by F.E.H. and D.C. The analysis was performed by F.E.H. The data were interpreted by F.E.H., B.A.M. and D.C. The manuscript was written by F.E.H. and D.C with contributions from BAM.

## References

[mec14711-bib-0001] Aguileta, G. , Refrégier, G. , Yockteng, R. , Fournier, E. , & Giraud, T. (2009). Rapidly evolving genes in pathogens: Methods for detecting positive selection and examples among fungi, bacteria, viruses and protists. Infection Genetics and Evolution, 9, 656–670. https://doi.org/10.1016/j.meegid.2009.03.010 10.1016/j.meegid.2009.03.010 19442589

[mec14711-bib-0002] Badouin, H. , Gladieux, P. , Gouzy, J. , Siguenza, S. , Aguileta, G. , Snirc, A. , … Giraud, T. (2017). Widespread selective sweeps throughout the genome of model plant pathogenic fungi and identification of effector candidates. Molecular Ecology, 26, 2041–2062. https://doi.org/10.1111/mec.13976 28012227 10.1111/mec.13976

[mec14711-bib-0003] Bahn, Y.‐S. , Xue, C. , Idnurm, A. , Rutherford, J. C. , Heitman, J. , & Cardenas, M. E. (2007). Sensing the environment: Lessons from fungi. Nature Reviews Microbiology, 5, 57–69. https://doi.org/10.1038/nrmicro1578 17170747 10.1038/nrmicro1578

[mec14711-bib-0004] Bierne, N. , Welch, J. , Loire, E. , Bonhomme, F. , & David, P. (2011). The coupling hypothesis: Why genome scans may fail to map local adaptation genes. Molecular Ecology, 20, 2044–2072. https://doi.org/10.1111/j.1365-294X.2011.05080.x 21476991 10.1111/j.1365-294X.2011.05080.x

[mec14711-bib-0005] Bolger, A. M. , Lohse, M. , & Usadel, B. (2014). trimmomatic: A flexible trimmer for Illumina sequence data. Bioinformatics, 30, 2114–2120. https://doi.org/10.1093/bioinformatics/btu170 24695404 10.1093/bioinformatics/btu170 PMC4103590

[mec14711-bib-0006] Bonhomme, M. , Boitard, S. , San Clemente, H. , Dumas, B. , Young, N. , & Jacquet, C. (2015). Genomic signature of selective sweeps illuminates adaptation of *Medicago truncatula* to root‐associated microorganisms. Molecular Biology and Evolution, 32, 2097–2110. https://doi.org/10.1093/molbev/msv092 25901015 10.1093/molbev/msv092 PMC4833077

[mec14711-bib-0007] Bradbury, P. J. , Zhang, Z. , Kroon, D. E. , Casstevens, T. M. , Ramdoss, Y. , & Buckler, E. S. (2007). tassel: Software for association mapping of complex traits in diverse samples. Bioinformatics, 23, 2633–2635. https://doi.org/10.1093/bioinformatics/btm308 17586829 10.1093/bioinformatics/btm308

[mec14711-bib-0008] Branco, S. , Bi, K. , Liao, H.‐L. , Gladieux, P. , Badouin, H. , Ellison, C. E. , … Bruns, T. D. (2017). Continental‐level population differentiation and environmental adaptation in the mushroom *Suillus brevipes* . Molecular Ecology, 26, 2063–2076. https://doi.org/10.1111/mec.13892 27761941 10.1111/mec.13892 PMC5392165

[mec14711-bib-0009] Branco, S. , Gladieux, P. , Ellison, C. E. , Kuo, A. , LaButti, K. , Lipzen, A. , … Bruns, T. D. (2015). Genetic isolation between two recently diverged populations of a symbiotic fungus. Molecular Ecology, 24, 2747–2758. https://doi.org/10.1111/mec.13132 25728665 10.1111/mec.13132

[mec14711-bib-0010] Brunner, P. C. , & McDonald, B. A. (2018). Evolutionary analyses of the avirulence effector *AvrStb6* in global populations of *Zymoseptoria tritici* identify candidate amino acids involved in recognition. Molecular Plant Pathology. https://doi.org/10.1111/mpp.12662E‐pub ahead of print. 10.1111/mpp.12662 PMC6637991 29363872

[mec14711-bib-0011] Brunner, P. C. , Stefanato, F. L. , & Mcdonald, B. A. (2008). Evolution of the CYP51 gene in *Mycosphaerella graminicola*: Evidence for intragenic recombination and selective replacement. Molecular Plant Pathology, 9, 305–316. https://doi.org/10.1111/j.1364-3703.2007.00464.x 18705872 10.1111/j.1364-3703.2007.00464.x PMC6710900

[mec14711-bib-0012] Brunner, P. C. , Stefansson, T. S. , Fountaine, J. , Richina, V. , & McDonald, B. A. (2015). A global analysis of CYP51 diversity and azole sensitivity in *Rhynchosporium commune* . Phytopathology, 106, 355–361. 10.1094/PHYTO-07-15-0158-R 26623995

[mec14711-bib-0013] Brunner, P. C. , Torriani, S. F. F. , Croll, D. , Stukenbrock, E. H. , & McDonald, B. A. (2013). Coevolution and life cycle specialization of plant cell wall degrading enzymes in a hemibiotrophic pathogen. Molecular Biology and Evolution, 30, 1337–1347. https://doi.org/10.1093/molbev/mst041 23515261 10.1093/molbev/mst041 PMC3649673

[mec14711-bib-0014] Butler, M. J. , & Day, A. W. (1998). Fungal melanins: A review. Canadian Journal of Microbiology, 44, 1115–1136. https://doi.org/10.1139/w98-119

[mec14711-bib-0015] Chen, L.‐Q. , Hou, B.‐H. , Lalonde, S. , Takanaga, H. , Hartung, M. L. , Qu, X.‐Q. , … Frommer, W. B. (2010). Sugar transporters for intercellular exchange and nutrition of pathogens. Nature, 468, 527–532. https://doi.org/10.1038/nature09606 21107422 10.1038/nature09606 PMC3000469

[mec14711-bib-0016] Cingolani, P. , Patel, V. M. , Coon, M. , Nguyen, T. , Land, S. J. , Ruden, D. M. , … Lu, X. (2012). Using *Drosophila melanogaster* as a model for genotoxic chemical mutational studies with a new program, SnpSift. Frontiers in Genetics, 3, 35. 22435069 10.3389/fgene.2012.00035 PMC3304048

[mec14711-bib-0017] Cingolani, P. , Platts, A. , Wang, L. L. , Coon, M. , Nguyen, T. , Wang, L. , … Ruden, D. M. (2012). A program for annotating and predicting the effects of single nucleotide polymorphisms, SnpEff: SNPs in the genome of *Drosophila melanogaster* strain w1118; iso‐2; iso‐3. Fly (Austin), 6, 80–92. https://doi.org/10.4161/fly.19695 22728672 10.4161/fly.19695 PMC3679285

[mec14711-bib-0018] Cools, H. J. , & Fraaije, B. A. (2013). Update on mechanisms of azole resistance in *Mycosphaerella graminicola* and implications for future control. Pest Management Science, 69, 150–155. https://doi.org/10.1002/ps.3348 22730104 10.1002/ps.3348

[mec14711-bib-0019] Coop, G. , Witonsky, D. , Rienzo, A. D. , & Pritchard, J. K. (2010). Using environmental correlations to identify loci underlying local adaptation. Genetics, 185, 1411–1423. https://doi.org/10.1534/genetics.110.114819 20516501 10.1534/genetics.110.114819 PMC2927766

[mec14711-bib-0020] Cowger, C. , Hoffer, M. E. , & Mundt, C. C. (2000). Specific adaptation by *Mycosphaerella graminicola* to a resistant wheat cultivar. Plant Pathology, 49, 445–451. https://doi.org/10.1046/j.1365-3059.2000.00472.x

[mec14711-bib-0021] Crisci, J. L. , Poh, Y.‐P. , Mahajan, S. , & Jensen, J. D. (2013). The impact of equilibrium assumptions on tests of selection. Frontiers in Genetics, 4, 235. 24273554 10.3389/fgene.2013.00235 PMC3822286

[mec14711-bib-0022] Croll, D. , Lendenmann, M. H. , Stewart, E. , & McDonald, B. A. (2015). The impact of recombination hotspots on genome evolution of a fungal plant pathogen. Genetics, 201, 1213–1228. https://doi.org/10.1534/genetics.115.180968 26392286 10.1534/genetics.115.180968 PMC4649646

[mec14711-bib-0023] Croll, D. , & McDonald, B. A. (2016). The genetic basis of local adaptation for pathogenic fungi in agricultural ecosystems. Molecular Ecology, 7, 2027–2040. 10.1111/mec.13870 27696587

[mec14711-bib-0024] Croll, D. , Zala, M. , & McDonald, B. A. (2013). Breakage‐fusion‐bridge cycles and large insertions contribute to the rapid evolution of accessory chromosomes in a fungal pathogen. PLoS Genetics, 9, e1003567 https://doi.org/10.1371/journal.pgen.1003567 23785303 10.1371/journal.pgen.1003567 PMC3681731

[mec14711-bib-0025] Dai, Y. , Jia, Y. , Correll, J. , Wang, X. , & Wang, Y. (2010). Diversification and evolution of the avirulence gene AVR‐Pita1 in field isolates of *Magnaporthe oryzae* . Fungal Genetics Biology, 47, 973–980. https://doi.org/10.1016/j.fgb.2010.08.003 20719251 10.1016/j.fgb.2010.08.003

[mec14711-bib-0026] Danecek, P. , Auton, A. , Abecasis, G. , Albers, C. A. , Banks, E. , DePristo, M. A. , … Durbin, R. (2011). The variant call format and vcftools . Bioinformatics, 27, 2156–2158. https://doi.org/10.1093/bioinformatics/btr330 21653522 10.1093/bioinformatics/btr330 PMC3137218

[mec14711-bib-0027] de Jonge, R. , Bolton, M. D. , & Thomma, B. P. (2011). How filamentous pathogens co‐opt plants: The ins and outs of fungal effectors. Current Opinion in Plant Biology, 14, 400–406. https://doi.org/10.1016/j.pbi.2011.03.005 21454120 10.1016/j.pbi.2011.03.005

[mec14711-bib-0201] de Jonge, R. , van Esse, H. P. , Maruthachalam, K. , Bolton, M. D. , Santhanam, P. , Saber, M. K. , … Thomma, B. P. (2012). Tomato immune receptor Ve1 recognizes effector of multiple fungal pathogens uncovered by genome and RNA sequencing. Proceedings of the National Academy of Sciences, 109, 5110–5115. https://doi.org/10.1073/pnas.1119623109 10.1073/pnas.1119623109 PMC3323992 22416119

[mec14711-bib-0028] Del Sorbo, G. , Schoonbeek, H. , & De Waard, M. A. (2000). Fungal transporters involved in efflux of natural toxic compounds and fungicides. Fungal Genetics and Biology, 30, 1–15. https://doi.org/10.1006/fgbi.2000.1206 10955904 10.1006/fgbi.2000.1206

[mec14711-bib-0029] Delmas, C. E. L. , Dussert, Y. , Delière, L. , Couture, C. , Mazet, I. D. , Richart Cervera, S. , & Delmotte, F. (2017). Soft selective sweeps in fungicide resistance evolution: Recurrent mutations without fitness costs in grapevine downy mildew. Molecular Ecology, 26, 1936–1951. https://doi.org/10.1111/mec.14006 28063192 10.1111/mec.14006

[mec14711-bib-0030] DePristo, M. A. , Banks, E. , Poplin, R. , Garimella, K. V. , Maguire, J. R. , Hartl, C. , … Daly, M. J. (2011). A framework for variation discovery and genotyping using next‐generation DNA sequencing data. Nature Genetics, 43, 491–498. https://doi.org/10.1038/ng.806 21478889 10.1038/ng.806 PMC3083463

[mec14711-bib-0031] Ellison, C. E. , Hall, C. , Kowbel, D. , Welch, J. , Brem, R. B. , Glass, N. L. , & Taylor, J. W. (2011). Population genomics and local adaptation in wild isolates of a model microbial eukaryote. Proceedings of the National Academy of Sciences, 108, 2831–2836. https://doi.org/10.1073/pnas.1014971108 10.1073/pnas.1014971108 PMC3041088 21282627

[mec14711-bib-0032] Estep, L. K. , Torriani, S. F. F. , Zala, M. , Anderson, N. P. , Flowers, M. D. , McDonald, B. A. , … Brunner, P. C. (2015). Emergence and early evolution of fungicide resistance in North American populations of *Zymoseptoria tritici* . Plant Pathology, 64, 961–971. https://doi.org/10.1111/ppa.12314

[mec14711-bib-0033] Evans, L. M. , Slavov, G. T. , Rodgers‐Melnick, E. , Martin, J. , Ranjan, P. , Muchero, W. , … DiFazio, S. P. (2014). Population genomics of *Populus trichocarpa* identifies signatures of selection and adaptive trait associations. Nature Genetics, 46, 1089–1096. https://doi.org/10.1038/ng.3075 25151358 10.1038/ng.3075

[mec14711-bib-0034] Eyal, Z. (1999). The *Septoria tritici* and *Stagonospora nodorum* blotch diseases of wheat. European Journal of Plant Pathology, 105, 629–641. https://doi.org/10.1023/A:1008716812259

[mec14711-bib-0035] Falcon, S. , & Gentleman, R. (2007). Using GOstats to test gene lists for GO term association. Bioinformatics, 23, 257–258. https://doi.org/10.1093/bioinformatics/btl567 17098774 10.1093/bioinformatics/btl567

[mec14711-bib-0036] Flicek, P. , Amode, M. R. , Barrell, D. , Beal, K. , Billis, K. , Brent, S. , … Searle, S. M. (2014). Ensembl 2014. Nucleic Acids Research, 42, D749–D755. https://doi.org/10.1093/nar/gkt1196 24316576 10.1093/nar/gkt1196 PMC3964975

[mec14711-bib-0037] Fones, H. , & Gurr, S. (2015). The impact of *Septoria tritici* blotch disease on wheat: An EU perspective. Fungal Genetics and Biology, 79, 3–7. https://doi.org/10.1016/j.fgb.2015.04.004 26092782 10.1016/j.fgb.2015.04.004 PMC4502551

[mec14711-bib-0038] Garrison, E. , & Marth, G. (2012). Haplotype‐based variant detection from short‐read sequencing. ArXiv12073907 Q‐BioGN.

[mec14711-bib-0039] Gautier, M. (2015). Genome‐wide scan for adaptive divergence and association with population‐specific covariates. Genetics, 201, 1555–1579. https://doi.org/10.1534/genetics.115.181453 26482796 10.1534/genetics.115.181453 PMC4676524

[mec14711-bib-0040] Gautier, M. , & Vitalis, R. (2012). rehh: An R package to detect footprints of selection in genome‐wide SNP data from haplotype structure. Bioinformatics, 28, 1176–1177. https://doi.org/10.1093/bioinformatics/bts115 22402612 10.1093/bioinformatics/bts115

[mec14711-bib-0041] Goodwin, S. B. , M'Barek, S. B. , Dhillon, B. , Wittenberg, A. H. J. , Crane, C. F. , Hane, J. K. , & Kema, G. H. (2011). Finished genome of the fungal wheat pathogen *Mycosphaerella graminicola* reveals dispensome structure, chromosome plasticity, and stealth pathogenesis. PLoS Genetics, 7, e1002070 https://doi.org/10.1371/journal.pgen.1002070 21695235 10.1371/journal.pgen.1002070 PMC3111534

[mec14711-bib-0042] Goudet, J. (2005). hierfstat, a package for r to compute and test hierarchical F‐statistics. Molecular Ecology Notes, 5, 184–186. https://doi.org/10.1111/j.1471-8286.2004.00828.x

[mec14711-bib-0043] Grandaubert, J. , Bhattacharyya, A. , & Stukenbrock, E. H. (2015). RNA‐seq‐based gene annotation and comparative genomics of four fungal grass pathogens in the genus *Zymoseptoria* identify novel orphan genes and species‐specific invasions of transposable elements. G3: Genes, Genomes, Genetics, 5, 1323–1333. https://doi.org/10.1534/g3.115.017731 25917918 10.1534/g3.115.017731 PMC4502367

[mec14711-bib-0044] Günther, T. , & Coop, G. (2013). Robust identification of local adaptation from allele frequencies. Genetics, 195, 205–220. https://doi.org/10.1534/genetics.113.152462 23821598 10.1534/genetics.113.152462 PMC3761302

[mec14711-bib-0045] Hagerty, C. H. , & Mundt, C. C. (2016). Reduced virulence of azoxystrobin‐resistant *Zymoseptoria tritici* populations in greenhouse assays. Phytopathology, 106, 884–889. https://doi.org/10.1094/PHYTO-01-16-0029-R 27249373 10.1094/PHYTO-01-16-0029-R

[mec14711-bib-0046] Hahn, M. (2014). The rising threat of fungicide resistance in plant pathogenic fungi: Botrytis as a case study. Journal of Chemical Biology, 7, 133–141. https://doi.org/10.1007/s12154-014-0113-1 25320647 10.1007/s12154-014-0113-1 PMC4182335

[mec14711-bib-0047] Hall, M. D. , Bento, G. , & Ebert, D. (2017). The evolutionary consequences of stepwise infection processes. Trends in Ecology & Evolution, 32, 612–623. https://doi.org/10.1016/j.tree.2017.05.009 28648806 10.1016/j.tree.2017.05.009

[mec14711-bib-0048] Hartmann, F. E. , & Croll, D. (2017). Distinct trajectories of massive recent gene gains and losses in populations of a microbial eukaryotic pathogen. Molecular Biology and Evolution, https://doi.org/10.1093/molbev/msx208 10.1093/molbev/msx208 PMC5850472 28981698

[mec14711-bib-0049] Hartmann, F. E. , Sánchez‐Vallet, A. , McDonald, B. A. , & Croll, D. (2017). A fungal wheat pathogen evolved host specialization by extensive chromosomal rearrangements. The ISME Journal, 11, 1189–1204. https://doi.org/10.1038/ismej.2016.196 28117833 10.1038/ismej.2016.196 PMC5437930

[mec14711-bib-0050] Hoban, S. , Kelley, J. L. , Lotterhos, K. E. , Antolin, M. F. , Bradburd, G. , Lowry, D. B. , … Whitlock, M. C. (2016). Finding the genomic basis of local adaptation: Pitfalls, practical solutions, and future directions. The American Naturalist, 188, 379–397. https://doi.org/10.1086/688018 10.1086/688018 PMC5457800 27622873

[mec14711-bib-0051] Howlett, B. J. (2006). Secondary metabolite toxins and nutrition of plant pathogenic fungi. Current Opinion in Plant Biology, 9, 371–375. https://doi.org/10.1016/j.pbi.2006.05.004 16713733 10.1016/j.pbi.2006.05.004

[mec14711-bib-0053] Jakobsson, M. , & Rosenberg, N. A. (2007). clumpp: A cluster matching and permutation program for dealing with label switching and multimodality in analysis of population structure. Bioinformatics, 23, 1801–1806. https://doi.org/10.1093/bioinformatics/btm233 17485429 10.1093/bioinformatics/btm233

[mec14711-bib-0054] Jones, P. , Binns, D. , Chang, H.‐Y. , Fraser, M. , Li, W. , McAnulla, C. , … Hunter, S. (2014). interproscan 5: Genome‐scale protein function classification. Bioinformatics, 30, 1236–1240. https://doi.org/10.1093/bioinformatics/btu031 24451626 10.1093/bioinformatics/btu031 PMC3998142

[mec14711-bib-0055] Jones, J. D. G. , & Dangl, J. L. (2006). The plant immune system. Nature, 444, 323–329. https://doi.org/10.1038/nature05286 17108957 10.1038/nature05286

[mec14711-bib-0056] Käll, L. , Krogh, A. , & Sonnhammer, E. L. L. (2007). Advantages of combined transmembrane topology and signal peptide prediction—the phobius web server. Nucleic Acids Research, 35, W429–W432. https://doi.org/10.1093/nar/gkm256 17483518 10.1093/nar/gkm256 PMC1933244

[mec14711-bib-0057] Krogh, A. , Larsson, B. , von Heijne, G. , & Sonnhammer, E. L. L. (2001). Predicting transmembrane protein topology with a hidden Markov model: Application to complete genomes. Journal of Molecular Biology, 305, 567–580. https://doi.org/10.1006/jmbi.2000.4315 11152613 10.1006/jmbi.2000.4315

[mec14711-bib-0058] Langmead, B. , Trapnell, C. , Pop, M. , & Salzberg, S. L. (2009). Ultrafast and memory‐efficient alignment of short DNA sequences to the human genome. Genome Biology, 10, R25 https://doi.org/10.1186/gb-2009-10-3-r25 19261174 10.1186/gb-2009-10-3-r25 PMC2690996

[mec14711-bib-0059] Lendenmann, M. H. , Croll, D. , & McDonald, B. A. (2015). QTL mapping of fungicide sensitivity reveals novel genes and pleiotropy with melanization in the pathogen *Zymoseptoria tritici* . Fungal Genetics and Biology, 80, 53–67. https://doi.org/10.1016/j.fgb.2015.05.001 25979163 10.1016/j.fgb.2015.05.001

[mec14711-bib-0060] Lendenmann, M. H. , Croll, D. , Palma‐Guerrero, J. , Stewart, E. L. , & McDonald, B. A. (2016). QTL mapping of temperature sensitivity reveals candidate genes for thermal adaptation and growth morphology in the plant pathogenic fungus *Zymoseptoria tritici* . Heredity, 116, 384–394. https://doi.org/10.1038/hdy.2015.111 26758189 10.1038/hdy.2015.111 PMC4806695

[mec14711-bib-0061] Lendenmann, M. H. , Croll, D. , Stewart, E. L. , & McDonald, B. A. (2014). Quantitative trait locus mapping of melanization in the plant pathogenic fungus *Zymoseptoria tritici* . G3 (Bethesda Md), 4, 2519–2533. https://doi.org/10.1534/g3.114.015289 10.1534/g3.114.015289 PMC4267946 25360032

[mec14711-bib-0062] Mboup, M. , Bahri, B. , Leconte, M. , De Vallavieille‐Pope, C. , Kaltz, O. , & Enjalbert, J. (2012). Genetic structure and local adaptation of European wheat yellow rust populations: The role of temperature‐specific adaptation. Evolutionary Applications, 5, 341–352. https://doi.org/10.1111/j.1752-4571.2011.00228.x 25568055 10.1111/j.1752-4571.2011.00228.x PMC3353355

[mec14711-bib-0063] McDonald, M. C. , Oliver, R. P. , Friesen, T. L. , Brunner, P. C. , & McDonald, B. A. (2013). Global diversity and distribution of three necrotrophic effectors in *Phaeosphaeria nodorum* and related species. New Phytologist, 199, 241–251. https://doi.org/10.1111/nph.12257 23550706 10.1111/nph.12257

[mec14711-bib-0064] McDonald, B. A. , & Stukenbrock, E. H. (2016). Rapid emergence of pathogens in agro‐ecosystems: Global threats to agricultural sustainability and food security. Philosophical Transactions of the Royal Society B, 371, 20160026 https://doi.org/10.1098/rstb.2016.0026 10.1098/rstb.2016.0026 PMC5095548 28080995

[mec14711-bib-0065] McKenna, A. , Hanna, M. , Banks, E. , Sivachenko, A. , Cibulskis, K. , Kernytsky, A. , … DePristo, M. A. (2010). The Genome Analysis Toolkit: A MapReduce framework for analyzing next‐generation DNA sequencing data. Genome Research, 20, 1297–1303. https://doi.org/10.1101/gr.107524.110 20644199 10.1101/gr.107524.110 PMC2928508

[mec14711-bib-0066] Messer, P. W. , & Petrov, D. A. (2013). Population genomics of rapid adaptation by soft selective sweeps. Trends in Ecology & Evolution, 28, 659–669. https://doi.org/10.1016/j.tree.2013.08.003 24075201 10.1016/j.tree.2013.08.003 PMC3834262

[mec14711-bib-0067] Mirzadi Gohari, A. , Ware, S. B. , Wittenberg, A. H. J. , Mehrabi, R. , Ben M'Barek, S. , Verstappen, E. C. P. , … Kema, G. H. (2015). Effector discovery in the fungal wheat pathogen *Zymoseptoria tritici* . Molecular Plant Pathology, 16, 931–945. https://doi.org/10.1111/mpp.12251 25727413 10.1111/mpp.12251 PMC6638447

[mec14711-bib-0068] Mohd‐Assaad, N. , McDonald, B. A. , & Croll, D. (2016). Multilocus resistance evolution to azole fungicides in fungal plant pathogen populations. Molecular Ecology, 25, 6124–6142. https://doi.org/10.1111/mec.13916 27859799 10.1111/mec.13916

[mec14711-bib-0069] Nei, M. (1973). Analysis of gene diversity in subdivided populations. Proceedings of the National Academy of Sciences, 70, 3321–3323. https://doi.org/10.1073/pnas.70.12.3321 10.1073/pnas.70.12.3321 PMC427228 4519626

[mec14711-bib-0070] Nielsen, R. (2005). Molecular signatures of natural selection. Annual Review of Genetics, 39, 197–218. https://doi.org/10.1146/annurev.genet.39.073003.112420 10.1146/annurev.genet.39.073003.112420 16285858

[mec14711-bib-0071] O'Driscoll, A. , Kildea, S. , Doohan, F. , Spink, J. , & Mullins, E. (2014). The wheat–Septoria conflict: A new front opening up? Trends in Plant Science, 19, 602–610. https://doi.org/10.1016/j.tplants.2014.04.011 24957882 10.1016/j.tplants.2014.04.011

[mec14711-bib-0072] Ohm, R. A. , Feau, N. , Henrissat, B. , Schoch, C. L. , Horwitz, B. A. , Barry, K. W. , … Grigoriev, I. V. (2012). Diverse lifestyles and strategies of plant pathogenesis encoded in the genomes of eighteen Dothideomycetes fungi. PLoS Pathogens, 8, e1003037 https://doi.org/10.1371/journal.ppat.1003037 23236275 10.1371/journal.ppat.1003037 PMC3516569

[mec14711-bib-0073] Palma‐Guerrero, J. , Ma, X. , Torriani, S. F. F. , Zala, M. , Francisco, C. S. , Hartmann, F. E. , … McDonald, B. A. (2017). Comparative transcriptome analyses in *Zymoseptoria tritici* reveal significant differences in gene expression among strains during plant infection. Molecular Plant‐Microbe Interactions, 30, 231–244. https://doi.org/10.1094/MPMI-07-16-0146-R 28121239 10.1094/MPMI-07-16-0146-R

[mec14711-bib-0074] Park, D. J. , Lukens, A. K. , Neafsey, D. E. , Schaffner, S. F. , Chang, H.‐H. , Valim, C. , … Volkman, S. K. (2012). Sequence‐based association and selection scans identify drug resistance loci in the *Plasmodium falciparum* malaria parasite. Proceedings of the National Academy of Sciences, 109, 13052–13057. https://doi.org/10.1073/pnas.1210585109 10.1073/pnas.1210585109 PMC3420184 22826220

[mec14711-bib-0075] Pavlidis, P. , Živković, D. , Stamatakis, A. , & Alachiotis, N. (2013). sweed: Likelihood‐based detection of selective sweeps in thousands of genomes. Molecular Biology and Evolution, 9, 2224–2234. https://doi.org/10.1093/molbev/mst112 10.1093/molbev/mst112 PMC3748355 23777627

[mec14711-bib-0203] Pavlidis, P. , & Alachiotis, N. (2017). A survey of methods and tools to detect recent and strong positive selection. Journal of Biological Research – Thessaloniki, 24, 7 https://doi.org/10.1186/s40709-017-0064-0 10.1186/s40709-017-0064-0 PMC5385031 28405579

[mec14711-bib-0076] Pereira, D. A. , McDonald, B. A. , & Brunner, P. C. (2017). Mutations in the CYP51 gene reduce DMI sensitivity in Parastagonospora nodorum populations in Europe and China. Pest Management Science, 7, 1503–1510. https://doi.org/10.1002/ps.4486 10.1002/ps.4486 27860315

[mec14711-bib-0077] Petersen, T. N. , Brunak, S. , von Heijne, G. , & Nielsen, H. (2011). signalp 4.0: Discriminating signal peptides from transmembrane regions. Nature Methods, 8, 785–786. https://doi.org/10.1038/nmeth.1701 21959131 10.1038/nmeth.1701

[mec14711-bib-0078] Pfeifer, B. , Wittelsbürger, U. , Ramos‐Onsins, S. E. , & Lercher, M. J. (2014). popgenome: an efficient Swiss army knife for population genomic analyses in R. Molecular Biology and Evolution, 31, 1929–1936. https://doi.org/10.1093/molbev/msu136 24739305 10.1093/molbev/msu136 PMC4069620

[mec14711-bib-0079] Poppe, S. , Dorsheimer, L. , Happel, P. , & Stukenbrock, E. H. (2015). Rapidly evolving genes are key players in host specialization and virulence of the fungal wheat pathogen *Zymoseptoria tritici* (*Mycosphaerella graminicola*). PLOS Pathogens, 11, e1005055 https://doi.org/10.1371/journal.ppat.1005055 26225424 10.1371/journal.ppat.1005055 PMC4520584

[mec14711-bib-0080] Presti, L. L. , Lanver, D. , Schweizer, G. , Tanaka, S. , Liang, L. , Tollot, M. , … Kahmann, R. (2015). Fungal effectors and plant susceptibility. Annual Review of Plant Biology, 66, 513–545. https://doi.org/10.1146/annurev-arplant-043014-114623 10.1146/annurev-arplant-043014-114623 25923844

[mec14711-bib-0081] Pritchard, J. K. , Stephens, M. , & Donnelly, P. (2000). Inference of population structure using multilocus genotype data. Genetics, 155, 945–959. 10835412 10.1093/genetics/155.2.945 PMC1461096

[mec14711-bib-0205] Qanbari, S. , Pausch, H. , Jansen, S. , Somel, M. , Strom, T. M. , Fries, R. , … Simianer, H. (2014). Classic selective sweeps revealed by massive sequencing in cattle. PLOS Genetics, 10, e1004148 https://doi.org/10.1371/journal.pgen.1004148 24586189 10.1371/journal.pgen.1004148 PMC3937232

[mec14711-bib-0082] Rep, M. (2005). Small proteins of plant‐pathogenic fungi secreted during host colonization. FEMS Microbiology Letters, 253, 19–27. https://doi.org/10.1016/j.femsle.2005.09.014 16216445 10.1016/j.femsle.2005.09.014

[mec14711-bib-0083] Rosenberg, N. A. (2004). distruct: A program for the graphical display of population structure. Molecular Ecology Notes, 4, 137–138.

[mec14711-bib-0084] Rudd, J. J. , Kanyuka, K. , Hassani‐Pak, K. , Derbyshire, M. , Andongabo, A. , Devonshire, J. , … Courbot, M. (2015). Transcriptome and metabolite profiling of the infection cycle of *Zymoseptoria tritici* on wheat reveals a biphasic interaction with plant immunity involving differential pathogen chromosomal contributions and a variation on the hemibiotrophic lifestyle definition. Plant Physiology, 167, 1158–1185. https://doi.org/10.1104/pp.114.255927 25596183 10.1104/pp.114.255927 PMC4348787

[mec14711-bib-0085] Sabeti, P. C. , Varilly, P. , Fry, B. , Lohmueller, J. , Hostetter, E. , Cotsapas, C. , … Stewart, J. (2007). Genome‐wide detection and characterization of positive selection in human populations. Nature, 449, 913–918. https://doi.org/10.1038/nature06250 17943131 10.1038/nature06250 PMC2687721

[mec14711-bib-0086] Shin, J.‐H. , Blay, S. , Graham, J. , & McNeney, B. (2006). ldheatmap: An R function for graphical display of pairwise linkage disequilibria between single nucleotide polymorphisms. Journal of Statistical Software, 16, 1–10.

[mec14711-bib-0087] Singh, R. P. , Hodson, D. P. , Huerta‐Espino, J. , Jin, Y. , Bhavani, S. , Njau, P. , … Govindan, V. (2011). The emergence of Ug99 races of the stem rust fungus is a threat to world wheat production. Annual Review of Phytopathology, 49, 465–481. https://doi.org/10.1146/annurev-phyto-072910-095423 10.1146/annurev-phyto-072910-095423 21568701

[mec14711-bib-0088] Stefansson, T. S. , McDonald, B. A. , & Willi, Y. (2013). Local adaptation and evolutionary potential along a temperature gradient in the fungal pathogen *Rhynchosporium commune* . Evolutionary Applications, 6, 524–534. https://doi.org/10.1111/eva.12039 23745143 10.1111/eva.12039 PMC3673479

[mec14711-bib-0089] Stewart, E. L. , Croll, D. , Lendenmann, M. H. , Sanchez‐Vallet, A. , Hartmann, F. E. , Palma‐Guerrero, J. , & McDonald, B. A. (2018). QTL mapping reveals complex genetic architecture of quantitative virulence in the wheat pathogen *Zymoseptoria tritici* . Molecular Plant Pathology, https://doi.org/10.1111/mpp.12515 10.1111/mpp.12515 PMC6638037 27868326

[mec14711-bib-0090] Stukenbrock, E. H. , Banke, S. , Javan‐Nikkhah, M. , & McDonald, B. A. (2007). Origin and domestication of the fungal wheat pathogen *Mycosphaerella graminicola* via sympatric speciation. Molecular Biology and Evolution, 24, 398–411. 17095534 10.1093/molbev/msl169

[mec14711-bib-0091] Stukenbrock, E. H. , Bataillon, T. , Dutheil, J. Y. , Hansen, T. T. , Li, R. , Zala, M. , … Schierup, M. H. (2011). The making of a new pathogen: Insights from comparative population genomics of the domesticated wheat pathogen *Mycosphaerella graminicola* and its wild sister species. Genome Research, 21, 2157–2166. https://doi.org/10.1101/gr.118851.110 21994252 10.1101/gr.118851.110 PMC3227104

[mec14711-bib-0092] Stukenbrock, E. H. , Christiansen, F. B. , Hansen, T. T. , Dutheil, J. Y. , & Schierup, M. H. (2012). Fusion of two divergent fungal individuals led to the recent emergence of a unique widespread pathogen species. Proceedings of the National Academy of Sciences, 109, 10954–10959. https://doi.org/10.1073/pnas.1201403109 10.1073/pnas.1201403109 PMC3390827 22711811

[mec14711-bib-0093] Stukenbrock, E. H. , & McDonald, B. A. (2008). The origins of plant pathogens in agro‐ecosystems. Annual Review of Phytopathology, 46,75–100. https://doi.org/10.1146/annurev.phyto.010708.154114 10.1146/annurev.phyto.010708.154114 18680424

[mec14711-bib-0094] Stukenbrock, E. H. , & McDonald, B. A. (2009). Population genetics of fungal and oomycete effectors involved in gene‐for‐gene interactions. Molecular Plant‐Microbe Interactions, 22, 371–380. https://doi.org/10.1094/MPMI-22-4-0371 19271952 10.1094/MPMI-22-4-0371

[mec14711-bib-0095] Tajima, F. (1989). Statistical method for testing the neutral mutation hypothesis by DNA polymorphism. Genetics, 123, 585–595. 2513255 10.1093/genetics/123.3.585 PMC1203831

[mec14711-bib-0096] Torriani, S. F. , Brunner, P. C. , McDonald, B. A. , & Sierotzki, H. (2009). QoI resistance emerged independently at least 4 times in European populations of *Mycosphaerella graminicola* . Pest Management Science, 65, 155–162. https://doi.org/10.1002/ps.1662 18833571 10.1002/ps.1662

[mec14711-bib-0097] Torriani, S. F. F. , Stukenbrock, E. H. , Brunner, P. C. , McDonald, B. A. , & Croll, D. (2011). Evidence for extensive recent intron transposition in closely related fungi. Current Biology, 21, 2017–2022. https://doi.org/10.1016/j.cub.2011.10.041 22100062 10.1016/j.cub.2011.10.041

[mec14711-bib-0098] Van de Wouw, A. P. , Cozijnsen, A. J. , Hane, J. K. , Brunner, P. C. , McDonald, B. A. , Oliver, R. P. , & Howlett, B. J. (2010). Evolution of linked avirulence effectors in *Leptosphaeria maculans* is affected by genomic environment and exposure to resistance genes in host plants. PLOS Pathogens, 6, e1001180 https://doi.org/10.1371/journal.ppat.1001180 21079787 10.1371/journal.ppat.1001180 PMC2973834

[mec14711-bib-0099] Van der Auwera, G. A. , Carneiro, M. O. , Hartl, C. , Poplin, R. , del Angel, G. , Levy‐Moonshine, A. , … DePristo, M. A. (2002). From FastQ data to high‐confidence variant calls: The genome analysis toolkit best practices pipeline. Current Protocols in Bioinformatics, 43, 11.10.1–11.10.33. 10.1002/0471250953.bi1110s43 PMC4243306 25431634

[mec14711-bib-0100] Vitti, J. J. , Grossman, S. R. , & Sabeti, P. C. (2013). Detecting natural selection in genomic data. Annual Review of Genetics, 47, 97–120. https://doi.org/10.1146/annurev-genet-111212-133526 10.1146/annurev-genet-111212-133526 24274750

[mec14711-bib-0101] Voight, B. F. , Kudaravalli, S. , Wen, X. , & Pritchard, J. K. (2006). A map of recent positive selection in the human genome. PLOS Biology, 4, e72 https://doi.org/10.1371/journal.pbio.0040072 16494531 10.1371/journal.pbio.0040072 PMC1382018

[mec14711-bib-0102] Wahl, R. , Wippel, K. , Goos, S. , Kämper, J. , & Sauer, N. (2010). A novel high‐affinity sucrose transporter is required for virulence of the plant pathogen *Ustilago maydis* . PLOS Biology, 8, e1000303. 20161717 10.1371/journal.pbio.1000303 PMC2817709

[mec14711-bib-0103] Walker, A.‐S. , Ravigne, V. , Rieux, A. , Ali, S. , Carpentier, F. , & Fournier, E. (2017). Fungal adaptation to contemporary fungicide applications: The case of *Botrytis cinerea* populations from Champagne vineyards (France). Molecular Ecology, 26, 1919–1935. https://doi.org/10.1111/mec.14072 28231406 10.1111/mec.14072

[mec14711-bib-0104] Weigel, D. , & Nordborg, M. (2015). Population genomics for understanding adaptation in wild plant species. Annual Review of Genetics, 49, 315–338. https://doi.org/10.1146/annurev-genet-120213-092110 10.1146/annurev-genet-120213-092110 26436459

[mec14711-bib-0105] Wright, S. (1951). The genetical structure of populations. Annals of Eugenics, 15, 323–354. 24540312 10.1111/j.1469-1809.1949.tb02451.x

[mec14711-bib-0106] Yang, R.‐C. (1998). Estimating hierarchical F‐Statistics. Evolution, 52, 950–956. 28565205 10.1111/j.1558-5646.1998.tb01824.x

[mec14711-bib-0107] Yang, L. , Gao, F. , Shang, L. , Zhan, J. , & McDonald, B. A. (2013). Association between virulence and triazole tolerance in the phytopathogenic fungus *Mycosphaerella graminicola* . PLoS ONE, 8, e59568 https://doi.org/10.1371/journal.pone.0059568 23555044 10.1371/journal.pone.0059568 PMC3598747

[mec14711-bib-0108] Zhan, J. , Linde, C. C. , Jürgens, T. , Merz, U. , Steinebrunner, F. , & McDonald, B. A. (2005). Variation for neutral markers is correlated with variation for quantitative traits in the plant pathogenic fungus *Mycosphaerella graminicola* . Molecular Ecology, 14, 2683–2693. https://doi.org/10.1111/j.1365-294X.2005.02638.x 16029470 10.1111/j.1365-294X.2005.02638.x

[mec14711-bib-0109] Zhan, J. , & McDonald, B. A. (2011). Thermal adaptation in the fungal pathogen *Mycosphaerella graminicola* . Molecular Ecology, 20, 1689–1701. https://doi.org/10.1111/j.1365-294X.2011.05023.x 21395890 10.1111/j.1365-294X.2011.05023.x

[mec14711-bib-0110] Zhan, J. , Stefanato, F. L. , & McDonald, B. A. (2006). Selection for increased cyproconazole tolerance in *Mycosphaerella graminicola* through local adaptation and in response to host resistance. Molecular Plant Pathology, 7, 259–268. https://doi.org/10.1111/j.1364-3703.2006.00336.x 20507445 10.1111/j.1364-3703.2006.00336.x

[mec14711-bib-0111] Zhong, Z. , Marcel, T. C. , Hartmann, F. E. , Ma, X. , Plissonneau, C. , Zala, M. , … Palma‐Guerrero, J. (2017). A small secreted protein in *Zymoseptoria tritici* is responsible for avirulence on wheat cultivars carrying the Stb6 resistance gene. New Phytologist, 214, 619–631. https://doi.org/10.1111/nph.14434 28164301 10.1111/nph.14434

