# Supplementary material for: Genome‐wide evidence for divergent selection between populations of a major agricultural pathogen
Source: Mol Ecol. 2018 May 23;27(12):2725–41. doi: 10.1111/mec.14711 (PMC6032900; doi:10.1111/mec.14711)
Supplement: Supplementary file 1 [file MEC-27-2725-s001.pdf]

MOLECULAR ECOLOGY

Supplemental Information for:

Genome-wide evidence for divergent selection between populations of a major agricultural pathogen

Fanny E. Hartmann[1,2], Bruce A. McDonald[1], Daniel Croll[3]

[1] Plant Pathology, Institute of Integrative Biology, ETH Zurich, 8092 Zurich, Switzerland

[2] Ecologie Systématique Evolution, Univ. Paris-Sud, AgroParisTech, CNRS, Université Paris-Saclay, 91400 Orsay, France

[3] Laboratory of Evolutionary Genetics, Institute of Biology, University of Neuchâtel, 2000 Neuchâtel, Switzerland

Summary:

Supplementary Figures

Fig. S1: Comparison of the numbers and the quality scores of SNPs called with the Genome Analysis Toolkit (GATK) HaplotypeCaller and Freebayes.

Fig. S2: Principal component analysis (PCA) based on genome-wide SNPs among the 123 *Zymoseptoria tritici* isolates.

Fig. S3: Genome-wide distribution of per gene nucleotide diversity per site (π) and per gene Tajima's D statistics in the four allopatric *Zymoseptoria tritici* populations.

Fig. S4: Distribution of per gene nucleotide diversity per site (π) and per gene Tajima's D statistics according to the gene location within a selective sweep region identified in the composite likelihood ratio (CLR) test and the extended haplotype homozygosity (EHH) test or outside a detected selective sweep region.

Fig. S5: Distribution of XtX statistics values among the four allopatric *Zymoseptoria tritici* populations obtained (A) for randomly selected genome-wide distributed synonymous SNPs and (B) computed using a simulated pseudo observed dataset.

Fig. S6: Distribution of XtX statistics values among the two Oregon sympatric *Zymoseptoria tritici* populations.

MOLECULAR ECOLOGY

Fig. S7: Cross-population extended haplotype homozygosity (XP-EHH) scan among the two Oregon sympatric *Zymoseptoria tritici* populations.

Supplementary Tables

Table S1: Accession numbers and statistics for the whole-genome sequencing data of 123 *Zymoseptoria tritici* isolates used in this study.

Table S2: Genetic diversity statistics of the four allopatric *Zymoseptoria tritici* populations.

Table S3: Summary statistics of the selective sweep regions identified in the *Zymoseptoria tritici* population sampled in Australia.

Table S4: Summary statistics of the selective sweep regions identified in the *Zymoseptoria tritici* population sampled in Switzerland.

Table S5: Summary statistics of the selective sweep regions identified in the *Zymoseptoria tritici* population sampled in Israel.

Table S6: Summary statistics of the selective sweep regions identified in the *Zymoseptoria tritici* population sampled in Oregon.

Table S7: List of the outlier SNPs detected using the population divergence scan among the four allopatric *Zymoseptoria tritici* populations.

Table S8: List of the outlier SNPs detected in the cross-population extended haplotype homozygosity (XP-EHH) scan among the two Oregon *Zymoseptoria tritici* sympatric populations.

Table S9: Significantly over-represented gene ontology terms linked to biological processes in selective sweep regions of all four allopatric *Zymoseptoria tritici* populations.

Table S10: List of genes found in selective sweep regions in the four allopatric *Zymoseptoria tritici* populations.

Table S11: List of the genes found in regions highly differentiated among the four allopatric *Zymoseptoria tritici* populations.

Table S12: List of the genes found in regions highly differentiated among the two Oregon sympatric *Zymoseptoria tritici* populations.

MOLECULAR ECOLOGY

Supplementary Notes

Note S1: Population structure of the 123 *Zymoseptoria tritici* isolates.

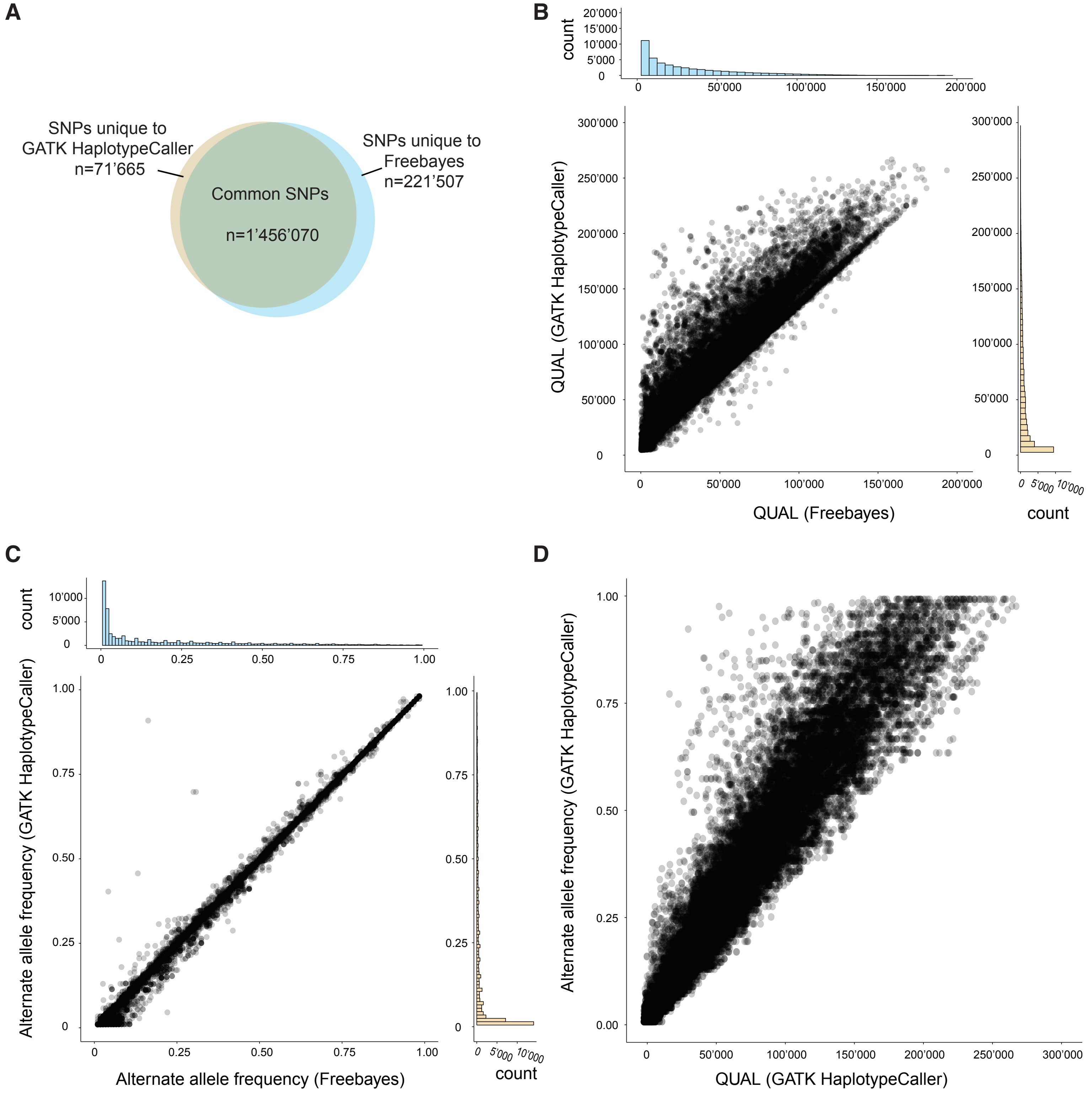

Fig. S1: Comparison of the numbers and the quality scores of SNPs called with the Genome Analysis Toolkit (GATK) HaplotypeCaller and Freebayes. A. Venn diagram of the number of shared SNPs and unique SNPs called by GATK HaplotypeCaller and Freebayes, respectively. B. Correlation between QUAL parameters of the SNPs jointly called with GATK HaplotypeCaller and Freebayes (Pearson's correlation coefficient, r = 0.958; p-value < 2.2e-16). C. Correlation between alternate allele frequencies of the SNPs jointly called with GATK HaplotypeCaller and Freebayes (Pearson's correlation coefficient, r = 0.999; p-value < 2.2e-16). D. Correlation of alternate allele frequencies and QUAL parameters of the final set of SNPs kept for further analyses. Statistics obtained from the GATK HaplotypeCaller were plotted (Pearson's correlation coefficient, r = 0.968; p-value < 2.2e-16). A positive correlation was expected as QUAL quantifies the overall confidence that there exists an alternative allele at a certain position. Therefore, overall number of reads matching the alternative allele directly contributes to higher QUAL. The panels B , C and D show only SNPs located on chromosome 13 (n=69,866) for simplicity.

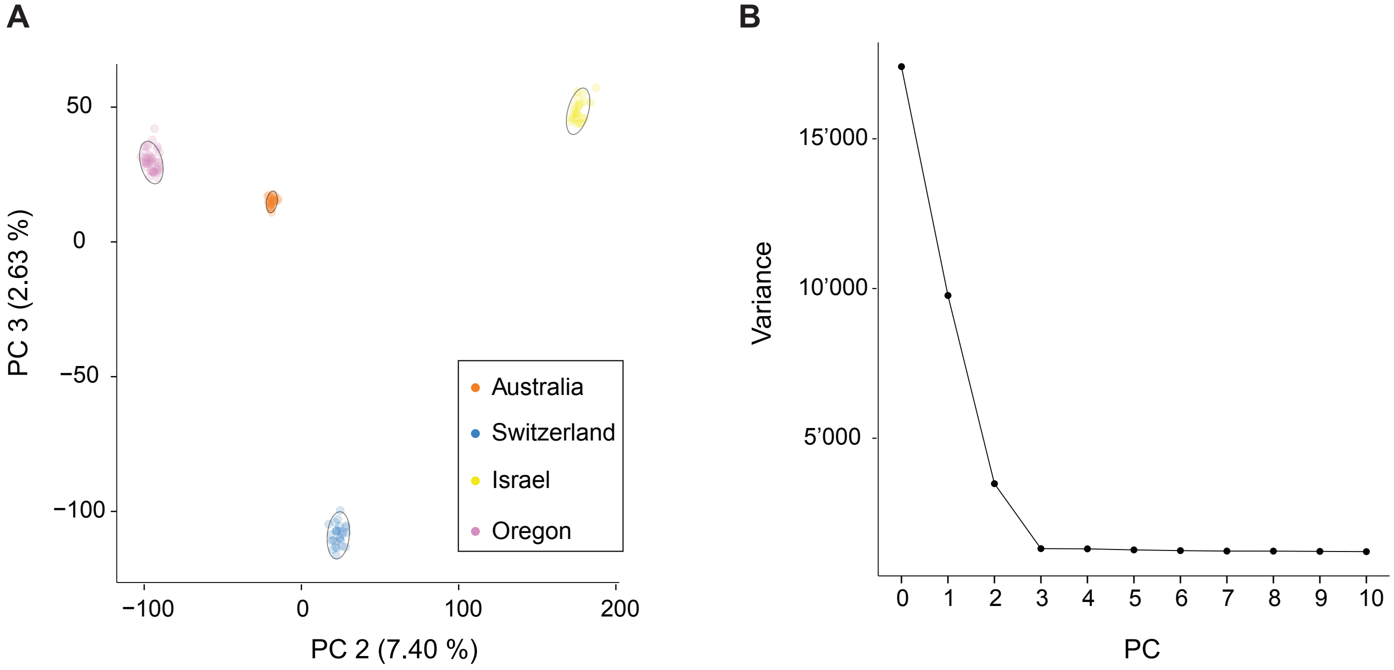

Fig. S2: Principal component analysis (PCA) based on genome-wide SNPs among the 123 *Zymoseptoria tritici* isolates. A. PCA showing the second and third principal components. The percentage of variance explained by each component is shown in parentheses. B. Screen plot showing the genetic variance explained by the first 10 PCs.

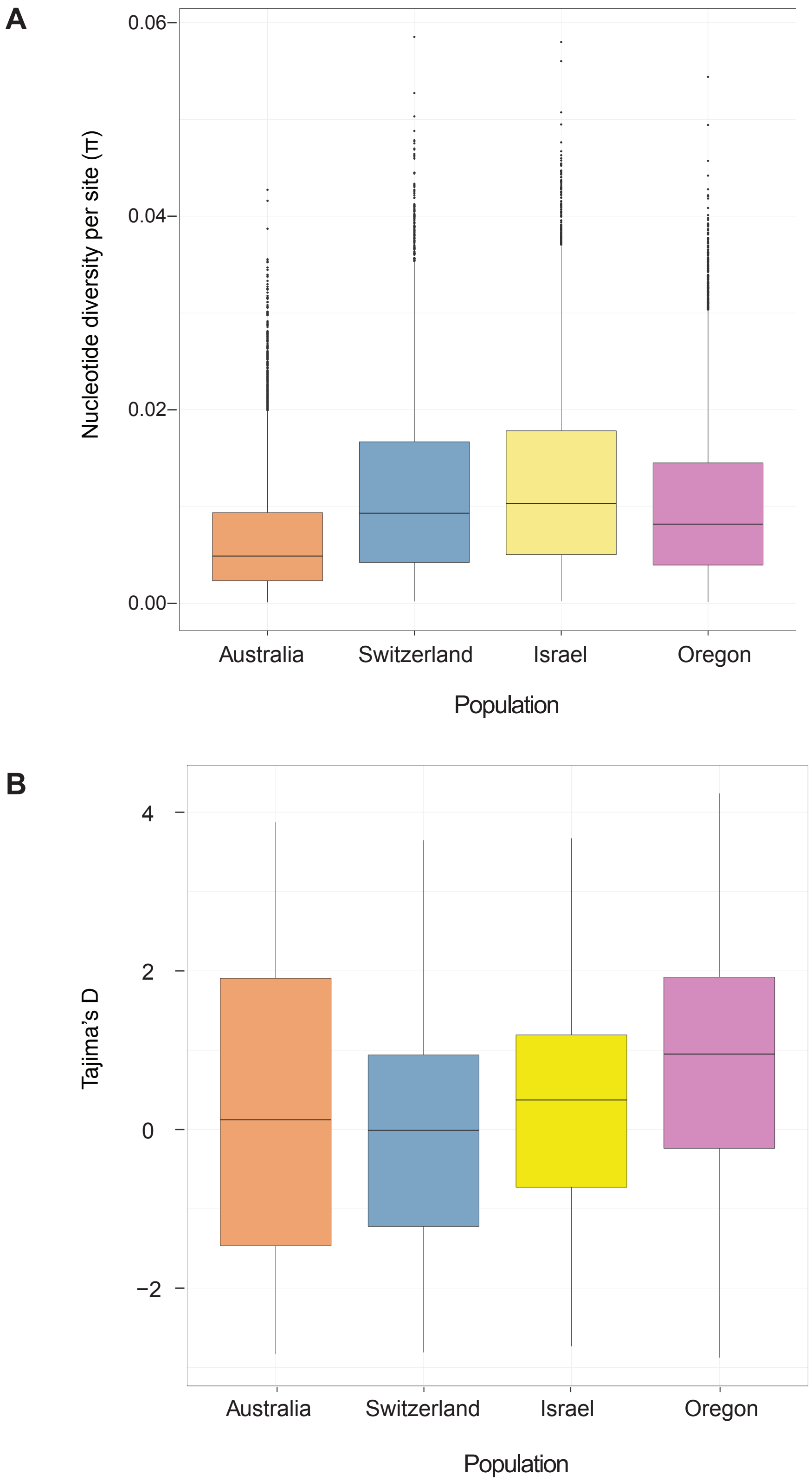

Fig. S3: Genome-wide distribution of per gene nucleotide diversity per site (π) and per gene Tajima's D statistics in the four allopatric *Zymoseptoria tritici* populations.

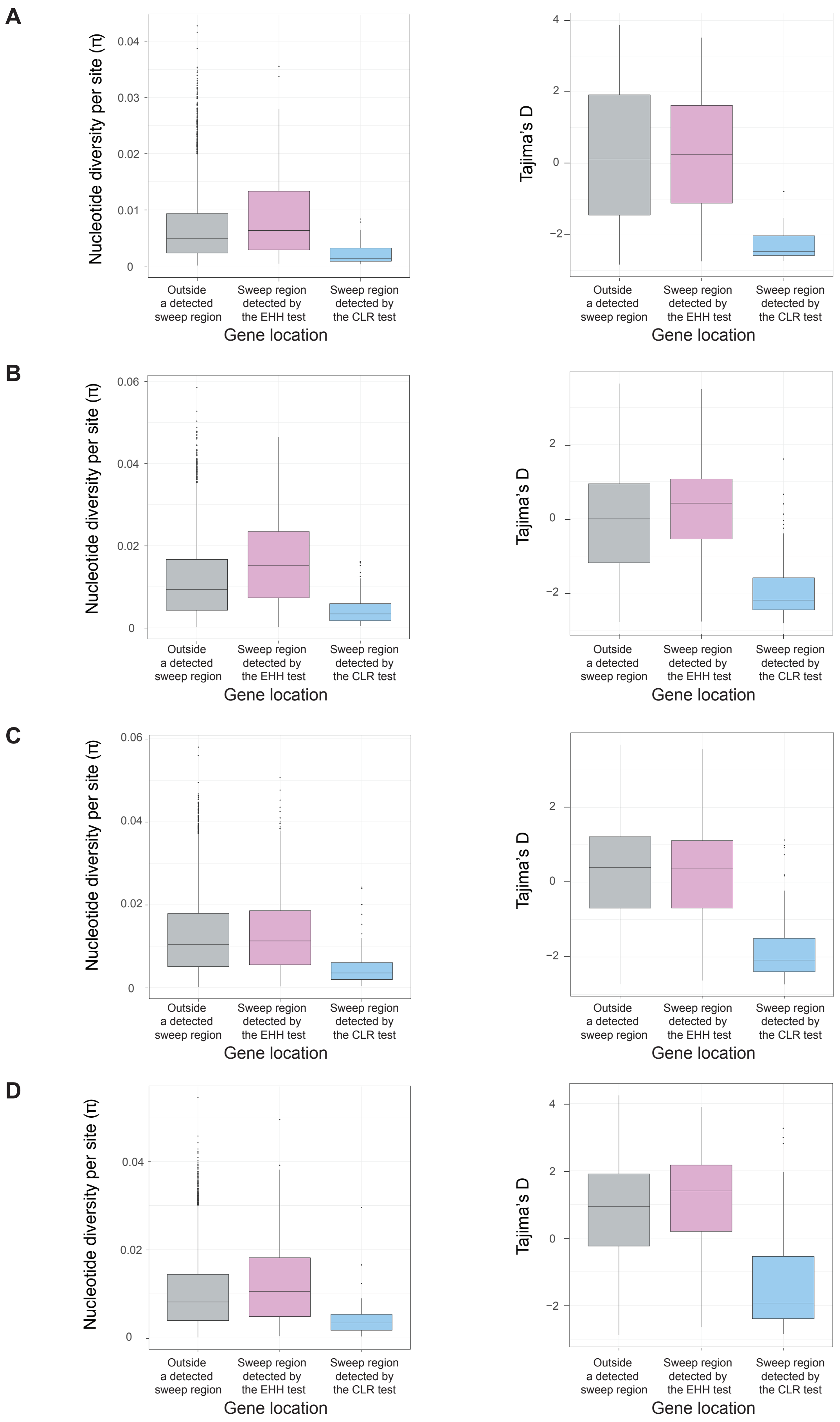

Fig. S4: Distribution of per gene nucleotide diversity per site (π) and per gene Tajima's D statistics according to the gene location within a selective sweep region identified in the composite likelihood ratio (CLR) test and the extended haplotype homozygosity (EHH) test or outside a detected selective sweep region. Distribution of the statistics is shown for the Australia (A), Swiss (B), Israel (C) and Oregon (D) populations.

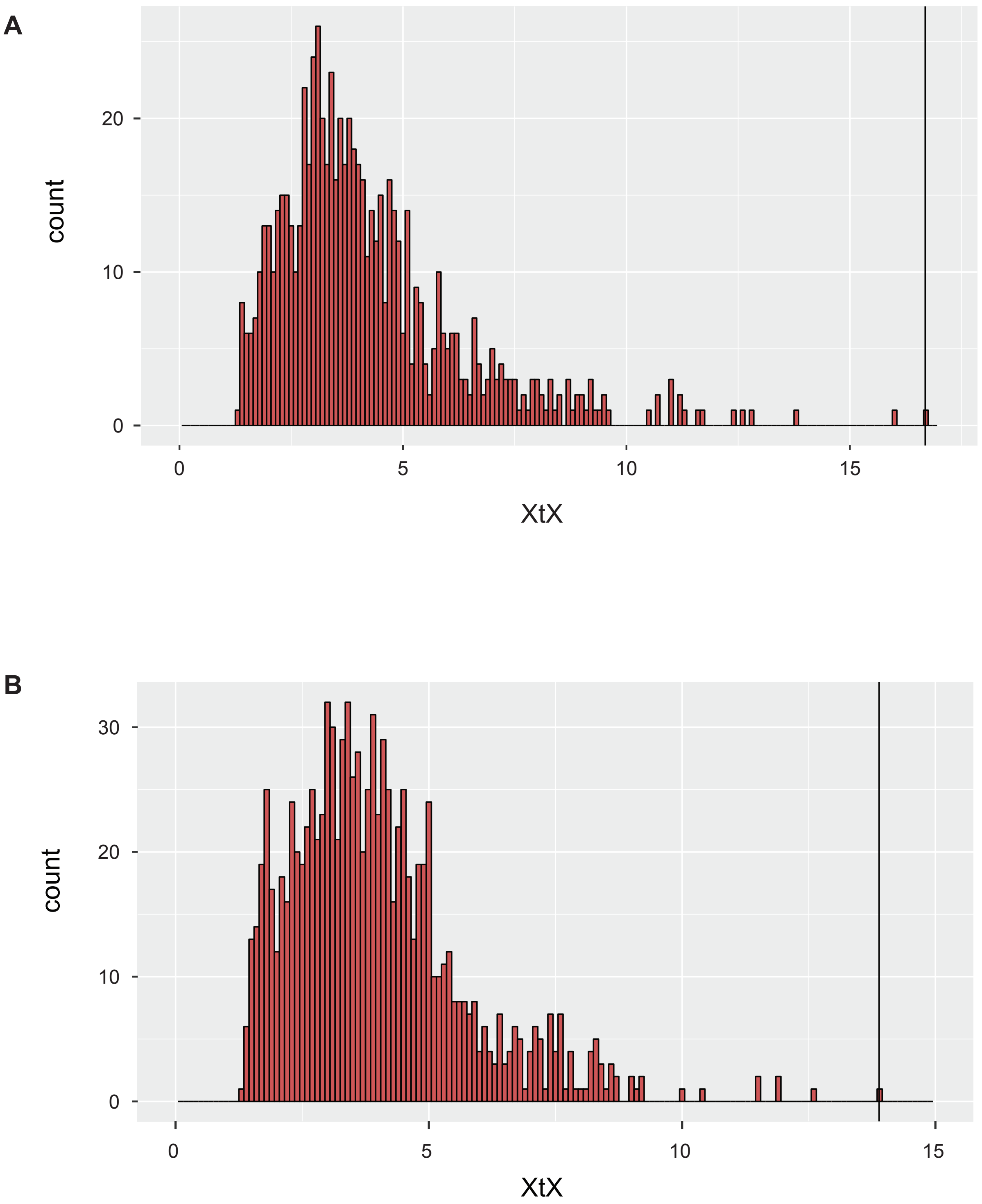

Fig. S5: Distribution of XtX statistics values among the four allopatric *Zymoseptoria tritici* populations obtained (A) for randomly selected genome-wide distributed synonymous SNPs and (B) computed using a simulated pseudo observed dataset. In each panel, the vertical line shows the maximum XtX statistics value of the distribution. The highest maximum XtX statistics value was used as a threshold to define differentiation outlier SNPs.

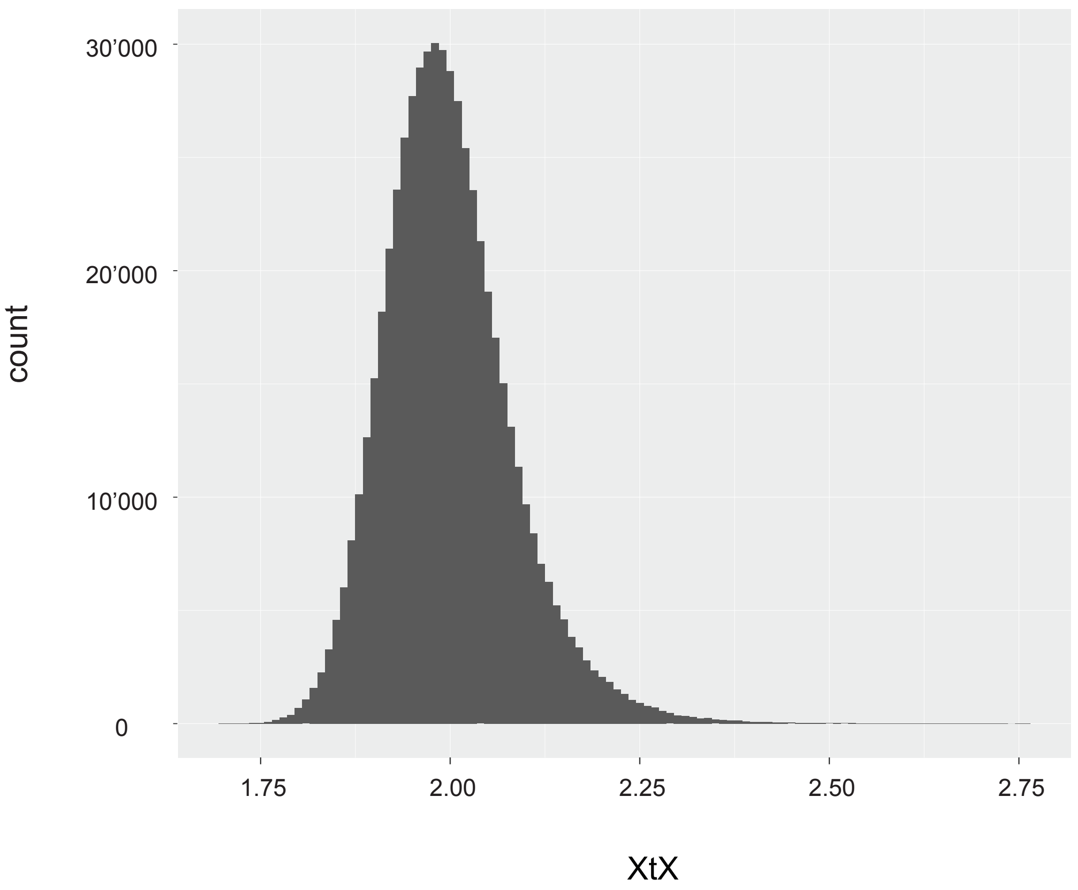

Fig. S6: Distribution of XtX statistics values among the two Oregon sympatric *Zymoseptoria tritici* populations.

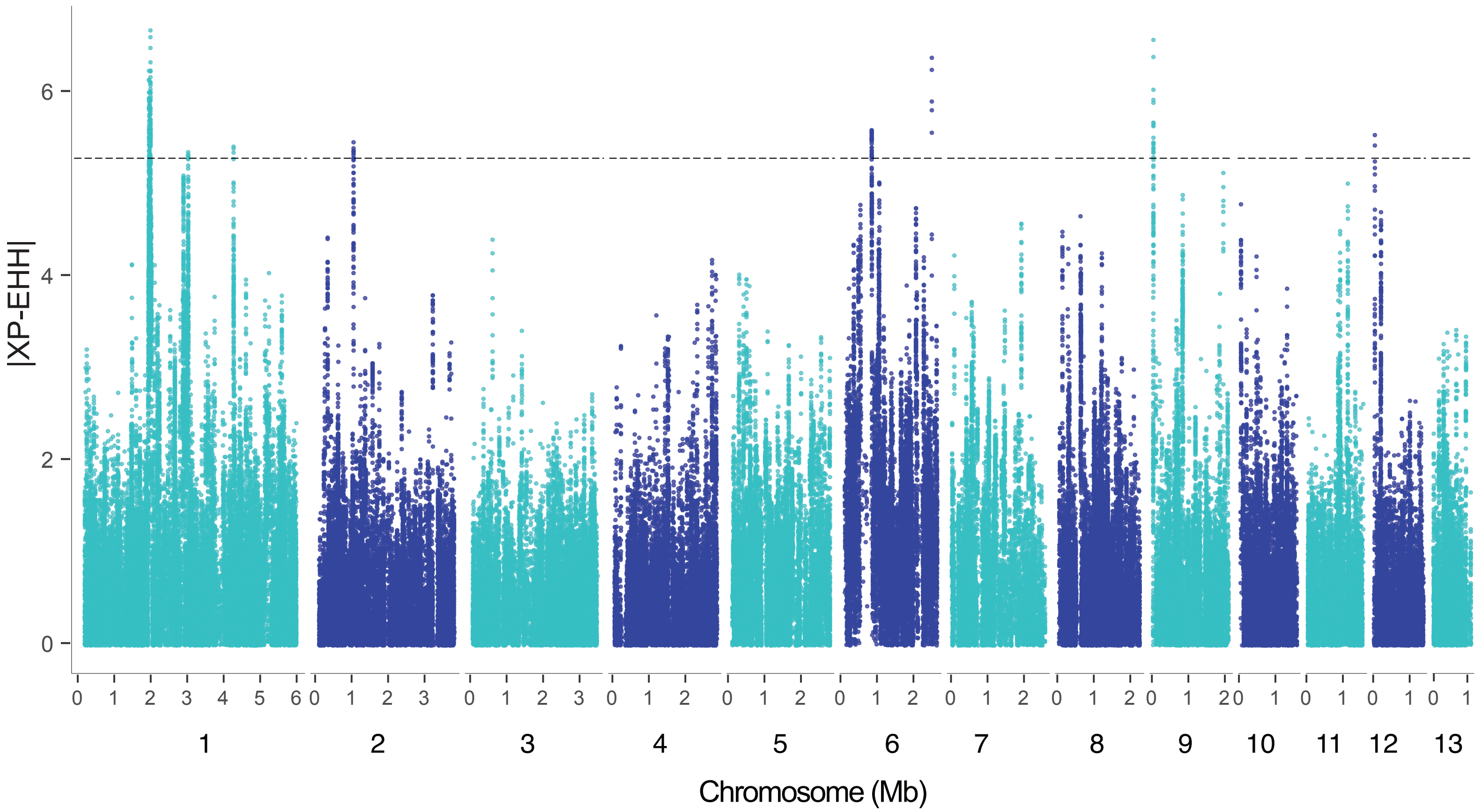

Fig. S7: Cross-population extended haplotype homozygosity (XP-EHH) scan among the two Oregon sympatric *Zymoseptoria tritici* populations. The horizontal dashed line shows the 99.9% percentile of the distribution of absolute XP-EHH values that was used as an outlier detection threshold.

Table S1: Accession numbers and statistics for the whole-genome sequencing data of 123 *Zymoseptoria tritici* isolates used in this study.

Isolate ID	Population	Reads length	Number of reads (1)	Alignment rate (%)	Mean coverage at mapped positions (X) (2)	Bioproject ID (3)	BioSample ID (4)
ISY_Ar_11g	Israel	101	7178306	94	17	PRJNA327615	SAMN05353914
ISY_Ar_11i	Israel	101	3434300	94	8	PRJNA327615	SAMN05353902
ISY_Ar_12d	Israel	101	4691441	94	11	PRJNA327615	SAMN05353911
ISY_Ar_12e	Israel	101	4374893	94	10	PRJNA327615	SAMN05353903
ISY_Ar_12f	Israel	101	9933383	93	24	PRJNA327615	SAMN05353908
ISY_Ar_15a	Israel	101	5473738	94	13	PRJNA327615	SAMN05353913
ISY_Ar_16a	Israel	101	4053420	94	10	PRJNA327615	SAMN05353900
ISY_Ar_16h	Israel	101	11095379	94	27	PRJNA327615	SAMN05353904
ISY_Ar_17b	Israel	101	11609906	93	27	PRJNA327615	SAMN05353907
ISY_Ar_17e	Israel	101	5353576	94	13	PRJNA327615	SAMN05353897
ISY_Ar_17i	Israel	101	6545538	94	16	PRJNA327615	SAMN05353912
ISY_Ar_17r	Israel	101	7954755	94	19	PRJNA327615	SAMN05353905
ISY_Ar_18b	Israel	101	10378790	93	25	PRJNA327615	SAMN05353906
ISY_Ar_19e	Israel	101	7352203	94	18	PRJNA327615	SAMN05981206
ISY_Ar_1b	Israel	101	4257294	94	10	PRJNA327615	SAMN05353901
ISY_Ar_1j	Israel	101	3803413	94	9	PRJNA327615	SAMN05981207
ISY_Ar_21a	Israel	101	8032270	94	19	PRJNA327615	SAMN05353910
ISY_Ar_22f	Israel	101	5431106	92	13	PRJNA327615	SAMN05353898
ISY_Ar_2b	Israel	101	4112730	94	10	PRJNA327615	SAMN05981208
ISY_Ar_2f	Israel	101	9155937	93	22	PRJNA327615	SAMN05353909
ISY_Ar_4b	Israel	101	9983818	94	24	PRJNA327615	SAMN05981209
ISY_Ar_4f	Israel	101	10087507	94	24	PRJNA327615	SAMN05981210
ISY_Ar_4g	Israel	101	3855471	94	9	PRJNA327615	SAMN05981211
ISY_Ar_5g	Israel	101	6609914	95	16	PRJNA327615	SAMN05981212
ST99CH_3A1	Switzerland	101	4234916	95	10	PRJNA327615	SAMN05353973
ST99CH_3A10	Switzerland	101	4677841	94	11	PRJNA327615	SAMN05981213
ST99CH_3A2	Switzerland	101	4589868	94	11	PRJNA327615	SAMN05353963
ST99CH_3A4	Switzerland	101	4732903	94	11	PRJNA327615	SAMN05353964
ST99CH_3A5	Switzerland	101	6041691	95	15	PRJNA327615	SAMN05353965
ST99CH_3A6	Switzerland	101	11052530	95	27	PRJNA327615	SAMN05353968
ST99CH_3A9	Switzerland	101	8270729	95	20	PRJNA327615	SAMN05981214
ST99CH_3B2	Switzerland	101	9671259	95	23	PRJNA327615	SAMN05353971
ST99CH_3B4	Switzerland	101	12191052	94	29	PRJNA327615	SAMN05353969
ST99CH_3B8	Switzerland	90	6140418	86	12	PRJNA178194	SAMN01815823
ST99CH_3C7	Switzerland	101	4926215	95	12	PRJNA327615	SAMN05353966
ST99CH_3D1	Switzerland	90	5651343	83	11	PRJNA178194	SAMN01815821
ST99CH_3D3	Switzerland	101	5794133	94	14	PRJNA327615	SAMN05353960
ST99CH_3D5	Switzerland	101	4810676	93	11	PRJNA327615	SAMN05353962
ST99CH_3D7	Switzerland	90	5809671	87	11	PRJNA178194	SAMN01815820
ST99CH_3D8	Switzerland	101	8194434	95	20	PRJNA327615	SAMN05981215
ST99CH_3F1	Switzerland	101	7937361	95	19	PRJNA327615	SAMN05353970
ST99CH_3F2	Switzerland	101	5423466	95	13	PRJNA327615	SAMN05353974
ST99CH_3F3	Switzerland	101	4670709	94	11	PRJNA327615	SAMN05353967
ST99CH_3F4	Switzerland	101	3943885	94	9	PRJNA327615	SAMN05981216
ST99CH_3F5	Switzerland	90	5776727	88	12	PRJNA178194	SAMN01815819
ST99CH_3G2	Switzerland	101	7267314	95	18	PRJNA327615	SAMN05981217
ST99CH_3G3	Switzerland	101	5765933	95	14	PRJNA327615	SAMN05981218
ST99CH_3G6	Switzerland	101	6420297	94	15	PRJNA327615	SAMN05353972
ST99CH_3H1	Switzerland	101	5414769	94	13	PRJNA327615	SAMN05353961
ST99CH_3H3	Switzerland	101	7641211	94	18	PRJNA327615	SAMN05353976
ST99CH_3H4	Switzerland	101	9678529	95	23	PRJNA327615	SAMN05353975
STAus01_1A4	Australia	101	5029770	95	12	PRJNA327615	SAMN05353885
STAus01_1A5	Australia	101	4376151	95	11	PRJNA327615	SAMN05353878
STAus01_1A6	Australia	101	5440046	94	13	PRJNA327615	SAMN05353896
STAus01_1A9	Australia	101	3710617	95	9	PRJNA327615	SAMN05353890
STAus01_1B1	Australia	101	5761703	95	14	PRJNA327615	SAMN05981219
STAus01_1B2	Australia	101	3918651	94	9	PRJNA327615	SAMN05353883
STAus01_1B7	Australia	101	7402701	94	18	PRJNA327615	SAMN05353877
STAus01_1B8	Australia	101	6776870	95	16	PRJNA327615	SAMN05353887
STAus01_1C1	Australia	101	7005709	95	17	PRJNA327615	SAMN05981220
STAus01_1C2	Australia	101	4083035	95	10	PRJNA327615	SAMN05353882
STAus01_1C3	Australia	101	10146056	95	25	PRJNA327615	SAMN05981221
STAus01_1C6	Australia	101	4793741	95	12	PRJNA327615	SAMN05981222
STAus01_1C8	Australia	101	4727151	94	11	PRJNA327615	SAMN05353880
STAus01_1D4	Australia	101	3495583	95	8	PRJNA327615	SAMN05353889
STAus01_1D5	Australia	101	4484033	95	11	PRJNA327615	SAMN05353893
STAus01_1D8	Australia	101	6341542	95	15	PRJNA327615	SAMN05353894
STAus01_1E1	Australia	101	4987210	95	12	PRJNA327615	SAMN05353892
STAus01_1E5	Australia	101	3692556	95	9	PRJNA327615	SAMN05353879
STAus01_1F2	Australia	101	4209252	95	10	PRJNA327615	SAMN05353884
STAus01_1F3	Australia	101	5704378	95	14	PRJNA327615	SAMN05981223
STAus01_1F8	Australia	101	6190980	95	15	PRJNA327615	SAMN05353895
STAus01_1G2	Australia	101	7152893	95	17	PRJNA327615	SAMN05981224
STAus01_1G5	Australia	101	5013052	95	12	PRJNA327615	SAMN05353886
STAus01_1H2	Australia	101	4284100	94	10	PRJNA327615	SAMN05353881
STAus01_1H6	Australia	101	4102884	94	10	PRJNA327615	SAMN05353888
STAus01_1H8	Australia	101	5407620	95	13	PRJNA327615	SAMN05353891
a12_3B_12	Oregon	101	10630698	95	26	PRJNA327615	SAMN05353929
a12_3B_14	Oregon	101	4755359	94	11	PRJNA327615	SAMN05353933
a12_3B_15	Oregon	101	4746231	94	11	PRJNA327615	SAMN05353916
a12_3B_17	Oregon	101	10476682	95	25	PRJNA327615	SAMN05353928
a12_3B_18	Oregon	101	9785365	93	23	PRJNA327615	SAMN05353927
a12_3B_19	Oregon	101	4937679	93	12	PRJNA327615	SAMN05353934
a12_3B_2	Oregon	101	3640816	95	9	PRJNA327615	SAMN05353917
a12_3B_21	Oregon	101	5307442	95	13	PRJNA327615	SAMN05353915
a12_3B_3	Oregon	101	3760709	95	9	PRJNA327615	SAMN05353926
a12_3B_5	Oregon	101	4734412	95	11	PRJNA327615	SAMN05353923
a12_3B_6	Oregon	101	4317174	95	10	PRJNA327615	SAMN05353932
a12_3B_7	Oregon	101	5117420	95	12	PRJNA327615	SAMN05353922
a12_3B_8	Oregon	101	3909637	95	9	PRJNA327615	SAMN05353924
a12_4A_1	Oregon	101	5552208	95	13	PRJNA327615	SAMN05353935
a12_4A_10	Oregon	101	4390406	94	10	PRJNA327615	SAMN05353920
a12_4A_11	Oregon	101	9226181	94	22	PRJNA327615	SAMN05353930
a12_4A_2	Oregon	101	3308123	95	8	PRJNA327615	SAMN05353925
a12_4A_4	Oregon	101	6243269	95	15	PRJNA327615	SAMN05353921
a12_4A_5	Oregon	101	3926832	95	9	PRJNA327615	SAMN05353918
a12_4A_6	Oregon	101	6877734	95	17	PRJNA327615	SAMN05981225
a12_4A_7	Oregon	101	4598742	95	11	PRJNA327615	SAMN05353919
a15_2A_11	Oregon	101	9382130	96	23	PRJNA327615	SAMN05353952
a15_2A_13	Oregon	101	4373040	95	11	PRJNA327615	SAMN05353949
a15_2A_16	Oregon	101	6935509	96	17	PRJNA327615	SAMN05353957
a15_2A_20	Oregon	101	6847234	95	17	PRJNA327615	SAMN05981227

a15_2A_6	Oregon	101	5570290	95	13	PRJNA327615	SAMN05353939
a15_2A_7	Oregon	101	6271214	95	15	PRJNA327615	SAMN05353958
a15_3B_13	Oregon	101	5448087	95	13	PRJNA327615	SAMN05353959
a15_3B_15	Oregon	101	4528385	95	11	PRJNA327615	SAMN05353940
a15_3B_18	Oregon	101	3767412	95	9	PRJNA327615	SAMN05353946
a15_3B_19	Oregon	101	4894289	94	12	PRJNA327615	SAMN05353955
a15_3B_3	Oregon	101	4369543	95	11	PRJNA327615	SAMN05981228
a15_3B_4	Oregon	101	4202703	94	10	PRJNA327615	SAMN05353942
a15_3B_5	Oregon	101	4977444	94	12	PRJNA327615	SAMN05353943
a15_3B_6	Oregon	101	6975988	94	17	PRJNA327615	SAMN05353950
a15_3B_9	Oregon	101	5255950	95	13	PRJNA327615	SAMN05353936
a15_4A_10	Oregon	101	8079466	95	20	PRJNA327615	SAMN05353956
a15_4A_11	Oregon	101	6959194	95	17	PRJNA327615	SAMN05981229
a15_4A_13	Oregon	101	3807392	95	9	PRJNA327615	SAMN05353938
a15_4A_15	Oregon	101	4446460	95	11	PRJNA327615	SAMN05353948
a15_4A_17	Oregon	101	4503275	95	11	PRJNA327615	SAMN05353945
a15_4A_19	Oregon	101	4408256	95	11	PRJNA327615	SAMN05353944
a15_4A_2	Oregon	101	6298950	95	15	PRJNA327615	SAMN05353951
a15_4A_3	Oregon	101	4870163	95	12	PRJNA327615	SAMN05353954
a15_4A_4	Oregon	101	9371938	96	23	PRJNA327615	SAMN05353953
a15_4A_7	Oregon	101	4087540	95	10	PRJNA327615	SAMN05353937

(1) Number of reads after trimming step
(2) The genome-wide coverage was calculated as the number of mapped reads multiplied by average read length and divided by the genome size
(3) (Croll et al, 2013; Hartmann et al, 2017)
(4) All samples are available on the NCBI Short Read Archive

Table S2: Genetic diversity statistics of the four allopatric *Zymoseptoria tritici* populations.

The numbers of bi-allelic SNPs according to different categories (genic, non-genic, with determined ancestral allele) and mean values of gene-wise nucleotide diversity per site (π) and Tajima's D are shown.

Statistics	123 isolates	Australia (n=26)	Switzerland (n=27)	Israel (n=24)	Oregon (n=46)
Number of SNPs:					
All SNPs	1,375,999	311,453	982,964	936,025	712,075
Genic SNPs	759,268	179,293	554,933	537,225	407,189
Non-genic SNPs	616,731	132,160	428,031	398,800	304,886
SNPs with determined ancestral allele	584,327	123,169	408,603	383,496	284,358
Per gene nucleotide diversity per site (π)					
median value	-	0.00489	0.00931	0.01033	0.00819
mean value	-	0.00682	0.01133	0.01236	0.01014
Per gene Tajima's D					
median value	-	0.12150	-0.01031	0.37205	0.95050
mean value	-	0.24360	-0.10991	0.23350	0.81187

Table S3: Summary statistics of the selective sweep regions identified in the *Zymoseptoria tritici* population sampled in Australia.

Selective sweep regions detected using the composite likelihood ratio (CLR) test and the extended haplotype homozygosity (EHH) test are shown.
Mean and median values of gene-wise nucleotide diversity per site (π) and Tajima's D in selective sweep regions are shown.

Sweep region ID	Chromosome	Start position	Stop position	Number of genes	Scan	Mean value of per gene nucleotide diversity per site (π)	Median value of per gene nucleotide diversity per site (π)	Mean value of per gene Tajima's D	Median value of per gene Tajima's D
1_607946_608193	1	607946	608193	1	EHH test	0.01617	0.01617	-0.36586	-0.36586
1_1063757_1063865	1	1063757	1063865	1	EHH test	0.01231	0.01231	0.83821	0.83821
1_1143353_1201130	1	1143353	1201130	13	EHH test	0.00308	0.00230	-0.44408	0.09669
1_1926500_1945664	1	1926500	1945664	8	EHH test	0.00624	0.00394	1.66553	2.02213
1_1939847_1980099	1	1939847	1980099	17	EHH test	0.00465	0.00387	1.30600	1.05158
1_2083273_2175351	1	2083273	2175351	37	EHH test	0.00585	0.00487	0.87523	1.12068
1_2632515_2649720	1	2632515	2649720	8	EHH test	0.00521	0.00510	0.22725	0.58053
1_2757393_2835405	1	2757393	2835405	14	CLR test	0.00170	0.00131	-2.20063	-2.50440
1_2898422_2929425	1	2898422	2929425	13	CLR test	0.00086	0.00073	-2.19003	-2.37905
1_4184521_4185400	1	4184521	4185400	2	EHH test	0.00625	0.00625	-0.60895	-0.60895
1_4447480_4448108	1	4447480	4448108	1	EHH test	0.00417	0.00417	-0.52095	-0.52095
1_5455027_5459610	1	5455027	5459610	2	EHH test	0.01085	0.01085	2.92617	2.92617
2_550120_566439	2	550120	566439	8	EHH test	0.00749	0.00632	-0.56993	-0.54403
2_2088403_2088936	2	2088403	2088936	1	EHH test	0.00955	0.00955	0.59441	0.59441
2_3659032_3659113	2	3659032	3659113	1	EHH test	0.01439	0.01439	-1.66069	-1.66069
3_341848_342172	3	341848	342172	1	EHH test	0.02776	0.02776	2.81958	2.81958
3_345388_345397	3	345388	345397	1	EHH test	0.01556	0.01556	2.86229	2.86229
3_1252650_1285656	3	1252650	1285656	15	CLR test	0.00070	0.00062	-2.54993	-2.53071
3_1293667_1322671	3	1293667	1322671	11	CLR test	0.00361	0.00298	-2.05295	-2.14316
3_1336684_1367689	3	1336684	1367689	11	CLR test	0.00556	0.00417	-1.76087	-2.20743
3_1337859_1337911	3	1337859	1337911	1	EHH test	0.00837	0.00837	-2.20743	-2.20743
3_1339300_1339484	3	1339300	1339484	0	EHH test	NA	NA	NA	NA
3_1907116_1908910	3	1907116	1908910	2	EHH test	0.00346	0.00346	-0.38811	-0.38811
3_1909007_1909717	3	1909007	1909717	1	EHH test	0.00330	0.00330	0.25226	0.25226
3_1949074_1954581	3	1949074	1954581	2	EHH test	0.00885	0.00885	0.57972	0.57972
3_2216224_2296002	3	2216224	2296002	30	EHH test	0.00304	0.00227	-1.24102	-2.34658
3_3044055_3094861	3	3044055	3094861	22	EHH test	0.00621	0.00426	-0.85733	-1.12099
3_3207587_3227385	3	3207587	3227385	9	EHH test	0.00616	0.00442	-1.44591	-2.56506
3_3250116_3250152	3	3250116	3250152	0	EHH test	NA	NA	NA	NA
5_593785_612861	5	593785	612861	7	EHH test	0.01352	0.00907	2.63322	2.67268
5_847891_898905	5	847891	898905	23	CLR test	0.00229	0.00154	-2.42378	-2.47175
5_1353185_1367656	5	1353185	1367656	5	EHH test	0.00916	0.00841	0.30239	0.88408
5_1375388_1375702	5	1375388	1375702	0	EHH test	NA	NA	NA	NA
5_1376090_1380871	5	1376090	1380871	3	EHH test	0.00334	0.00334	-0.84976	-0.84976
5_1558078_1564346	5	1558078	1564346	3	EHH test	0.00157	0.00157	-0.11023	-0.11023
5_2297614_2305710	5	2297614	2305710	3	EHH test	NA	NA	NA	NA
6_267566_289614	6	267566	289614	8	EHH test	0.00899	0.00615	0.11488	0.36289
6_2515602_2515741	6	2515602	2515741	1	EHH test	0.02118	0.02118	2.07462	2.07462
7_229237_229376	7	229237	229376	1	EHH test	0.00591	0.00591	2.52291	2.52291
7_489375_490263	7	489375	490263	2	EHH test	0.01263	0.01263	0.65355	0.65355
7_1924781_1925233	7	1924781	1925233	1	EHH test	0.00659	0.00659	-1.16869	-1.16869
7_2132112_2132422	7	2132112	2132422	1	EHH test	0.01766	0.01766	1.56779	1.56779
7_2422416_2457963	7	2422416	2457963	18	EHH test	0.01192	0.01067	0.92892	1.06514
8_926308_990377	8	926308	990377	32	EHH test	0.00841	0.00649	1.18264	1.06933
8_1081650_1086204	8	1081650	1086204	3	EHH test	0.00802	0.00571	0.45987	0.32171
8_1740264_1740337	8	1740264	1740337	0	EHH test	NA	NA	NA	NA
8_1957912_1957927	8	1957912	1957927	1	EHH test	0.02096	0.02096	3.49632	3.49632
9_986453_986683	9	986453	986683	1	EHH test	0.01323	0.01323	0.73070	0.73070
10_1488654_1489226	10	1488654	1489226	1	EHH test	0.01338	0.01338	1.09586	1.09586
11_352788_424014	11	352788	424014	22	EHH test	0.00564	0.00231	0.54569	0.63502
11_442207_442228	11	442207	442228	1	EHH test	0.01657	0.01657	0.79136	0.79136
11_648290_649243	11	648290	649243	1	EHH test	0.02362	0.02362	3.47719	3.47719
11_896526_897552	11	896526	897552	1	EHH test	0.01177	0.01177	2.02181	2.02181
11_949430_973533	11	949430	973533	9	EHH test	0.01128	0.01002	1.65499	2.54456
12_528748_528796	12	528748	528796	1	EHH test	0.03555	0.03555	0.57330	0.57330
12_528796_528892	12	528796	528892	1	EHH test	0.03555	0.03555	0.57330	0.57330
12_528896_528931	12	528896	528931	1	EHH test	0.03555	0.03555	0.57330	0.57330
12_753193_780502	12	753193	780502	7	EHH test	0.00959	0.00671	-0.48589	-0.73391
12_788313_816054	12	788313	816054	15	EHH test	0.01634	0.01602	0.35861	0.06404
12_804888_839046	12	804888	839046	14	EHH test	0.01064	0.00850	-0.34349	-0.46082

Table S4: Summary statistics of the selective sweep regions identified in the *Zymoseptoria tritici* population sampled in Switzerland.
Selective sweep regions detected using the composite likelihood ratio (CLR) test and the extended haplotype homozygosity (EHH) test are shown.
Mean and median values of gene-wise nucleotide diversity per site (π) and Tajima's D in selective sweep regions are shown.

Sweep region ID	Chromosome	Start position	Stop position	Number of genes	Scan	Mean value of per gene nucleotide diversity per site (π)	Median value of per gene nucleotide diversity per site (π)	Mean value of per gene Tajima's D	Median value of per gene Tajima's D
1_1430994_1440595	1	1430994	1440595	2	EHH test	0.02052	0.02052	0.39328	0.39328
1_1449373_1449404	1	1449373	1449404	0	EHH test	NA	NA	NA	NA
1_2114193_2124595	1	2114193	2124595	5	CLR test	0.00523	0.00348	-1.56055	-1.59758
1_2129198_2136599	1	2129198	2136599	4	CLR test	0.00181	0.00160	-1.37482	-1.32295
1_4351914_4355364	1	4351914	4355364	3	EHH test	0.01787	0.01787	1.77553	1.77553
1_4546742_4550083	1	4546742	4550083	2	EHH test	0.01568	0.01568	0.52653	0.52653
1_4634943_4639343	1	4634943	4639343	2	CLR test	0.00422	0.00422	-2.08317	-2.08317
1_4641945_4646345	1	4641945	4646345	2	CLR test	0.00270	0.00270	-1.72800	-1.72800
1_4820928_4821123	1	4820928	4821123	1	EHH test	0.01512	0.01512	0.30535	0.30535
1_5473192_5486594	1	5473192	5486594	7	CLR test	0.00386	0.00405	-1.88443	-2.27800
1_5492339_5493365	1	5492339	5493365	1	EHH test	0.01707	0.01707	0.43149	0.43149
1_5494461_5494462	1	5494461	5494462	0	EHH test	NA	NA	NA	NA
1_5494479_5494634	1	5494479	5494634	1	EHH test	0.02259	0.02259	1.61426	1.61426
1_5495454_5495537	1	5495454	5495537	1	EHH test	0.02259	0.02259	1.61426	1.61426
1_5495690_5495713	1	5495690	5495713	1	EHH test	0.02259	0.02259	1.61426	1.61426
1_5495713_5495794	1	5495713	5495794	1	EHH test	0.02259	0.02259	1.61426	1.61426
1_5495842_5495863	1	5495842	5495863	1	EHH test	0.02259	0.02259	1.61426	1.61426
1_5510137_5616031	1	5510137	5616031	34	EHH test	0.01582	0.01425	0.48251	0.81365
1_5750274_5754674	1	5750274	5754674	1	CLR test	0.00454	0.00454	-2.21564	-2.21564
2_382208_408545	2	382208	408545	5	EHH test	0.01088	0.01088	-1.33544	-1.33544
2_993633_993784	2	993633	993784	1	EHH test	0.01555	0.01555	0.49337	0.49337
2_1957328_1957569	2	1957328	1957569	0	EHH test	NA	NA	NA	NA
2_2102304_2102770	2	2102304	2102770	1	EHH test	0.01485	0.01485	0.98880	0.98880
2_3156180_3157465	2	3156180	3157465	1	EHH test	0.01631	0.01631	1.38913	1.38913
2_3695420_3707872	2	3695420	3707872	2	EHH test	0.01965	0.01965	0.56365	0.56365
2_3722790_3722875	2	3722790	3722875	1	EHH test	0.02391	0.02391	0.73309	0.73309
2_3759287_3777235	2	3759287	3777235	6	EHH test	0.01784	0.01435	-0.02055	-0.11284
3_520299_529530	3	520299	529530	3	EHH test	0.01924	0.01924	1.11633	1.11633
3_841414_861820	3	841414	861820	7	CLR test	0.00540	0.00406	-1.61594	-1.75247
3_1971197_2011438	3	1971197	2011438	12	EHH test	0.00531	0.00432	-1.31348	-1.63370
3_1979848_1993251	3	1979848	1993251	5	CLR test	0.00432	0.00432	-1.53410	-1.53410
3_2688118_2694518	3	2688118	2694518	4	CLR test	0.00805	0.00581	-1.40109	-1.79900
3_3155296_3178703	3	3155296	3178703	12	CLR test	0.00316	0.00225	-2.34289	-2.32991
4_442462_452864	4	442462	452864	6	CLR test	0.00430	0.00456	-1.83142	-1.95571
4_536500_548904	4	536500	548904	2	CLR test	0.00128	0.00128	-2.62072	-2.62072
4_709248_709417	4	709248	709417	1	EHH test	0.02992	0.02992	1.18856	1.18856
4_2294235_2295140	4	2294235	2295140	1	EHH test	0.04644	0.04644	1.78529	1.78529
4_2401264_2409665	4	2401264	2409665	2	CLR test	0.00439	0.00439	-2.62205	-2.62205
4_2556363_2557138	4	2556363	2557138	1	EHH test	0.02542	0.02542	1.31560	1.31560
4_2706788_2716248	4	2706788	2716248	6	EHH test	0.02258	0.02371	0.55305	0.20885
4_2716229_2724108	4	2716229	2724108	5	EHH test	0.01410	0.00844	-0.41182	-0.27817
4_2728347_2728895	4	2728347	2728895	1	EHH test	0.02013	0.02013	-0.70929	-0.70929
4_2742944_2742975	4	2742944	2742975	1	EHH test	0.02768	0.02768	0.30243	0.30243
4_2743812_2743854	4	2743812	2743854	1	EHH test	0.02768	0.02768	0.30243	0.30243
4_2765792_2770924	4	2765792	2770924	2	EHH test	0.02514	0.02514	0.65355	0.65355
4_2801656_2808395	4	2801656	2808395	3	EHH test	0.02998	0.02998	0.68921	0.68921
4_2824437_2832839	4	2824437	2832839	5	CLR test	0.00309	0.00286	-2.48522	-2.47473
5_123369_127769	5	123369	127769	1	CLR test	0.00939	0.00939	-1.31180	-1.31180
5_273879_283114	5	273879	283114	6	EHH test	0.02824	0.02824	0.52105	0.52105
5_1456028_1466431	5	1456028	1466431	5	CLR test	0.00126	0.00063	-1.97713	-1.93917
5_1648339_1650360	5	1648339	1650360	2	EHH test	0.00824	0.00824	-1.10060	-1.10060
5_1722806_1725697	5	1722806	1725697	3	EHH test	0.01432	0.01495	1.24497	1.00443
5_2120287_2120737	5	2120287	2120737	1	EHH test	0.00516	0.00516	-1.87366	-1.87366
5_2160687_2167129	5	2160687	2167129	3	EHH test	0.00210	0.00210	-2.22939	-2.22939
5_2306647_2319807	5	2306647	2319807	3	EHH test	0.00396	0.00396	-1.44074	-1.44074
5_2690452_2695439	5	2690452	2695439	2	EHH test	0.00338	0.00338	-0.75694	-0.75694
5_2734661_2739061	5	2734661	2739061	3	CLR test	0.00263	0.00148	-2.36348	-2.35399
5_2792114_2793086	5	2792114	2793086	1	EHH test	0.02058	0.02058	2.50063	2.50063
6_82159_101567	6	82159	101567	6	CLR test	0.00963	0.00877	-0.91456	-1.13850
6_217309_217436	6	217309	217436	1	EHH test	0.03115	0.03115	0.54413	0.54413
6_1113499_1113899	6	1113499	1113899	1	EHH test	0.01865	0.01865	0.86080	0.86080
6_1288740_1307042	6	1288740	1307042	8	EHH test	0.00645	0.00343	-1.50321	-1.79614
6_1496853_1526265	6	1496853	1526265	12	CLR test	0.00459	0.00401	-1.27912	-1.66595
6_1748206_1762529	6	1748206	1762529	2	EHH test	0.00861	0.00861	0.27730	0.27730
6_1885521_1885558	6	1885521	1885558	1	EHH test	0.01293	0.01293	-0.06669	-0.06669
6_1905446_1905494	6	1905446	1905494	1	EHH test	0.01224	0.01224	-1.01770	-1.01770
6_2491855_2507324	6	2491855	2507324	2	EHH test	0.03634	0.03634	0.79259	0.79259
6_2492343_2508456	6	2492343	2508456	3	EHH test	0.03248	0.02995	1.11957	0.93767
7_510166_516568	7	510166	516568	3	CLR test	0.00690	0.00690	-2.54814	-2.54814
7_1030917_1030995	7	1030917	1030995	1	EHH test	0.01971	0.01971	0.73760	0.73760
7_1105498_1176160	7	1105498	1176160	30	EHH test	0.01156	0.01108	0.50739	0.45563
7_1522822_1601782	7	1522822	1601782	31	EHH test	0.00234	0.00111	-0.92177	-1.19542
7_1996592_1996650	7	1996592	1996650	1	EHH test	0.02546	0.02546	0.65952	0.65952
7_2122776_2123957	7	2122776	2123957	2	EHH test	0.02345	0.02345	-0.78519	-0.78519
8_78095_86497	8	78095	86497	4	CLR test	0.00771	0.00760	-2.24140	-2.20876
8_197321_197459	8	197321	197459	1	EHH test	0.02518	0.02518	0.61421	0.61421
8_197667_197846	8	197667	197846	1	EHH test	0.02518	0.02518	0.61421	0.61421
8_207311_207355	8	207311	207355	1	EHH test	0.01134	0.01134	-0.10083	-0.10083
8_207336_207359	8	207336	207359	1	EHH test	0.01134	0.01134	-0.10083	-0.10083
8_929744_929786	8	929744	929786	0	EHH test	NA	NA	NA	NA
8_965499_969899	8	965499	969899	4	CLR test	0.00248	0.00245	-2.44604	-2.43151
8_1109548_1122594	8	1109548	1122594	6	EHH test	0.01593	0.01633	1.01660	1.32283
8_1760129_1765324	8	1760129	1765324	2	EHH test	0.02719	0.02719	0.50115	0.50115
8_1851904_1857304	8	1851904	1857304	2	CLR test	0.00611	0.00611	-2.14113	-2.14113
8_2025991_2031851	8	2025991	2031851	3	EHH test	0.00872	0.00731	-0.62819	-0.77086
8_2172782_2208640	8	2172782	2208640	6	EHH test	0.02825	0.02962	0.84215	1.38499
9_188167_188197	9	188167	188197	0	EHH test	NA	NA	NA	NA
9_485087_502978	9	485087	502978	9	EHH test	0.02091	0.01719	0.14024	0.75280
9_519766_519961	9	519766	519961	1	EHH test	0.02307	0.02307	0.06507	0.06507
9_817292_817786	9	817292	817786	1	EHH test	0.01860	0.01860	1.00696	1.00696
9_884864_900272	9	884864	900272	7	CLR test	0.00202	0.00195	-2.40517	-2.45859
9_942371_943822	9	942371	943822	1	EHH test	0.02074	0.02074	0.98902	0.98902
9_1117046_1123448	9	1117046	1123448	4	CLR test	0.00135	0.00094	-2.37010	-2.28143
9_1582412_1590816	9	1582412	1590816	3	CLR test	0.00489	0.00254	-2.31116	-2.32669
9_1714746_1728094	9	1714746	1728094	1	EHH test	0.00648	0.00648	0.26646	0.26646
9_1805425_1817614	9	1805425	1817614	5	EHH test	0.03214	0.03214	1.10145	1.10145
9_1817673_1817689	9	1817673	1817689	1	EHH test	0.03214	0.03214	1.10145	1.10145
10_345511_356810	10	345511	356810	5	EHH test	0.00832	0.00858	-0.69636	-0.75061
10_370517_380456	10	370517	380456	3	EHH test	0.01339	0.01339	0.70872	0.70872
10_531857_533446	10	531857	533446	1	EHH test	0.01615	0.01615	0.69807	0.69807
10_763054_774459	10	763054	774459	6	CLR test	0.00510	0.00432	-2.10235	-2.43122
10_834756_835315	10	834756	835315	1	EHH test	0.02246	0.02246	0.04866	0.04866
10_1119418_1119555	10	1119418	1119555	1	EHH test	0.02142	0.02142	2.01959	2.01959
10_1134641_1151708	10	1134641	1151708	3	EHH test	0.01473	0.01473	0.03220	0.03220
10_1137535_1152667	10	1137535	1152667	1	EHH test	0.02518	0.02518	0.91389	0.91389
10_1257304_1272274	10	1257304	1272274	2	EHH test	0.02423	0.02423	0.45546	0.45546
10_1457067_1457169	10	1457067	1457169	1	EHH test	0.02381	0.02381	0.77421	0.77421
10_1457226_1457310	10	1457226	1457310	1	EHH test	0.02381	0.02381	0.77421	0.77421
10_1488565_1488654	10	1488565	1488654	1	EHH test	0.02175	0.02175	1.85550	1.85550
10_1488848_1489154	10	1488848	1489154	1	EHH test	0.02175	0.02175	1.85550	1.85550
11_357236_378364	11	357236	378364	7	EHH test	0.01136	0.01081	-0.20425	-0.16112
11_563577_576989	11	563577	576989	8	CLR test	0.00602	0.00535	-1.57011	-1.89336
11_831639_844396	11	831639	844396	9	EHH test	0.01623	0.01731	-0.35198	-0.54101
11_870220_878597	11	870220	878597	3	EHH test	0.02232	0.01988	0.69190	0.33642
11_1093238_1098639	11	1093238	1098639	5	CLR test	0.00532	0.00480	-1.92010	-2.37330
11_1524559_1524658	11	1524559	1524658	1	EHH test	0.02691	0.02691	0.99778	0.99778
12_529099_535463	12	529099	535463	3	EHH test	0.04235	0.04235	0.32858	0.32858
12_653265_662833	12	653265	662833	3	EHH test	0.00907	0.00843	-0.14385	0.18673
13_841778_845596	13	841778	845596	1	EHH test	0.02939	0.02939	0.94425	0.94425

Table S5: Summary statistics of the selective sweep regions identified in the *Zymoseptoria tritici* population sampled in Israel.
Selective sweep regions detected using the composite likelihood ratio (CLR) test and the extended haplotype homozygosity (EHH) test are shown.
Mean and median values of gene-wise nucleotide diversity per site (π) and Tajima's D in selective sweep regions are shown.

Sweep region ID	Chromosome	Start position	Stop position	Number of genes	Scan	Mean value of per gene nucleotide diversity per site (π)	Median value of per gene nucleotide diversity per site (π)	Mean value of per gene Tajima's D	Median value of per gene Tajima's D
1_384888_384903	1	384888	384903	1	EHH test	0.02859	0.02859	0.43336	0.43336
1_675189_675229	1	675189	675229	1	EHH test	0.01772	0.01772	-0.42018	-0.42018
1_1013137_1013176	1	1013137	1013176	1	EHH test	0.02430	0.02430	0.99506	0.99506
1_1018535_1018838	1	1018535	1018838	1	EHH test	0.03097	0.03097	1.07842	1.07842
1_1146142_1163765	1	1146142	1163765	6	EHH test	0.00874	0.00767	0.64722	0.78225
1_1271814_1272095	1	1271814	1272095	1	EHH test	0.02724	0.02724	1.09686	1.09686
1_1273147_1273210	1	1273147	1273210	1	EHH test	0.02724	0.02724	1.09686	1.09686
1_1276119_1278736	1	1276119	1278736	3	EHH test	0.01966	0.01895	0.62468	0.79065
1_1278330_1367629	1	1278330	1367629	35	EHH test	0.00979	0.00837	0.12433	0.28678
1_1322958_1328358	1	1322958	1328358	3	CLR test	0.00184	0.00198	-2.30262	-2.27734
1_2280226_2297629	1	2280226	2297629	8	EHH test	0.01020	0.00947	-0.77994	-1.27302
1_2299248_2303648	1	2299248	2303648	2	CLR test	0.00433	0.00433	-2.33479	-2.33479
1_2907663_2907879	1	2907663	2907879	0	EHH test	NA	NA	NA	NA
1_2912431_2913701	1	2912431	2913701	2	EHH test	0.00800	0.00800	0.66407	0.66407
1_2974742_2975311	1	2974742	2975311	1	EHH test	0.02180	0.02180	2.80736	2.80736
1_3005708_3014752	1	3005708	3014752	5	EHH test	0.01037	0.01176	0.52591	0.45359
1_3014752_3015128	1	3014752	3015128	1	EHH test	0.01176	0.01176	0.45359	0.45359
1_3018002_3018102	1	3018002	3018102	1	EHH test	0.02092	0.02092	1.09663	1.09663
1_3018602_3019265	1	3018602	3019265	1	EHH test	0.01844	0.01844	1.41436	1.41436
1_3027223_3027381	1	3027223	3027381	1	EHH test	0.01260	0.01260	1.02839	1.02839
1_3047248_3047304	1	3047248	3047304	0	EHH test	NA	NA	NA	NA
1_3073958_3073976	1	3073958	3073976	1	EHH test	0.03061	0.03061	1.67111	1.67111
1_3075366_3075657	1	3075366	3075657	0	EHH test	NA	NA	NA	NA
1_3076114_3076141	1	3076114	3076141	1	EHH test	0.01855	0.01855	1.15169	1.15169
1_4724990_4725293	1	4724990	4725293	1	EHH test	0.03374	0.03374	0.96714	0.96714
1_4725349_4725401	1	4725349	4725401	1	EHH test	0.03374	0.03374	0.96714	0.96714
1_4725410_4727194	1	4725410	4727194	2	EHH test	0.02255	0.02255	0.96960	0.96960
1_4727617_4967899	1	4727617	4967899	92	EHH test	0.01400	0.01321	0.41676	0.52282
1_4728026_4733942	1	4728026	4733942	3	EHH test	0.02464	0.02801	1.27217	1.20049
1_4728205_4733585	1	4728205	4733585	2	EHH test	0.02001	0.02001	1.15365	1.15365
1_4733602_4789537	1	4733602	4789537	27	EHH test	0.01584	0.01505	0.61678	0.73290
1_4952997_5094902	1	4952997	5094902	47	EHH test	0.01141	0.01088	-0.19758	-0.28236
1_4989048_4994448	1	4989048	4994448	2	CLR test	0.00963	0.00963	-1.47532	-1.47532
1_5422702_5424445	1	5422702	5424445	2	EHH test	0.02702	0.02702	1.79445	1.79445
2_444285_444298	2	444285	444298	1	EHH test	0.01402	0.01402	0.96265	0.96265
2_972524_972563	2	972524	972563	1	EHH test	0.01204	0.01204	-0.57099	-0.57099
2_1009608_1014008	2	1009608	1014008	3	CLR test	0.00358	0.00359	-1.78392	-1.85133
2_1384805_1391206	2	1384805	1391206	3	CLR test	0.00481	0.00506	-1.55603	-2.32846
2_1832725_1832896	2	1832725	1832896	1	EHH test	0.03237	0.03237	0.90194	0.90194
2_2088435_2197034	2	2088435	2197034	42	EHH test	0.00883	0.00673	0.19132	0.52028
2_2197325_2197545	2	2197325	2197545	1	EHH test	0.01438	0.01438	1.50433	1.50433
2_2197545_2197566	2	2197545	2197566	1	EHH test	0.01438	0.01438	1.50433	1.50433
2_2836193_2836206	2	2836193	2836206	1	EHH test	0.02060	0.02060	1.35524	1.35524
2_2836725_3546490	2	2836725	3546490	230	EHH test	0.01025	0.00697	-0.29285	-0.44682
2_2836776_2836777	2	2836776	2836777	1	EHH test	0.02060	0.02060	1.35524	1.35524
2_2836887_2836911	2	2836887	2836911	1	EHH test	0.02060	0.02060	1.35524	1.35524
2_2875603_2887904	2	2875603	2887904	7	EHH test	0.00907	0.00675	-1.17216	-1.29630
2_2932619_2956029	2	2932619	2956029	7	CLR test	0.00254	0.00153	-1.89381	-1.89001
2_3328827_3333227	2	3328827	3333227	2	CLR test	0.00579	0.00579	-1.69397	-1.69397
2_3485425_3498037	2	3485425	3498037	5	EHH test	0.01241	0.00947	-0.95713	-1.60659
2_3539894_3542027	2	3539894	3542027	2	EHH test	0.02663	0.02663	0.69752	0.69752
2_3695581_3707199	2	3695581	3707199	1	EHH test	0.01466	0.01466	0.40812	0.40812
2_3789069_3794470	2	3789069	3794470	4	CLR test	0.00498	0.00498	-2.55944	-2.55944
3_464414_464433	3	464414	464433	1	EHH test	0.00688	0.00688	-1.39253	-1.39253
3_978250_983650	3	978250	983650	1	CLR test	0.00316	0.00316	-2.70303	-2.70303
3_1333443_1336396	3	1333443	1336396	2	EHH test	0.01095	0.01095	0.00769	0.00769
3_1774048_1774311	3	1774048	1774311	1	EHH test	0.02020	0.02020	2.10328	2.10328
3_1933092_1933133	3	1933092	1933133	0	EHH test	NA	NA	NA	NA
3_2037380_2296025	3	2037380	2296025	91	EHH test	0.00970	0.00714	-0.07760	-0.08687
3_2257812_2262212	3	2257812	2262212	3	CLR test	0.00566	0.00708	-2.05799	-2.08061
3_2404574_2404648	3	2404574	2404648	0	EHH test	NA	NA	NA	NA
3_2404665_2404707	3	2404665	2404707	0	EHH test	NA	NA	NA	NA
3_2404966_2405035	3	2404966	2405035	0	EHH test	NA	NA	NA	NA
3_2408854_2678837	3	2408854	2678837	80	EHH test	0.01019	0.00719	0.38595	0.51944
3_2689002_2693402	3	2689002	2693402	4	CLR test	0.00783	0.00281	-1.44241	-2.07929
3_3155207_3168611	3	3155207	3168611	6	CLR test	0.00483	0.00530	-1.95746	-1.90120
4_14286_24689	4	14286	24689	1	CLR test	0.00418	0.00418	-2.13940	-2.13940
4_600527_605927	4	600527	605927	2	CLR test	0.00768	0.00768	-1.65085	-1.65085
4_741511_791998	4	741511	791998	19	EHH test	0.01270	0.01064	0.70823	1.15320
4_1000145_1000756	4	1000145	1000756	1	EHH test	0.01171	0.01171	-0.92074	-0.92074
4_1169760_1202171	4	1169760	1202171	16	CLR test	0.00412	0.00335	-1.61947	-1.85553
4_1279805_1292208	4	1279805	1292208	5	CLR test	0.00099	0.00071	-1.78607	-1.85790
4_2279102_2294242	4	2279102	2294242	7	EHH test	0.01603	0.00885	-0.28083	0.00781
4_2295100_2298000	4	2295100	2298000	1	EHH test	0.04092	0.04092	0.79515	0.79515
4_2297891_2298000	4	2297891	2298000	0	EHH test	NA	NA	NA	NA
4_2401264_2417669	4	2401264	2417669	7	CLR test	0.00558	0.00491	-1.88992	-1.82040
4_2448149_2448976	4	2448149	2448976	2	EHH test	0.02283	0.02283	1.27298	1.27298
4_2471102_2474263	4	2471102	2474263	2	EHH test	0.01500	0.01500	0.98693	0.98693
4_2496426_2501067	4	2496426	2501067	2	EHH test	0.01033	0.01033	-0.34916	-0.34916
4_2501118_2511695	4	2501118	2511695	3	EHH test	0.01832	0.01832	-0.06384	-0.06384
4_2556164_2557138	4	2556164	2557138	1	EHH test	0.02649	0.02649	0.69896	0.69896
4_2713194_2723821	4	2713194	2723821	5	EHH test	0.02166	0.02370	0.92421	0.29381
4_2723827_2724601	4	2723827	2724601	1	EHH test	0.03659	0.03659	0.29381	0.29381
4_2726757_2743767	4	2726757	2743767	5	EHH test	0.02608	0.03056	0.56650	0.60766
5_2520937_2520970	5	2520937	2520970	1	EHH test	0.02612	0.02612	1.64247	1.64247
5_2521255_2657623	5	2521255	2657623	43	EHH test	0.01438	0.01420	0.46527	0.46008
5_2657953_2740054	5	2657953	2740054	23	EHH test	0.01571	0.01605	0.39943	0.44711
6_83223_90624	6	83223	90624	4	CLR test	0.01187	0.01232	-0.85032	-0.92904
6_217781_232345	6	217781	232345	3	EHH test	0.01689	0.01689	-0.35346	-0.35346
6_945382_1099152	6	945382	1099152	56	EHH test	0.00796	0.00703	0.23072	0.28002
6_1111794_1379276	6	1111794	1379276	90	EHH test	0.00855	0.00767	0.40087	0.66166
6_1198726_1215131	6	1198726	1215131	4	CLR test	0.00443	0.00332	-1.83235	-1.88031
6_1387977_1399411	6	1387977	1399411	5	EHH test	0.01440	0.01502	0.63438	0.31618
6_1506864_1514266	6	1506864	1514266	5	CLR test	0.00161	0.00160	-1.83277	-2.18196
6_1779438_1779504	6	1779438	1779504	1	EHH test	0.02342	0.02342	1.43610	1.43610
6_1835188_1835851	6	1835188	1835851	1	EHH test	0.01216	0.01216	1.71904	1.71904
6_1862679_1862724	6	1862679	1862724	1	EHH test	0.02519	0.02519	1.04599	1.04599
6_1872966_1873971	6	1872966	1873971	1	EHH test	0.01933	0.01933	0.50736	0.50736
6_1890898_1891058	6	1890898	1891058	0	EHH test	NA	NA	NA	NA
6_1891148_1891606	6	1891148	1891606	1	EHH test	0.01879	0.01879	1.16792	1.16792
7_1684202_1684877	7	1684202	1684877	0	EHH test	NA	NA	NA	NA
7_1853916_1854009	7	1853916	1854009	1	EHH test	0.01436	0.01436	-0.20549	-0.20549
7_2019251_2080719	7	2019251	2080719	23	EHH test	0.01539	0.01181	-0.06174	-0.31598
7_2081417_2085844	7	2081417	2085844	4	EHH test	0.02978	0.03229	0.44027	0.14420
7_2086792_2086893	7	2086792	2086893	1	EHH test	0.01592	0.01592	1.14388	1.14388
7_2192106_2192147	7	2192106	2192147	1	EHH test	0.00903	0.00903	0.02416	0.02416
7_2282011_2282035	7	2282011	2282035	1	EHH test	0.02241	0.02241	-0.61491	-0.61491
7_2304197_2304883	7	2304197	2304883	1	EHH test	0.03957	0.03957	1.27668	1.27668
8_204204_212607	8	204204	212607	2	CLR test	0.00623	0.00623	-2.09786	-2.09786
8_483433_489835	8	483433	489835	5	CLR test	0.00787	0.00808	-2.14405	-2.19604
8_647568_655971	8	647568	655971	1	CLR test	0.01530	0.01530	-0.45440	-0.45440
8_769668_800090	8	769668	800090	13	CLR test	0.00265	0.00176	-2.12723	-2.18014
8_926034_926241	8	926034	926241	0	EHH test	NA	NA	NA	NA
8_1090593_1090668	8	1090593	1090668	1	EHH test	0.02302	0.02302	1.00751	1.00751
8_1091029_1091214	8	1091029	1091214	1	EHH test	0.02302	0.02302	1.00751	1.00751
8_1094418_1094748	8	1094418	1094748	1	EHH test	0.02477	0.02477	0.24958	0.24958
8_1418998_1446566	8	1418998	1446566	10	EHH test	0.00893	0.00851	-0.91120	-1.47495
8_1424205_1436612	8	1424205	1436612	4	CLR test	0.00639	0.00358	-1.55431	-1.75866
8_1631375_1635775	8	1631375	1635775	5	CLR test	0.00353	0.00301	-1.94614	-1.99007
8_1740142_1740174	8	1740142	1740174	0	EHH test	NA	NA	NA	NA
8_2208204_2208235	8	2208204	2208235	1	EHH test	0.02931	0.02931	1.29305	1.29305
8_2209078_2209161	8	2209078	2209161	1	EHH test	0.02931	0.02931	1.29305	1.29305
9_583922_583969	9	583922	583969	1	EHH test	0.03344	0.03344	0.88654	0.88654
9_683864_683928	9	683864	683928	0	EHH test	NA	NA	NA	NA
9_717086_717124	9	717086	717124	1	EHH test	0.02260	0.02260	0.36425	0.36425
9_724213_789275	9	724213	789275	27	EHH test	0.02125	0.02094	0.77354	0.78677
9_841958_844347	9	841958	844347	3	EHH test	0.01719	0.01251	0.70761	0.76441
9_905253_988356	9	905253	988356	18	EHH test	0.01257	0.00979	-0.40333	-0.27881

9_910944_916344	9	910944	916344	3	CLR test	0.00436	0.00436	-1.87971	-1.87971
9_992663_993672	9	992663	993672	1	EHH test	0.01906	0.01906	0.73422	0.73422
9_1864346_1866043	9	1864346	1866043	2	EHH test	0.03754	0.03754	0.98359	0.98359
9_1888324_1900391	9	1888324	1900391	0	EHH test	NA	NA	NA	NA
10_204642_204726	10	204642	204726	1	EHH test	0.02514	0.02514	2.27899	2.27899
10_338094_338199	10	338094	338199	1	EHH test	0.02464	0.02464	1.00705	1.00705
10_721030_729433	10	721030	729433	4	CLR test	0.00428	0.00284	-2.24301	-2.52555
10_779305_779439	10	779305	779439	1	EHH test	0.02388	0.02388	1.10856	1.10856
10_779442_779789	10	779442	779789	1	EHH test	0.02388	0.02388	1.10856	1.10856
10_1222124_1222373	10	1222124	1222373	1	EHH test	0.05073	0.05073	1.87138	1.87138
11_657275_660132	11	657275	660132	2	EHH test	0.02378	0.02378	0.09588	0.09588
11_711767_712383	11	711767	712383	1	EHH test	0.01324	0.01324	1.39394	1.39394
11_713354_713702	11	713354	713702	0	EHH test	NA	NA	NA	NA
11_730439_771371	11	730439	771371	12	EHH test	0.01065	0.01402	0.52855	0.31863
11_821966_851993	11	821966	851993	15	EHH test	0.01857	0.01997	0.69302	0.66795
11_918019_923421	11	918019	923421	4	CLR test	0.00173	0.00173	-1.57726	-1.57726
11_988629_988675	11	988629	988675	1	EHH test	0.02380	0.02380	0.16635	0.16635
11_1524417_1524610	11	1524417	1524610	1	EHH test	0.02393	0.02393	1.38359	1.38359
11_1584185_1584405	11	1584185	1584405	1	EHH test	0.03834	0.03834	1.92618	1.92618
12_255749_255842	12	255749	255842	1	EHH test	0.02504	0.02504	0.77010	0.77010
12_375891_379043	12	375891	379043	3	EHH test	0.01207	0.01132	-0.52038	-0.74066
12_390210_390427	12	390210	390427	1	EHH test	0.02379	0.02379	0.03078	0.03078
12_391795_439304	12	391795	439304	11	EHH test	0.01270	0.01170	0.59560	0.70052
12_779810_780431	12	779810	780431	1	EHH test	0.02656	0.02656	0.63652	0.63652
12_781040_782045	12	781040	782045	1	EHH test	0.02656	0.02656	0.63652	0.63652
12_995666_1000066	12	995666	1000066	1	CLR test	0.00061	0.00061	-2.42360	-2.42360
13_557462_557580	13	557462	557580	1	EHH test	0.03286	0.03286	1.05649	1.05649
13_558039_599698	13	558039	599698	21	EHH test	0.02071	0.02115	0.02711	-0.38267
13_841220_846622	13	841220	846622	2	CLR test	0.02097	0.02097	-0.23162	-0.23162

Table S6: Summary statistics of the selective sweep regions identified in the *Zymoseptoria tritici* population sampled in Oregon.
Selective sweep regions detected using the composite likelihood ratio (CLR) test and the extended haplotype homozygosity (EHH) test are shown.
Mean and median values of gene-wise nucleotide diversity per site (π) and Tajima's D in selective sweep regions are shown.

Sweep region ID	Chromosome	Start position	Stop position	Number of genes	Scan	Mean value of per gene nucleotide diversity per site (π)	Median value of per gene nucleotide diversity per site (π)	Mean value of per gene Tajima's D	Median value of per gene Tajima's D
1_278958_279122	1	278958	279122	1	Haplotype	0.02604	0.02604	0.78103	0.78103
1_279129_279241	1	279129	279241	1	Haplotype	0.02604	0.02604	0.78103	0.78103
1_1140447_1140543	1	1140447	1140543	0	Haplotype	NA	NA	NA	NA
1_1147908_1162311	1	1147908	1162311	4	SFS	0.00274	0.00260	-1.52383	-1.24436
1_1867074_1951031	1	1867074	1951031	40	Haplotype	0.00811	0.00712	1.22649	1.32574
1_2176434_2190657	1	2176434	2190657	4	Haplotype	0.00683	0.00644	1.28891	1.51834
1_2924557_2924726	1	2924557	2924726	1	Haplotype	0.00450	0.00450	0.41006	0.41006
1_3075071_3075127	1	3075071	3075127	1	Haplotype	0.01704	0.01704	1.68725	1.68725
1_3075139_3075165	1	3075139	3075165	1	Haplotype	0.01704	0.01704	1.68725	1.68725
1_3521613_3526013	1	3521613	3526013	4	SFS	0.00324	0.00291	-2.00058	-2.16860
1_4142274_4164273	1	4142274	4164273	8	Haplotype	0.00622	0.00413	0.96266	1.33593
1_4566275_4573661	1	4566275	4573661	3	Haplotype	0.01280	0.00902	1.54224	1.62685
1_4736038_4768240	1	4736038	4768240	16	Haplotype	0.01418	0.01217	1.76033	1.73222
1_5896807_5896888	1	5896807	5896888	1	Haplotype	0.01750	0.01750	2.12394	2.12394
2_316244_327724	2	316244	327724	6	Haplotype	0.00980	0.00648	0.09875	-1.15648
2_381541_381839	2	381541	381839	1	Haplotype	0.01313	0.01313	1.85912	1.85912
2_409282_410426	2	409282	410426	2	Haplotype	0.02009	0.02009	0.68728	0.68728
2_549898_565891	2	549898	565891	8	Haplotype	0.01010	0.01015	0.84856	0.92170
2_567368_567962	2	567368	567962	1	Haplotype	0.01898	0.01898	1.81076	1.81076
2_572964_573036	2	572964	573036	1	Haplotype	0.00385	0.00385	-1.20592	-1.20592
2_589326_589639	2	589326	589639	1	Haplotype	0.00242	0.00242	0.48211	0.48211
2_590365_590464	2	590365	590464	1	Haplotype	0.01536	0.01536	1.75728	1.75728
2_594389_608795	2	594389	608795	4	SFS	0.00580	0.00543	0.52610	0.50517
2_615586_615694	2	615586	615694	1	Haplotype	0.00285	0.00285	2.39942	2.39942
2_909987_915098	2	909987	915098	1	Haplotype	NA	NA	NA	NA
2_915499_946678	2	915499	946678	11	Haplotype	0.02367	0.02367	3.35875	3.35875
2_1148636_1177050	2	1148636	1177050	8	Haplotype	0.00236	0.00217	-1.92795	-1.66838
2_1176857_1178511	2	1176857	1178511	1	Haplotype	0.00151	0.00151	-1.66838	-1.66838
2_1300453_1301063	2	1300453	1301063	1	Haplotype	NA	NA	NA	NA
2_1303762_1309163	2	1303762	1309163	1	SFS	0.00657	0.00657	1.95904	1.95904
2_1324773_1331174	2	1324773	1331174	3	SFS	0.00316	0.00316	-0.92498	-0.92498
2_1344784_1355187	2	1344784	1355187	3	SFS	0.00963	0.00727	1.22114	0.38895
2_1431830_1441232	2	1431830	1441232	5	SFS	0.02094	0.02094	2.89662	2.89662
2_3322439_3344391	2	3322439	3344391	10	Haplotype	0.00510	0.00484	0.61892	0.46217
2_3707177_3707657	2	3707177	3707657	1	Haplotype	0.00242	0.00242	0.40896	0.40896
2_3707999_3712204	2	3707999	3712204	4	Haplotype	0.00431	0.00431	0.01195	0.01195
3_634526_678883	3	634526	678883	18	Haplotype	0.00794	0.00500	1.23410	0.79753
3_876433_888247	3	876433	888247	6	Haplotype	0.01385	0.01319	1.35070	1.79370
3_1286240_1297833	3	1286240	1297833	6	Haplotype	0.01162	0.01159	1.52845	1.01411
3_1314071_1341912	3	1314071	1341912	15	Haplotype	0.01089	0.00822	1.65204	2.12045
3_1572858_1585260	3	1572858	1585260	1	SFS	NA	NA	NA	NA
3_1631875_1659282	3	1631875	1659282	13	SFS	0.00108	0.00105	-2.12543	-2.29451
3_2617116_2618448	3	2617116	2618448	1	Haplotype	0.01199	0.01199	1.91533	1.91533
3_2870507_2879825	3	2870507	2879825	4	Haplotype	0.01782	0.01645	0.78921	1.31138
3_3117866_3117941	3	3117866	3117941	1	Haplotype	0.03482	0.03482	1.67904	1.67904
4_468486_475491	4	468486	475491	4	Haplotype	0.01794	0.01856	1.64595	1.58687
4_475653_477630	4	475653	477630	1	Haplotype	0.01303	0.01303	1.57875	1.57875
4_1381622_1382190	4	1381622	1382190	1	Haplotype	0.01331	0.01331	0.57073	0.57073
4_2280269_2285566	4	2280269	2285566	2	Haplotype	0.02379	0.02379	2.15366	2.15366
4_2347385_2355734	4	2347385	2355734	3	Haplotype	0.01358	0.01133	2.05710	1.69813
4_2473035_2473608	4	2473035	2473608	1	Haplotype	0.02110	0.02110	1.86579	1.86579
4_2556363_2556629	4	2556363	2556629	1	Haplotype	0.01549	0.01549	0.47699	0.47699
5_878771_879002	5	878771	879002	1	Haplotype	0.00875	0.00875	0.73142	0.73142
5_1203426_1217828	5	1203426	1217828	7	Haplotype	0.00802	0.00503	1.07203	1.40872
5_1275417_1330145	5	1275417	1330145	22	Haplotype	0.00397	0.00139	-0.29691	-0.41084
5_1438812_1443268	5	1438812	1443268	2	Haplotype	0.00783	0.00783	1.87377	1.87377
5_2554069_2562856	5	2554069	2562856	5	Haplotype	0.01042	0.00871	0.14616	0.24493
5_2580542_2580626	5	2580542	2580626	1	Haplotype	0.00290	0.00290	-1.24458	-1.24458
5_2580658_2581065	5	2580658	2581065	1	Haplotype	0.00290	0.00290	-1.24458	-1.24458
5_2582683_2583683	5	2582683	2583683	1	Haplotype	0.00260	0.00260	-1.48166	-1.48166
5_2584238_2585969	5	2584238	2585969	1	Haplotype	0.00698	0.00698	-0.01771	-0.01771
5_2594699_2594937	5	2594699	2594937	1	Haplotype	0.00402	0.00402	-1.97379	-1.97379
5_2614706_2615227	5	2614706	2615227	0	Haplotype	NA	NA	NA	NA
5_2621985_2622360	5	2621985	2622360	1	Haplotype	0.01174	0.01174	1.34660	1.34660
5_2622752_2623057	5	2622752	2623057	0	Haplotype	NA	NA	NA	NA
5_2640530_2669775	5	2640530	2669775	8	Haplotype	0.01344	0.01156	1.42642	1.24959
5_2776881_2776905	5	2776881	2776905	1	Haplotype	0.02203	0.02203	1.55518	1.55518
6_65386_100810	6	65386	100810	11	SFS	0.00388	0.00371	-2.08692	-2.07878
6_253144_254583	6	253144	254583	1	Haplotype	0.01442	0.01442	2.58984	2.58984
6_1234516_1253317	6	1234516	1253317	6	Haplotype	0.00355	0.00219	0.92075	0.76935
6_1542284_1543002	6	1542284	1543002	1	Haplotype	0.01354	0.01354	2.14010	2.14010
6_1708356_1730474	6	1708356	1730474	7	Haplotype	0.00516	0.00278	-0.62545	-0.52506
6_1780168_1781704	6	1780168	1781704	1	Haplotype	0.01970	0.01970	1.24468	1.24468
6_1880261_1880451	6	1880261	1880451	1	Haplotype	0.02042	0.02042	2.21404	2.21404
6_2297945_2298017	6	2297945	2298017	1	Haplotype	0.01073	0.01073	0.00382	0.00382
6_2492343_2492403	6	2492343	2492403	1	Haplotype	0.04943	0.04943	2.42634	2.42634
7_39455_39539	7	39455	39539	1	Haplotype	0.01971	0.01971	1.39922	1.39922
7_232675_250247	7	232675	250247	9	Haplotype	0.00726	0.00683	0.71115	0.91521
7_266745_285266	7	266745	285266	8	Haplotype	0.00745	0.00917	0.82173	0.39378
7_284735_301199	7	284735	301199	8	Haplotype	0.01093	0.01006	1.33207	2.41462
7_457233_475503	7	457233	475503	9	Haplotype	0.01643	0.00842	0.88000	0.83740
7_970719_970758	7	970719	970758	0	Haplotype	NA	NA	NA	NA
7_1026727_1026781	7	1026727	1026781	1	Haplotype	0.01450	0.01450	1.26140	1.26140
7_1388643_1399619	7	1388643	1399619	3	Haplotype	0.00150	0.00150	-2.31132	-2.31132
7_1396541_1400941	7	1396541	1400941	2	SFS	0.00150	0.00150	-2.31132	-2.31132
7_1654916_1728701	7	1654916	1728701	16	Haplotype	0.01322	0.00836	2.60113	3.00028
7_1922301_1939035	7	1922301	1939035	10	Haplotype	0.01535	0.01468	0.12181	0.20603
7_2078869_2078947	7	2078869	2078947	1	Haplotype	0.03307	0.03307	1.83368	1.83368
7_2078945_2081482	7	2078945	2081482	2	Haplotype	0.02654	0.02654	1.78038	1.78038
7_2201462_2212497	7	2201462	2212497	7	Haplotype	0.01713	0.01615	0.81318	0.87598
7_2406927_2418394	7	2406927	2418394	5	Haplotype	0.02381	0.02053	1.02710	0.87922
8_197887_202843	8	197887	202843	4	Haplotype	0.01964	0.01964	1.55392	1.55392
8_685247_685443	8	685247	685443	1	Haplotype	0.02722	0.02722	1.81366	1.81366
8_1127136_1147604	8	1127136	1147604	11	Haplotype	0.01043	0.01216	1.47962	1.59729
8_1717345_1737219	8	1717345	1737219	5	Haplotype	0.01852	0.01852	2.31560	2.31560
8_1910607_1922013	8	1910607	1922013	6	SFS	0.00451	0.00418	-2.19921	-2.16893
8_2062690_2062879	8	2062690	2062879	1	Haplotype	0.03367	0.03367	3.14358	3.14358
9_520165_525390	9	520165	525390	2	Haplotype	0.01266	0.01266	0.49771	0.49771
9_629835_645896	9	629835	645896	6	Haplotype	0.00973	0.00380	0.14409	0.82845
9_668514_686046	9	668514	686046	4	Haplotype	0.01438	0.01659	0.44333	0.92923
9_943974_944295	9	943974	944295	0	Haplotype	NA	NA	NA	NA
9_945481_987912	9	945481	987912	7	Haplotype	0.02124	0.01963	2.12509	1.99612
9_1379254_1383654	9	1379254	1383654	1	SFS	0.00183	0.00183	-2.70234	-2.70234
9_1497804_1509295	9	1497804	1509295	3	Haplotype	0.01275	0.01275	1.26860	1.26860
9_1876909_1879161	9	1876909	1879161	2	Haplotype	0.03168	0.03168	2.45361	2.45361
10_792683_792761	10	792683	792761	1	Haplotype	0.02020	0.02020	0.92154	0.92154
10_792754_792858	10	792754	792858	1	Haplotype	0.02020	0.02020	0.92154	0.92154
10_1135116_1152283	10	1135116	1152283	4	Haplotype	0.02312	0.02312	1.44741	1.44741
10_1186022_1186080	10	1186022	1186080	1	Haplotype	0.02765	0.02765	1.65880	1.65880
10_1257646_1272983	10	1257646	1272983	2	Haplotype	0.01960	0.01960	1.03272	1.03272
10_1272745_1273270	10	1272745	1273270	1	Haplotype	0.03322	0.03322	2.15833	2.15833
10_1274615_1274939	10	1274615	1274939	1	Haplotype	0.03428	0.03428	2.69339	2.69339
10_1348438_1365930	10	1348438	1365930	4	Haplotype	0.01223	0.01117	0.14917	0.16604
10_1483383_1483456	10	1483383	1483456	1	Haplotype	0.02223	0.02223	1.87858	1.87858
10_1488866_1489139	10	1488866	1489139	1	Haplotype	0.02019	0.02019	1.89540	1.89540
11_269032_275468	11	269032	275468	3	Haplotype	0.01388	0.01554	1.73183	1.92979
11_355465_355564	11	355465	355564	1	Haplotype	0.02075	0.02075	2.68321	2.68321
11_355591_355726	11	355591	355726	1	Haplotype	0.02075	0.02075	2.68321	2.68321
11_892383_936330	11	892383	936330	18	Haplotype	0.00806	0.00645	0.63911	1.35727
11_962579_962791	11	962579	962791	0	Haplotype	NA	NA	NA	NA
11_1178840_1179759	11	1178840	1179759	2	Haplotype	0.01571	0.01571	0.49831	0.49831
12_214779_246705	12	214779	246705	11	Haplotype	0.01426	0.01537	1.40518	1.30705
12_247726_251136	12	247726	251136	2	Haplotype	0.03051	0.03051	2.25474	2.25474

12_404932_405079	12	404932	405079	1	Haplotype	0.01582	0.01582	0.20657	0.20657
12_802409_839184	12	802409	839184	16	Haplotype	0.01495	0.01482	1.76284	2.48922
13_373541_385148	13	373541	385148	4	Haplotype	0.01793	0.02066	1.51664	1.52222
13_386267_386514	13	386267	386514	1	Haplotype	0.03465	0.03465	2.69893	2.69893
13_1032179_1055611	13	1032179	1055611	1	SFS	0.00034	0.00034	-1.75995	-1.75995
13_1067239_1089670	13	1067239	1089670	3	SFS	0.00179	0.00127	-1.94211	-2.36500

Table S7: List of the outlier SNPs detected using the population divergence scan among among the four allopatric *Zymoseptoria tritici* populations.
For each outlier SNP, the SNP location, XtX statistic's values, alternative allele counts (AC_ALT) in the four *Z. tritici* populations and pairwise Fst values are shown (mean value in a 1 kb window containing the SNP).

SNP_ID	M_XtX	AC_ALT Australia	AC_ALT Switzerland	AC_ALT Israel	AC_ALT Oregon	Fst Switzerland vs Israel	Fst Switzerland vs Oregon	Fst Israel vs Oregon
chr_1_2948875	16.938	0	0	24	1	NA	NA	NA
chr_1_2949090	16.819	0	0	24	1	NA	NA	NA
chr_1_2949096	16.781	0	0	24	1	NA	NA	NA
chr_1_2949255	16.847	0	0	24	1	NA	NA	NA
chr_1_2949270	16.783	0	0	24	1	NA	NA	NA
chr_1_2949456	16.800	0	0	24	1	NA	NA	NA
chr_1_2949995	16.792	0	0	24	1	NA	NA	NA
chr_1_2950364	16.69032868	0	0	24	1	0.52503	NA	NA
chr_1_2950651	18.29680418	0	0	24	0	0.52503	NA	NA
chr_1_3782543	17.51169984	0	0	5	45	NA	NA	NA
chr_2_1243139	17.0157152	0	0	1	44	-0.02461	0.45520	0.48209
chr_2_1427116	17.94562173	26	0	22	0	NA	NA	NA
chr_2_1427119	17.89323608	26	0	22	0	NA	NA	NA
chr_2_1446193	16.95881687	0	0	0	41	0.02902	0.21743	0.33421
chr_2_1453875	17.82894005	0	0	0	42	0.09092	NA	NA
chr_2_1484243	18.22976665	0	0	24	0	0.31226	NA	0.41845
chr_2_1778133	17.28055272	15	5	24	46	0.22659	0.16457	0.26995
chr_2_1778197	18.18944399	0	0	24	0	0.22659	0.16457	0.26995
chr_2_1778207	18.32626952	0	0	24	0	0.22659	0.16457	0.26995
chr_2_1778410	18.99936594	26	5	24	46	0.22659	0.16457	0.26995
chr_2_1778411	18.61413582	26	5	24	46	0.22659	0.16457	0.26995
chr_2_2367738	18.20396603	0	0	24	0	0.50140	0.05960	NA
chr_2_2367810	18.00735699	0	0	24	0	0.50140	0.05960	NA
chr_2_2367827	18.07183978	0	0	24	0	0.50140	0.05960	NA
chr_2_2367864	18.10028608	0	0	24	0	0.50140	0.05960	NA
chr_3_777547	21.20056874	26	0	3	46	NA	NA	NA
chr_3_777548	21.32752518	26	0	3	46	NA	NA	NA
chr_3_780731	16.90593599	0	23	1	0	NA	NA	NA
chr_3_1231605	19.10183401	0	0	20	40	NA	NA	NA
chr_3_1231636	19.23320517	0	0	20	40	NA	NA	NA
chr_3_1263643	17.246	0	0	13	42	NA	NA	NA
chr_3_1263710	17.067	0	0	13	42	NA	NA	NA
chr_3_1263742	17.19786699	0	0	13	42	NA	NA	NA
chr_3_1263794	17.04335814	0	0	13	42	NA	NA	NA
chr_3_1538017	22.23985451	26	1	22	46	0.16461	0.26207	NA
chr_3_1541055	17.73682659	21	0	11	45	NA	NA	NA
chr_3_1541118	22.46426147	26	0	16	46	NA	NA	NA
chr_3_1541140	22.8213672	26	0	16	46	NA	NA	NA
chr_3_1541142	18.69289388	21	0	15	45	NA	NA	NA
chr_3_1541189	19.91613337	21	0	0	43	NA	NA	NA
chr_3_1541190	19.78108873	21	0	0	43	NA	NA	NA
chr_3_1541202	19.55048137	26	0	1	45	NA	NA	NA
chr_3_1541203	23.23067608	26	0	16	46	NA	NA	NA
chr_3_1541222	22.72891058	26	0	16	46	NA	NA	NA
chr_3_1541224	19.36546566	26	0	1	45	NA	NA	NA
chr_3_1541257	20.07145014	21	0	16	45	NA	NA	NA
chr_3_1545072	24.92647885	21	0	24	43	0.22595	NA	NA
chr_3_1552148	18.48874036	15	0	0	44	0.15552	0.50104	0.46863
chr_3_1644351	16.79201853	0	0	0	40	NA	NA	NA
chr_3_2083875	17.0609717	0	26	0	40	0.34904	NA	NA
chr_3_2083902	16.87058787	0	26	0	40	0.34904	NA	NA
chr_3_2085663	16.97736769	26	1	24	6	NA	NA	NA
chr_3_2086102	16.72271156	26	1	24	6	0.44196	NA	0.39178
chr_3_2086109	16.85996693	26	1	24	6	0.44196	NA	0.39178
chr_3_2086191	16.795	26	1	24	6	0.44196	NA	0.39178
chr_3_2086728	19.949	26	0	24	6	0.44196	NA	0.39178
chr_3_2086744	20.240	26	0	24	6	0.44196	NA	0.39178
chr_3_2086762	19.853	26	0	24	6	0.44196	NA	0.39178
chr_3_2087099	19.801	26	0	24	6	NA	NA	0.39448
chr_3_2087180	20.267	26	0	24	6	NA	NA	0.39448
chr_3_2087356	19.930	26	0	24	6	NA	NA	0.39448
chr_3_2087479	19.809	26	0	24	6	NA	NA	0.39448
chr_3_2087758	19.841	26	0	24	6	NA	NA	0.39448
chr_3_2087925	19.397	26	0	24	6	NA	NA	0.39448
chr_3_2088018	20.039	26	0	24	6	0.32435	NA	0.43882
chr_3_2088091	19.962	26	0	24	6	0.32435	NA	0.43882
chr_3_2088554	18.732	26	1	24	0	0.32435	NA	0.43882
chr_3_2088568	18.645	26	1	24	0	0.32435	NA	0.43882
chr_3_2088623	18.74698701	26	1	24	0	0.32435	NA	0.43882
chr_3_2088695	18.7671832	26	1	24	0	0.32435	NA	0.43882
chr_3_2088807	21.31259314	26	0	24	0	0.32435	NA	0.43882
chr_3_2188464	18.10084895	0	3	22	46	0.52045	0.54454	0.00431
chr_3_2188506	16.73924561	26	21	0	0	0.52045	0.54454	0.00431
chr_3_2188535	19.07968827	26	5	24	46	0.52045	0.54454	0.00431
chr_3_2188556	21.49867256	25	2	23	46	0.52045	0.54454	0.00431
chr_3_2188563	21.21990694	25	2	23	46	0.52045	0.54454	0.00431
chr_3_2188569	21.52632967	25	2	23	46	0.52045	0.54454	0.00431
chr_3_2188574	21.57821324	25	2	23	46	0.52045	0.54454	0.00431
chr_3_2236613	18.31190232	0	0	24	0	NA	NA	NA
chr_3_2320578	18.27698101	0	0	24	0	0.44160	0.12335	0.48110
chr_3_2320596	18.22295943	0	0	24	0	0.44160	0.12335	0.48110
chr_3_2320609	18.33542842	0	0	24	0	0.44160	0.12335	0.48110
chr_3_2320647	18.34557928	0	0	24	0	0.44160	0.12335	0.48110
chr_4_224968	16.91238292	0	0	0	41	0.02530	0.13866	0.20710
chr_4_320351	16.69966875	0	0	0	41	NA	NA	NA
chr_4_326883	16.92354285	0	0	0	41	NA	NA	NA
chr_4_326884	17.28127941	0	0	0	41	NA	NA	NA
chr_4_327731	17.08950257	0	0	0	41	NA	NA	NA
chr_4_1004185	17.57508665	26	4	23	46	0.57706	0.66691	0.09947
chr_4_1004261	19.51111695	26	3	23	46	0.57706	0.66691	0.09947

chr_4_1004262	19.82847427	26	3	23	46	0.57706	0.66691	0.09947
chr_4_1004268	19.67575176	26	3	23	46	0.57706	0.66691	0.09947
chr_4_1004274	19.21229664	26	3	23	46	0.57706	0.66691	0.09947
chr_4_1004457	19.77683422	26	3	23	46	0.57706	0.66691	0.09947
chr_4_1004468	19.74934556	26	3	23	46	0.57706	0.66691	0.09947
chr_4_1004471	19.338	26	3	23	46	0.57706	0.66691	0.09947
chr_4_1004482	19.652	26	3	23	46	0.57706	0.66691	0.09947
chr_4_1004526	19.312	26	3	23	46	0.57706	0.66691	0.09947
chr_4_1004527	19.849	26	3	23	46	0.57706	0.66691	0.09947
chr_4_1004552	19.546	26	3	23	46	0.57706	0.66691	0.09947
chr_4_1004581	19.042	26	3	22	46	0.57706	0.66691	0.09947
chr_4_1004584	19.157	26	3	22	46	0.57706	0.66691	0.09947
chr_4_1004619	17.251	26	1	23	33	0.57706	0.66691	0.09947
chr_4_1004651	18.896	26	3	23	46	0.57706	0.66691	0.09947
chr_4_1004768	21.882	26	3	24	46	0.57706	0.66691	0.09947
chr_4_1004833	21.671	26	3	24	46	0.57706	0.66691	0.09947
chr_4_1004837	19.830	26	4	24	46	0.57706	0.66691	0.09947
chr_4_1004841	19.137	26	5	24	46	0.57706	0.66691	0.09947
chr_4_1004842	21.93085459	26	3	24	46	0.57706	0.66691	0.09947
chr_4_1004912	16.77394147	26	5	23	46	0.57706	0.66691	0.09947
chr_4_1004947	20.34254876	26	4	24	46	0.57706	0.66691	0.09947
chr_4_1004953	21.85524694	26	3	24	46	0.57706	0.66691	0.09947
chr_4_1094925	17.90583194	0	0	20	0	0.21559	0.14837	0.26102
chr_4_1094933	17.52933237	0	0	20	0	0.21559	0.14837	0.26102
chr_4_1094935	17.61993978	0	0	19	0	0.21559	0.14837	0.26102
chr_4_1094939	17.54196975	0	0	19	0	0.21559	0.14837	0.26102
chr_4_1094940	17.56609633	0	0	19	0	0.21559	0.14837	0.26102
chr_4_1094945	17.67173666	0	0	20	0	0.21559	0.14837	0.26102
chr_4_1094954	17.90640139	0	0	20	0	0.21559	0.14837	0.26102
chr_4_1115321	17.97026774	0	0	24	0	NA	NA	NA
chr_4_1115327	18.2490908	0	0	24	0	NA	NA	NA
chr_4_1115329	18.21007473	0	0	24	0	NA	NA	NA
chr_4_1115355	17.9763779	0	0	24	0	NA	NA	NA
chr_4_1115358	18.28494539	0	0	24	0	NA	NA	NA
chr_4_1115371	18.253	0	0	24	0	NA	NA	NA
chr_4_1115375	18.349	0	0	24	0	NA	NA	NA
chr_4_1115389	18.276	0	0	24	0	NA	NA	NA
chr_4_1115422	18.189	0	0	24	0	NA	NA	NA
chr_4_1115424	18.207	0	0	24	0	NA	NA	NA
chr_4_1115454	18.043	0	0	24	0	NA	NA	NA
chr_4_1115455	18.095	0	0	24	0	NA	NA	NA
chr_4_1115459	17.923	0	0	24	0	NA	NA	NA
chr_4_1115463	18.258	0	0	24	0	NA	NA	NA
chr_4_1115464	18.100	0	0	24	0	NA	NA	NA
chr_4_1115468	18.064	0	0	24	0	NA	NA	NA
chr_4_1115481	18.262	0	0	24	0	NA	NA	NA
chr_4_1115596	17.913	0	0	22	0	NA	NA	NA
chr_4_1115640	18.251	0	0	24	0	NA	NA	NA
chr_4_1115677	18.009	0	0	24	0	NA	NA	NA
chr_4_1116305	16.780	0	0	24	2	NA	NA	NA
chr_4_1116308	17.062	0	0	24	2	NA	NA	NA
chr_4_1116311	16.999	0	0	24	2	NA	NA	NA
chr_4_1116318	17.037	0	0	24	2	NA	NA	NA
chr_4_1116619	16.817	0	0	23	3	NA	NA	NA
chr_4_1116625	16.71324844	0	0	23	3	NA	NA	NA
chr_4_1116680	16.69154177	0	0	23	3	NA	NA	NA
chr_4_1116693	16.8824948	0	0	23	3	NA	NA	NA
chr_4_1914116	23.12066391	26	0	0	46	0.02062	0.50522	0.55839
chr_4_1914121	23.79648146	26	0	0	46	0.02062	0.50522	0.55839
chr_4_1914132	20.25415174	26	1	0	46	0.02062	0.50522	0.55839
chr_4_1914223	23.64139797	25	0	0	46	0.02062	0.50522	0.55839
chr_4_1914306	23.18045626	16	0	0	46	0.02062	0.50522	0.55839
chr_5_69206	20.75348992	0	0	14	42	0.32577	0.36134	0.19581
chr_5_85929	17.24040053	0	0	0	41	-0.00700	0.28014	NA
chr_5_125841	18.23989582	0	0	24	0	0.40211	NA	NA
chr_5_125886	18.13030749	0	0	24	0	0.40211	NA	NA
chr_5_127720	18.28989749	0	0	24	0	0.12648	NA	0.24766
chr_5_128672	18.21479211	0	0	24	0	NA	NA	NA
chr_5_128723	18.021	0	0	24	0	NA	NA	NA
chr_5_128725	18.053	0	0	24	0	NA	NA	NA
chr_5_128840	18.229	0	0	24	0	NA	NA	NA
chr_5_128843	18.402	0	0	24	0	NA	NA	NA
chr_5_128865	18.405	0	0	24	0	NA	NA	NA
chr_5_128909	18.158	0	0	24	0	NA	NA	NA
chr_5_128924	18.024	0	0	24	0	NA	NA	NA
chr_5_128963	18.182	0	0	24	0	NA	NA	NA
chr_5_128969	18.045	0	0	24	0	NA	NA	NA
chr_5_128987	18.383	0	0	24	0	NA	NA	NA
chr_5_129011	18.006	0	0	24	0	0.82480	NA	0.82303
chr_5_129020	18.418	0	0	24	0	0.82480	NA	0.82303
chr_5_129101	18.074	0	0	24	0	0.82480	NA	0.82303
chr_5_129107	18.072	0	0	24	0	0.82480	NA	0.82303
chr_5_129113	18.184	0	0	24	0	0.82480	NA	0.82303
chr_5_129170	18.278	0	0	24	0	0.82480	NA	0.82303
chr_5_129179	18.030	0	0	24	0	0.82480	NA	0.82303
chr_5_129200	18.067	0	0	24	0	0.82480	NA	0.82303
chr_5_129233	18.117	0	0	24	0	0.82480	NA	0.82303
chr_5_129278	18.153	0	0	24	0	0.82480	NA	0.82303
chr_5_129308	18.251	0	0	24	0	0.82480	NA	0.82303
chr_5_129318	18.084	0	0	24	0	0.82480	NA	0.82303
chr_5_129405	18.238	0	0	24	0	0.82480	NA	0.82303
chr_5_129428	18.247	0	0	24	0	0.82480	NA	0.82303
chr_5_129447	18.200	0	0	24	0	0.82480	NA	0.82303
chr_5_129467	18.266	0	0	24	0	0.82480	NA	0.82303
chr_5_129546	17.921	0	0	24	0	0.82480	NA	0.82303
chr_5_129560	18.158	0	0	24	0	0.82480	NA	0.82303
chr_5_129566	18.398	0	0	24	0	0.82480	NA	0.82303
chr_5_129569	17.849	0	0	24	0	0.82480	NA	0.82303
chr_5_129573	18.102	0	0	24	0	0.82480	NA	0.82303
chr_5_129578	18.312	0	0	24	0	0.82480	NA	0.82303
chr_5_129596	18.167	0	0	24	0	0.82480	NA	0.82303
chr_5_129615	18.195	0	0	24	0	0.82480	NA	0.82303
chr_5_129642	18.188	0	0	24	0	0.82480	NA	0.82303
chr_5_129661	18.026	0	0	24	0	0.82480	NA	0.82303
chr_5_129670	18.165	0	0	24	0	0.82480	NA	0.82303
chr_5_129700	18.171	0	0	24	0	0.82480	NA	0.82303
chr_5_129701	18.096	0	0	24	0	0.82480	NA	0.82303
chr_5_129716	18.000	0	0	24	0	0.82480	NA	0.82303
chr_5_129842	18.123	0	0	24	0	0.82480	NA	0.82303
chr_5_129849	18.351	0	0	24	0	0.82480	NA	0.82303
chr_5_129907	18.219	0	0	24	0	0.82480	NA	0.82303
chr_5_129908	18.220	0	0	24	0	0.82480	NA	0.82303

chr_5_129931	18.079	0	0	24	0	0.82480	NA	0.82303
chr_5_129955	17.902	0	0	24	0	0.82480	NA	0.82303
chr_5_129961	18.407	0	0	24	0	0.82480	NA	0.82303
chr_5_129972	18.211	0	0	24	0	0.82480	NA	0.82303
chr_5_130020	18.194	0	0	24	0	0.85364	NA	0.86471
chr_5_130024	18.285	0	0	24	0	0.85364	NA	0.86471
chr_5_130046	18.023	0	0	24	0	0.85364	NA	0.86471
chr_5_130053	18.037	0	0	24	0	0.85364	NA	0.86471
chr_5_130055	17.976	0	0	24	0	0.85364	NA	0.86471
chr_5_130089	18.037	0	0	24	0	0.85364	NA	0.86471
chr_5_130120	18.163	0	0	24	0	0.85364	NA	0.86471
chr_5_130151	18.170	0	0	24	0	0.85364	NA	0.86471
chr_5_130183	18.207	0	0	24	0	0.85364	NA	0.86471
chr_5_130233	18.011	0	0	24	0	0.85364	NA	0.86471
chr_5_130238	18.218	0	0	24	0	0.85364	NA	0.86471
chr_5_130278	18.272	0	0	24	0	0.85364	NA	0.86471
chr_5_130298	18.274	0	0	24	0	0.85364	NA	0.86471
chr_5_130336	18.238	0	0	24	0	0.85364	NA	0.86471
chr_5_130338	18.059	0	0	24	0	0.85364	NA	0.86471
chr_5_130421	18.292	0	0	24	0	0.85364	NA	0.86471
chr_5_130442	18.031	0	0	24	0	0.85364	NA	0.86471
chr_5_130445	18.378	0	0	24	0	0.85364	NA	0.86471
chr_5_130451	18.332	0	0	24	0	0.85364	NA	0.86471
chr_5_130466	18.224	0	0	24	0	0.85364	NA	0.86471
chr_5_130473	18.237	0	0	24	0	0.85364	NA	0.86471
chr_5_130524	17.924	0	0	24	0	0.85364	NA	0.86471
chr_5_130538	18.068	0	0	24	0	0.85364	NA	0.86471
chr_5_130547	18.113	0	0	24	0	0.85364	NA	0.86471
chr_5_130571	18.126	0	0	24	0	0.85364	NA	0.86471
chr_5_130579	18.120	0	0	24	0	0.85364	NA	0.86471
chr_5_130613	18.121	0	0	24	0	0.85364	NA	0.86471
chr_5_130616	18.029	0	0	24	0	0.85364	NA	0.86471
chr_5_130622	18.181	0	0	24	0	0.85364	NA	0.86471
chr_5_130637	18.293	0	0	24	0	0.85364	NA	0.86471
chr_5_130641	18.111	0	0	24	0	0.85364	NA	0.86471
chr_5_130644	18.274	0	0	24	0	0.85364	NA	0.86471
chr_5_130648	17.820	0	0	24	0	0.85364	NA	0.86471
chr_5_130652	18.485	0	0	24	0	0.85364	NA	0.86471
chr_5_130655	18.216	0	0	24	0	0.85364	NA	0.86471
chr_5_130656	18.340	0	0	24	0	0.85364	NA	0.86471
chr_5_130669	18.176	0	0	24	0	0.85364	NA	0.86471
chr_5_130683	18.075	0	0	24	0	0.85364	NA	0.86471
chr_5_130696	18.217	0	0	24	0	0.85364	NA	0.86471
chr_5_130698	18.293	0	0	24	0	0.85364	NA	0.86471
chr_5_130712	18.323	0	0	24	0	0.85364	NA	0.86471
chr_5_130729	18.232	0	0	24	0	0.85364	NA	0.86471
chr_5_130735	18.034	0	0	24	0	0.85364	NA	0.86471
chr_5_130779	18.428	0	0	24	0	0.85364	NA	0.86471
chr_5_130788	18.239	0	0	24	0	0.85364	NA	0.86471
chr_5_130804	18.134	0	0	24	0	0.85364	NA	0.86471
chr_5_130805	18.246	0	0	24	0	0.85364	NA	0.86471
chr_5_130806	18.240	0	0	24	0	0.85364	NA	0.86471
chr_5_130810	18.279	0	0	24	0	0.85364	NA	0.86471
chr_5_130812	18.245	0	0	24	0	0.85364	NA	0.86471
chr_5_130838	18.079	0	0	24	0	0.85364	NA	0.86471
chr_5_130869	18.045	0	0	24	0	0.85364	NA	0.86471
chr_5_130890	18.287	0	0	24	0	0.85364	NA	0.86471
chr_5_130935	18.060	0	0	24	0	0.85364	NA	0.86471
chr_5_130943	18.475	0	0	24	0	0.85364	NA	0.86471
chr_5_130986	18.362	0	0	24	0	0.85364	NA	0.86471
chr_5_131013	18.254	0	0	24	0	0.78744	NA	NA
chr_5_131034	18.289	0	0	24	0	0.78744	NA	NA
chr_5_131043	18.277	0	0	24	0	0.78744	NA	NA
chr_5_131049	18.415	0	0	24	0	0.78744	NA	NA
chr_5_131052	18.405	0	0	24	0	0.78744	NA	NA
chr_5_131058	18.308	0	0	24	0	0.78744	NA	NA
chr_5_131067	18.359	0	0	24	0	0.78744	NA	NA
chr_5_131082	18.228	0	0	24	0	0.78744	NA	NA
chr_5_131088	18.127	0	0	24	0	0.78744	NA	NA
chr_5_131103	18.096	0	0	24	0	0.78744	NA	NA
chr_5_131106	18.216	0	0	24	0	0.78744	NA	NA
chr_5_131112	18.016	0	0	24	0	0.78744	NA	NA
chr_5_131121	18.142	0	0	24	0	0.78744	NA	NA
chr_5_131130	18.435	0	0	24	0	0.78744	NA	NA
chr_5_131157	18.304	0	0	24	0	0.78744	NA	NA
chr_5_131243	18.163	0	0	24	0	0.78744	NA	NA
chr_5_131830	18.193	0	0	24	0	0.78744	NA	NA
chr_5_131832	18.424	0	0	24	0	0.78744	NA	NA
chr_5_131844	18.183	0	0	24	0	0.78744	NA	NA
chr_5_131847	18.541	0	0	24	0	0.78744	NA	NA
chr_5_131850	18.258	0	0	24	0	0.78744	NA	NA
chr_5_131852	18.106	0	0	24	0	0.78744	NA	NA
chr_5_131877	18.234	0	0	24	0	0.78744	NA	NA
chr_5_131881	18.021	0	0	23	0	0.78744	NA	NA
chr_5_131887	18.137	0	0	24	0	0.78744	NA	NA
chr_5_131888	18.398	0	0	24	0	0.78744	NA	NA
chr_5_131890	18.132	0	0	24	0	0.78744	NA	NA
chr_5_131898	17.914	0	0	24	0	0.78744	NA	NA
chr_5_131927	18.176	0	0	24	0	0.78744	NA	NA
chr_5_131929	18.197	0	0	24	0	0.78744	NA	NA
chr_5_131932	18.341	0	0	24	0	0.78744	NA	NA
chr_5_132034	18.091	0	0	24	0	NA	NA	0.67991
chr_5_132094	18.041	0	0	24	0	NA	NA	0.67991
chr_5_132133	18.345	0	0	24	0	NA	NA	0.67991
chr_5_132151	18.252	0	0	24	0	NA	NA	0.67991
chr_5_132172	18.284	0	0	24	0	NA	NA	0.67991
chr_5_132190	18.205	0	0	24	0	NA	NA	0.67991
chr_5_132199	18.118	0	0	24	0	NA	NA	0.67991
chr_5_132208	18.117	0	0	24	0	NA	NA	0.67991
chr_5_132373	18.083	0	0	24	0	NA	NA	0.67991
chr_5_132849	17.714	0	0	20	0	NA	NA	0.67991
chr_5_132860	17.769	0	0	20	0	NA	NA	0.67991
chr_5_132864	17.740	0	0	20	0	NA	NA	0.67991
chr_5_132869	17.565	0	0	20	0	NA	NA	0.67991
chr_5_132894	17.657	0	0	20	0	NA	NA	0.67991
chr_5_132901	17.687	0	0	20	0	NA	NA	0.67991
chr_5_132904	17.596	0	0	20	0	NA	NA	0.67991
chr_5_133498	18.154	0	0	24	0	0.33692	NA	0.35117
chr_5_133501	18.362	0	0	24	0	0.33692	NA	0.35117
chr_5_133543	18.20731891	0	0	24	0	0.33692	NA	0.35117
chr_5_133605	18.3825721	0	0	24	0	0.33692	NA	0.35117
chr_5_272962	18.36647727	0	0	24	0	0.26730	0.02716	0.33411
chr_5_517240	17.80433371	0	0	23	0	0.28733	0.41743	0.35013
chr_5_517241	17.9926775	0	0	23	0	0.28733	0.41743	0.35013
chr_5_517261	18.05225374	0	0	23	0	0.28733	0.41743	0.35013
chr_5_517262	16.70753416	0	20	0	0	0.28733	0.41743	0.35013

chr_5_2695259	17.07070046	26	1	24	12	NA	0.02610	0.07461
chr_6_651670	18.1509385	0	0	0	43	0.19145	0.37205	0.16669
chr_6_680360	18.62460736	23	0	0	43	NA	NA	NA
chr_6_681039	17.00024115	0	0	0	41	NA	NA	NA
chr_6_820377	16.92971386	0	0	24	3	0.40032	NA	NA
chr_6_835689	17.58730903	0	0	0	42	0.25351	NA	0.33245
chr_6_978603	16.72184046	0	0	24	2	0.56904	0.01430	NA
chr_6_978686	16.71318471	0	0	24	3	0.56904	0.01430	NA
chr_6_978716	17.10554901	0	0	24	3	0.56904	0.01430	NA
chr_6_978750	16.724	0	0	24	3	0.56904	0.01430	NA
chr_6_978791	16.85361543	0	0	24	3	0.56904	0.01430	NA
chr_6_979258	16.75140801	0	0	24	3	NA	NA	NA
chr_6_1270254	17.88830082	0	0	22	0	0.22907	0.04671	0.34524
chr_7_183285	21.49430341	0	0	0	46	NA	NA	NA
chr_7_183785	22.19701256	0	0	0	46	NA	NA	NA
chr_7_184266	25.00661022	0	1	24	46	NA	NA	NA
chr_7_184376	21.06041386	0	3	24	46	NA	NA	NA
chr_7_406386	18.18311532	0	0	24	0	0.32158	NA	NA
chr_7_1367438	17.51592307	0	21	0	0	NA	NA	NA
chr_7_1367487	16.83838129	0	21	0	0	NA	NA	NA
chr_7_1367513	17.01046941	0	21	0	0	NA	NA	NA
chr_7_1367632	17.05810697	0	21	0	0	NA	NA	NA
chr_7_1367734	17.32164486	0	21	0	0	NA	NA	NA
chr_7_1896337	18.8971217	0	0	0	44	0.08004	0.28998	NA
chr_8_1368207	22.02680166	26	3	24	46	NA	NA	NA
chr_9_881881	17.94731179	26	1	24	0	0.10959	0.04824	0.17401
chr_10_450514	16.909	26	27	0	44	NA	NA	NA
chr_10_452599	16.69962058	26	27	0	44	NA	NA	NA
chr_10_452864	16.84693243	26	27	0	44	NA	NA	NA
chr_10_465112	18.74441352	0	0	0	43	NA	NA	NA
chr_11_814692	17.18537397	26	4	22	46	0.18062	0.28965	NA
chr_11_921528	16.89074615	0	0	24	4	NA	NA	NA
chr_11_922117	18.03857346	0	0	24	0	NA	NA	NA
chr_11_923521	16.99225115	0	0	24	4	NA	NA	0.58860
chr_11_923522	16.9799953	0	0	24	4	NA	NA	0.58860
chr_11_923523	17.05759974	0	0	24	4	NA	NA	0.58860
chr_11_923527	16.72943112	0	0	24	4	NA	NA	0.58860
chr_11_923628	16.98649137	0	0	24	4	NA	NA	0.58860
chr_11_923781	16.93432934	0	0	24	4	NA	NA	0.58860
chr_11_923837	17.06589097	0	0	24	4	NA	NA	0.58860
chr_11_924016	16.94045678	0	0	24	4	0.58134	0.04221	0.58612
chr_11_924017	16.72118454	0	0	24	4	0.58134	0.04221	0.58612
chr_11_924111	16.93342141	0	0	24	4	0.58134	0.04221	0.58612
chr_11_924134	17.16001095	0	0	24	9	0.58134	0.04221	0.58612
chr_11_924148	17.04913064	0	0	24	9	0.58134	0.04221	0.58612
chr_11_924153	17.06285198	0	0	24	9	0.58134	0.04221	0.58612
chr_11_924163	16.85004185	0	0	24	4	0.58134	0.04221	0.58612
chr_11_924176	16.72952159	0	0	24	4	0.58134	0.04221	0.58612
chr_11_924184	17.05853353	0	0	24	4	0.58134	0.04221	0.58612
chr_11_924200	17.21594859	0	0	24	9	0.58134	0.04221	0.58612
chr_11_924201	17.00535796	0	0	24	9	0.58134	0.04221	0.58612
chr_11_924226	17.03146795	0	0	24	4	0.58134	0.04221	0.58612
chr_11_924227	16.69695872	0	0	24	4	0.58134	0.04221	0.58612
chr_11_924252	16.97922073	0	0	24	9	0.58134	0.04221	0.58612
chr_11_924267	16.73962722	0	0	24	4	0.58134	0.04221	0.58612
chr_11_924293	16.92637226	0	0	24	9	0.58134	0.04221	0.58612
chr_11_924296	16.71132366	0	0	24	4	0.58134	0.04221	0.58612
chr_11_924338	17.0530505	0	0	24	4	0.58134	0.04221	0.58612
chr_11_924368	18.2494717	0	0	24	0	0.58134	0.04221	0.58612
chr_11_924384	18.22480107	0	0	24	0	0.58134	0.04221	0.58612
chr_11_924423	18.10586521	0	0	24	0	0.58134	0.04221	0.58612
chr_11_924535	18.21850478	0	0	24	0	0.58134	0.04221	0.58612
chr_11_924938	18.36687713	0	0	24	0	0.58134	0.04221	0.58612
chr_12_63668	19.818	24	0	24	0	0.18056	NA	0.30415
chr_12_63672	20.03263208	24	0	24	0	0.18056	NA	0.30415
chr_12_63673	20.35156903	24	0	24	0	0.18056	NA	0.30415
chr_12_63786	18.16807304	0	0	24	0	0.18056	NA	0.30415
chr_12_63815	17.00837809	3	26	0	46	0.18056	NA	0.30415
chr_12_63873	16.79753077	3	26	0	46	0.18056	NA	0.30415
chr_13_480239	22.29496551	26	0	16	46	0.15889	NA	0.18941
chr_13_1114924	16.99079139	0	0	0	40	NA	0.01390	0.02102

Table S8: List of the outlier SNPs detected in the cross-population extended haplotype homozygosity (XP-EHH) scan among the two Oregon *Zymoseptoria tritici* sympatric populations. For each outlier SNP, the SNP location, XP-EHH statistic's values and p value are shown.

SNP_ID	XP-EHH	pvalue (log10 transformed)
chr_1_1887262	5.2437	6.8030
chr_1_1887278	5.7528	8.0565
chr_1_1887285	5.9301	8.5189
chr_1_1887289	5.7703	8.1016
chr_1_1887340	5.8677	8.3547
chr_1_1887358	5.8421	8.2879
chr_1_1887377	5.7611	8.0781
chr_1_1887411	5.8665	8.3516
chr_1_1887439	6.0593	8.8641
chr_1_1887441	6.0667	8.8841
chr_1_1887520	5.3681	7.0994
chr_1_1887523	5.5322	7.5000
chr_1_1887676	5.3045	6.9470
chr_1_1887874	5.4066	7.1922
chr_1_1887919	5.4527	7.3043
chr_1_1887930	5.4338	7.2583
chr_1_1887940	5.5602	7.5695
chr_1_1887958	5.4951	7.4085
chr_1_1887959	6.1615	9.1424
chr_1_1896683	5.5283	7.4904
chr_1_1896699	5.6204	7.7200
chr_1_1896784	5.3698	7.1033
chr_1_1896915	5.2559	6.8319
chr_1_1896937	5.3064	6.9515
chr_1_1896940	5.5155	7.4587
chr_1_1896941	5.6725	7.8517
chr_1_1896946	5.9290	8.5159
chr_1_1896947	5.5855	7.6326
chr_1_1903585	5.2606	6.8428
chr_1_1903718	5.2273	6.7645
chr_1_1903719	5.2820	6.8935
chr_1_1903723	5.3115	6.9637
chr_1_1904026	5.3692	7.1020
chr_1_1904036	5.4395	7.2721
chr_1_1904051	5.3477	7.0502
chr_1_1904091	5.3887	7.1490
chr_1_1904113	5.6278	7.7386
chr_1_1904118	5.8240	8.2407
chr_1_1904133	5.8451	8.2956
chr_1_1904138	5.8271	8.2488
chr_1_1904155	5.5276	7.4887
chr_1_1904192	5.5242	7.4803
chr_1_1904213	5.6910	7.8986
chr_1_1904220	5.8335	8.2654
chr_1_1904260	5.4882	7.3915
chr_1_1904292	5.7944	8.1639
chr_1_1904313	5.7821	8.1320
chr_1_1904325	5.7331	8.0062
chr_1_1904343	5.5148	7.4570
chr_1_1904422	5.6759	7.8601
chr_1_1904435	5.7945	8.1643
chr_1_1904439	5.8494	8.3068
chr_1_1904450	5.7832	8.1350
chr_1_1904456	5.7477	8.0436
chr_1_1904501	5.7998	8.1780
chr_1_1904503	5.7931	8.1606
chr_1_1904567	5.4553	7.3108
chr_1_1904572	5.4559	7.3122
chr_1_1904597	5.2390	6.7920
chr_1_1904770	5.2830	6.8959
chr_1_1904792	5.2332	6.7783
chr_1_1904847	5.4104	7.2015
chr_1_1904867	5.4792	7.3693
chr_1_1904904	5.4616	7.3262
chr_1_1905149	5.3682	7.0995
chr_1_1905151	5.5211	7.4725
chr_1_1905153	5.5123	7.4509
chr_1_1905170	5.6952	7.9094
chr_1_1905176	5.6258	7.7336
chr_1_1905203	5.5308	7.4966
chr_1_1905221	5.6357	7.7585
chr_1_1905716	5.3387	7.0286
chr_1_1905723	5.3848	7.1396
chr_1_1905727	5.6986	7.9179
chr_1_1921681	5.2339	6.7801
chr_1_1921702	6.0392	8.8101
chr_1_1921719	5.9406	8.5468
chr_1_1921728	5.8449	8.2951
chr_1_1921765	5.8463	8.2988
chr_1_1921812	5.4081	7.1958
chr_1_1921854	5.5079	7.4399
chr_1_1921868	5.4462	7.2885
chr_1_1921924	5.4862	7.3865
chr_1_1921928	5.4225	7.2307
chr_1_1922414	5.2613	6.8445
chr_1_1927341	5.7275	7.9918
chr_1_1927345	6.0289	8.7823
chr_1_1927358	6.4073	9.8293
chr_1_1927362	6.5960	10.3744
chr_1_1927368	6.5231	10.1619
chr_1_1927377	5.6009	7.6712
chr_1_1927489	5.7635	8.0840
chr_1_1927523	5.9325	8.5251
chr_1_1927524	6.0411	8.8152
chr_1_1927540	5.9252	8.5059
chr_1_1927563	6.0041	8.7157
chr_1_1927567	5.9651	8.6118
chr_1_1927571	5.4826	7.3778
chr_1_1927964	5.6980	7.9165
chr_1_1927978	5.6385	7.7657
chr_1_1927979	5.9256	8.5070
chr_1_1927980	5.6142	7.7046
chr_1_1927996	5.2555	6.8310
chr_1_1928120	5.5349	7.5067
chr_1_1928131	5.6428	7.7766
chr_1_1928197	5.8431	8.2903
chr_1_1928218	6.1562	9.1279
chr_1_1928238	6.1641	9.1495
chr_1_1928239	6.2536	9.3968
chr_1_1928337	5.5297	7.4937
chr_1_1928374	5.7549	8.0621

chr_1_1928385	6.0934	8.9565
chr_1_1928399	5.8882	8.4084
chr_1_1928401	5.8690	8.3582
chr_1_1928436	5.5474	7.5376
chr_1_1928644	5.3854	7.1410
chr_1_1928793	5.3502	7.0561
chr_1_1928796	5.3669	7.0963
chr_1_1928809	5.4480	7.2929
chr_1_1928815	5.4051	7.1886
chr_1_1928882	5.2309	6.7730
chr_1_1928885	5.3794	7.1264
chr_1_1928897	5.4189	7.2221
chr_1_1928906	5.4426	7.2797
chr_1_1928979	5.4749	7.3587
chr_1_1928980	5.5414	7.5229
chr_1_1928986	5.5788	7.6158
chr_1_1928989	5.5789	7.6160
chr_1_1928995	5.5372	7.5125
chr_1_1929001	5.4989	7.4177
chr_1_1929014	5.4844	7.3821
chr_1_1929024	5.4527	7.3044
chr_1_1929036	5.4451	7.2859
chr_1_1929053	5.4266	7.2409
chr_1_1929070	5.4859	7.3858
chr_1_1929073	5.5269	7.4869
chr_1_1929075	5.5319	7.4992
chr_1_1929077	5.6134	7.7025
chr_1_1929080	5.4637	7.3314
chr_1_1929102	5.4681	7.3421
chr_1_1929103	5.4680	7.3419
chr_1_1929243	5.3662	7.0948
chr_1_1929244	5.4920	7.4007
chr_1_1929247	5.5446	7.5308
chr_1_1929248	5.5182	7.4655
chr_1_1929259	5.4568	7.3144
chr_1_1929269	5.3814	7.1313
chr_1_2963630	5.2401	6.7946
chr_1_2963644	5.2519	6.8224
chr_1_2963717	5.2639	6.8506
chr_1_2964361	5.2904	6.9134
chr_1_4210591	5.3495	7.0546
chr_1_4210601	5.3296	7.0068
chr_1_4210625	5.2857	6.9023
chr_2_1001593	5.2802	6.8892
chr_2_1001595	5.3960	7.1665
chr_2_1001606	5.3042	6.9462
chr_2_1001618	5.3022	6.9415
chr_2_1001630	5.3319	7.0123
chr_2_1001646	5.2592	6.8395
chr_2_1001698	5.2981	6.9319
chr_2_1001907	5.2274	6.7646
chr_6_841039	-5.5261	7.4850
chr_6_841042	-5.4200	7.2248
chr_6_841089	-5.3375	7.0259
chr_6_841278	-5.4334	7.2574
chr_6_841834	-5.2978	6.9311
chr_6_841898	-5.2649	6.8532
chr_6_841958	-5.3399	7.0316
chr_6_842009	-5.4079	7.1954
chr_6_842017	-5.4451	7.2859
chr_6_842022	-5.3817	7.1321
chr_6_842033	-5.4534	7.3061
chr_6_842050	-5.4421	7.2785
chr_6_842088	-5.4675	7.3407
chr_6_842093	-5.4290	7.2466
chr_6_842126	-5.4199	7.2246
chr_6_842153	-5.4450	7.2855
chr_6_842177	-5.4945	7.4069
chr_6_842180	-5.2849	6.9003
chr_6_842183	-5.2579	6.8366
chr_6_842259	-5.2369	6.7870
chr_6_842320	-5.4291	7.2470
chr_6_842324	-5.4288	7.2463
chr_6_842351	-5.4272	7.2422
chr_6_842359	-5.5199	7.4696
chr_6_842367	-5.4951	7.4083
chr_6_842420	-5.4954	7.4091
chr_6_842450	-5.5074	7.4386
chr_6_842454	-5.4779	7.3661
chr_6_842493	-5.5009	7.4227
chr_6_842549	-5.2428	6.8009
chr_6_842723	-5.2473	6.8115
chr_6_842768	-5.3088	6.9572
chr_6_2492790	-5.4984	7.4165
chr_6_2492796	-5.7396	8.0228
chr_6_2492803	-5.8331	8.2644
chr_6_2492814	-6.3018	9.5312
chr_6_2492820	-6.1725	9.1724
chr_9_49415	5.3149	6.9718
chr_9_50926	5.6068	7.6859
chr_9_50930	5.5997	7.6680
chr_9_50987	6.3105	9.5559
chr_9_51033	5.8510	8.3111
chr_9_51048	5.3803	7.1286
chr_9_51061	5.5735	7.6026
chr_9_51064	5.2913	6.9156
chr_9_51093	5.3390	7.0295
chr_9_51115	5.2350	6.7825
chr_9_51145	5.3752	7.1163
chr_9_51154	5.8192	8.2282
chr_9_51166	6.4939	10.0774
chr_9_51172	5.9593	8.5964
chr_9_51178	5.5688	7.5910
chr_9_51223	5.3943	7.1625
chr_9_51376	5.2846	6.8996
chr_9_51379	5.4445	7.2843
chr_9_51398	5.3323	7.0132
chr_12_74110	5.4734	7.3551
chr_12_74112	5.3615	7.0833

Table S9: Significantly over-represented gene ontology terms linked to biological processes in selective sweep regions of all four allopatric *Zymoseptoria tritici* populations.
Scores are based on negative log-10 transformed p-values obtained from a hyper-geometric test with α < 0.05.

Gene ontology (GO) Term	Enrichment p-value	Odds Ratio	Expected GO term count	Effective GO term count	Total proteins per GO	Term
GO:0006886	0.00504	1.8528	19.956	31	95	intracellular protein transport
GO:0046907	0.00556	1.7635	23.317	35	111	intracellular transport
GO:0051641	0.00579	1.7133	25.838	38	123	cellular localization
GO:0006626	0.00581	9.4511	1.4705	5	7	protein targeting to mitochondrion
GO:0034613	0.00629	1.7782	21.847	33	104	cellular protein localization
GO:0070727	0.00629	1.7782	21.847	33	104	cellular macromolecule localization
GO:0051649	0.00650	1.7400	23.528	35	112	establishment of localization in cell
GO:0015031	0.00775	1.6728	26.258	38	125	protein transport
GO:0008104	0.00894	1.6301	28.149	40	134	protein localization
GO:1902582	0.00914	3.4101	3.9913	9	19	single-organism intracellular transport
GO:0007018	0.00917	4.4155	2.7309	7	13	microtubule-based movement
GO:0072655	0.01283	6.2987	1.6805	5	8	establishment of protein localization to mitochondrion
GO:0070585	0.01283	6.2987	1.6805	5	8	protein localization to mitochondrion
GO:0045184	0.01336	1.5972	27.099	38	129	establishment of protein localization
GO:0007005	0.01358	3.0991	4.2013	9	20	mitochondrion organization
GO:0033036	0.01468	1.5458	30.670	42	146	macromolecule localization
GO:0017038	0.01474	4.5376	2.3107	6	11	protein import
GO:0006839	0.01474	4.5376	2.3107	6	11	mitochondrial transport
GO:0006928	0.01506	3.7835	2.9409	7	14	movement of cell or subcellular component
GO:0072594	0.01550	2.6062	5.6718	11	27	establishment of protein localization to organelle
GO:0006605	0.02080	2.4521	5.8819	11	28	protein targeting
GO:0006810	0.02215	1.2201	159.021	180	757	transport
GO:0051179	0.02257	1.2175	161.962	183	771	localization
GO:0007017	0.02371	2.5250	5.2517	10	25	microtubule-based process
GO:0051234	0.02705	1.2098	159.861	180	761	establishment of localization
GO:0032446	0.03039	2.7514	3.9913	8	19	protein modification by small protein conjugation
GO:0033365	0.03523	2.1926	6.3020	11	30	protein localization to organelle
GO:1902580	0.03623	2.4327	4.8315	9	23	single-organism cellular localization
GO:0010605	0.03931	5.0330	1.4705	4	7	negative regulation of macromolecule metabolic process
GO:0071826	0.03971	3.7768	2.1007	5	10	ribonucleoprotein complex subunit organization

Table S11: List of the genes found in regions highly differentiated among the four allopatric *Zymoseptoria tritici* populations.

Presence of a predicted function in transmembrane transport or secretion is shown. Predicted functions in transmembrane transport or secretion are shown. Nucleotide diversity per site (π) and Tajima's D per gene in the four *Zymoseptoria tritici* populations are shown.

Gene ID	Gene category	Secretion	Protein family (PFAM) domain	π Australia population	π Swiss population	π Israel population	π Oregon population	Tajima's D Australian population	Tajima's D Swiss population	Tajima's D Israel population	Tajima's D Oregon population
Zt09_TU_10_00161	.	Not secreted	Methyltransferase domain, Polyketide synthase dehydratase, Beta-ketoacyl synthase, C-terminal domain, KR domain, Acyl transferase domain, Phosphopantetheine attachment site, Ketoacyl-synthetase C-terminal extension, Beta-ketoacyl synthase, N-terminal domain, Condensation domain	0.00019	0.00051	0.00290	0.00024	-1.4149	-1.9575	0.8068	-2.2329
Zt09_TU_11_00256	.	Not secreted	NA	NA	NA	NA	NA	NA	NA	NA	NA
Zt09_TU_11_00299	.	Not secreted	Protein of unknown function (DUF2370)	NA	NA	0.00161	NA	NA	NA	-0.9755	NA
Zt09_TU_11_00300	.	Not secreted	Probable N6-adenine methyltransferase	NA	NA	NA	0.00524	NA	NA	NA	-0.3684
Zt09_TU_12_00012	.	Secreted	NA	0.00237	0.00264	0.00456	NA	-1.2871	-2.0139	-0.6175	NA
Zt09_TU_1_01013	.	Not secreted	C2 domain, Phosphatidylinositol-specific phospholipase C, Y domain, Phosphatidylinositol-specific phospholipase C, X domain	NA	0.00112	0.00061	0.00056	NA	-2.2316	-2.1694	-2.5376
Zt09_TU_2_00455	.	Not secreted	Bestrophin, RFP-TM, chloride channel	NA	0.00838	0.00628	0.01171	NA	0.6999	0.2622	0.9414
Zt09_TU_2_00463	.	Not secreted	AMP-binding enzyme, Acetyl-coenzyme A synthetase N-terminus	NA	0.00583	0.00615	0.00872	NA	1.4054	2.1166	2.4325
Zt09_TU_2_00479	.	Not secreted	Fungal Zn(2)-Cys(6) binuclear cluster domain, Fungal specific transcription factor domain	NA	0.00172	0.00128	0.02012	NA	-2.0764	-0.7915	2.1411
Zt09_TU_2_00572	Small secreted protein	Secreted	NA	0.00784	0.01641	0.01525	NA	-0.5204	-0.2568	0.2806	NA
Zt09_TU_2_00776	.	Not secreted	GDP/GTP exchange factor Sec2p	NA	0.00820	NA	NA	NA	1.5944	NA	NA
Zt09_TU_3_00230	.	Secreted	NA	NA	NA	0.00347	NA	NA	NA	-1.4870	NA
Zt09_TU_3_00231	Small secreted protein	Secreted	NA	NA	NA	0.00392	NA	NA	NA	-2.0614	NA
Zt09_TU_3_00359	.	Not secreted	Zinc finger, C3HC4 type (RING finger)	NA	0.00112	0.01610	NA	NA	-2.0825	2.9694	NA
Zt09_TU_3_00466	.	Secreted	Deuterolysin metalloprotease (M35) family	NA	NA	0.00383	NA	NA	NA	0.7228	NA
Zt09_TU_3_00467	Small secreted protein	Secreted	NA	NA	NA	0.01385	0.00183	NA	NA	1.4045	-2.1416
Zt09_TU_3_00492	.	Not secreted	Domain of unknown function (DUF1989)	NA	0.00568	0.00258	0.00098	NA	0.3977	-1.6590	-2.4678
Zt09_TU_3_00646	.	Not secreted	NA	NA	0.00090	NA	NA	NA	-2.3176	NA	NA
Zt09_TU_3_00697	.	Not secreted	Cytochrome P450	NA	0.00178	0.00647	0.00136	NA	-1.0524	2.4884	-0.1911
Zt09_TU_3_00731	.	Not secreted	Major Facilitator Superfamily	0.00193	0.00556	NA	0.00244	-0.2011	-0.0635	NA	0.2910
Zt09_TU_4_00055	.	Not secreted	Pyridoxal-dependent decarboxylase conserved domain	NA	NA	NA	NA	NA	NA	NA	NA
Zt09_TU_4_00056	.	Secreted	NA	NA	NA	NA	NA	NA	NA	NA	NA
Zt09_TU_4_00057	.	Not secreted	NA	NA	NA	0.00109	NA	NA	NA	-1.9137	NA
Zt09_TU_4_00289	.	Secreted	NA	NA	0.00770	0.00337	NA	NA	-0.9768	-1.7264	NA
Zt09_TU_4_00318	.	Not secreted	NA	NA	0.00476	NA	0.00930	NA	-2.5543	NA	-1.6147
Zt09_TU_5_00006	.	Not secreted	Major Facilitator Superfamily	0.02441	0.02830	0.01764	0.00416	1.1838	1.9249	-0.3417	-2.6993
Zt09_TU_5_00021	Polyketide synthase gene	Not secreted	Beta-ketoacyl synthase, C-terminal domain, Alcohol dehydrogenase GroES-like domain, Phosphopantetheine attachment site, KR domain, Acyl transferase domain, Ketoacyl-synthetase C-terminal extension, Zinc-binding dehydrogenase, Polyketide synthase dehydratase, Beta-ketoacyl synthase, N-terminal domain	NA	0.00939	0.00690	0.01565	NA	-1.3118	-0.2587	2.0129
Zt09_TU_5_00022	.	Secreted	Lysophospholipase catalytic domain	0.00087	0.00293	0.00191	0.00341	-1.4282	1.6155	0.3457	1.8513
Zt09_TU_5_00052	.	Not secreted	NA	0.01184	0.02408	0.01468	0.01669	-0.1627	0.0623	0.2287	0.3214
Zt09_TU_6_00161	.	Not secreted	Tannase and feruloyl esterase, Alcohol acetyltransferase	0.00470	0.01451	0.00881	0.00069	0.1069	0.4786	-0.7897	-2.5950
Zt09_TU_6_00163	.	Not secreted	RNA recognition motif. (a.k.a. RRM, RBD, or RNP domain)	NA	NA	NA	NA	NA	NA	NA	NA
Zt09_TU_6_00181	.	Secreted	NA	NA	0.00856	0.00774	0.00299	NA	-1.2369	0.0656	-1.3367
Zt09_TU_6_00233	.	Not secreted	Luciferase-like monooxygenase	NA	0.00285	0.00464	0.00182	NA	-0.7016	1.2058	-0.2455
Zt09_TU_7_00040	Small secreted protein	Secreted	NA	NA	0.01192	NA	NA	NA	1.5413	NA	NA
Zt09_TU_7_00426	.	Not secreted	Prenyltransferase and squalene oxidase repeat	NA	NA	NA	NA	NA	NA	NA	NA
Zt09_TU_8_00461	.	Not secreted	emp24/gp25L/p24 family/GOLD	NA	NA	NA	NA	NA	NA	NA	NA

Table S12: List of the genes found in regions highly differentiated among the two Oregon sympatric *Zymoseptoria tritici* populations

Gene ID	Protein family (PFAM) domain	Gene Ontology (GO) terms
Zt09_TU_1_00605	His Kinase A (phospho-acceptor) domain, Histidine kinase-, DNA gyrase B-, and HSP90-like ATPase, Response regulator receiver domain	GO:0000155|GO:0007165|GO:0000160|GO:0016310|GO:0016772
Zt09_TU_1_00607	NA	NA
Zt09_TU_1_00613	NMT1/THI5 like	GO:0009228
Zt09_TU_1_00614	NMT1/THI5 like	GO:0009228
Zt09_TU_1_00615	NA	NA
Zt09_TU_1_00624	NA	NA
Zt09_TU_1_00625	Protein kinase domain	GO:0004672|GO:0006468|GO:0004672|GO:0005524|GO:0006468
Zt09_TU_1_00627	NA	NA
Zt09_TU_1_01019	Pro-kumamolisin, activation domain	GO:0004252|GO:0006508|GO:0008236|GO:0006508
Zt09_TU_1_01474	NA	NA
Zt09_TU_2_00308	Glutathione S-transferase, C-terminal domain	NA
Zt09_TU_6_00188	Acyltransferase, Acyltransferase C-terminus	GO:0008152|GO:0016746
Zt09_TU_6_00676	Sir2 family	GO:0070403|GO:0006476|GO:0008270|GO:0016811|GO:0051287
Zt09_TU_9_00014	CFEM domain	GO:0008061
Zt09_TU_9_00015	MoeA C-terminal region (domain IV), Probable molybdopterin binding domain, MoeA N-terminal region (domain I and II)	GO:0032324|GO:0006777
Zt09_TU_12_00014	Fungal Zn(2)-Cys(6) binuclear cluster domain, Fungal specific transcription factor domain	GO:0000981|GO:0005634|GO:0006355|GO:0008270|GO:0003677|GO:0005634|GO:0006351|GO:0008270

Note S1: Population structure of the 123 *Zymoseptoria tritici* isolates.

Isolates clustered by geographical origin according to the PCA (Fig. 1C; Fig. S2A). Bayesian genetic clustering performed using the software STRUCTURE similarly revealed a clear genetic clustering by geographical origin (Fig. 1B). Low levels of genetic admixture were observed for K=4. However, determination of the optimal number of genetic clusters using Evanno's method (Evanno et al. 2005) showed that K=2 was the optimal clustering. These results showed that the Australian isolates were most distant from any of the three other populations (Fig. 1B). We found no evidence for genetic substructure within populations.

Evanno G, Regnaut S, Goudet J (2005) Detecting the number of clusters of individuals using the software structure: a simulation study. *Molecular Ecology,* 14, 2611–2620.
